# Historical insights at scale: A corpus-wide machine learning analysis of early modern astronomic tables

**DOI:** 10.1126/sciadv.adj1719

**Published:** 2024-10-23

**Authors:** Oliver Eberle, Jochen Büttner, Hassan el-Hajj, Grégoire Montavon, Klaus-Robert Müller, Matteo Valleriani

**Affiliations:** ^1^ Machine Learning Group, Technische Universität Berlin, Marchstr. 23, 10587 Berlin, Germany.; ^2^ BIFOLD–Berlin Institute for the Foundations of Learning and Data, 10587 Berlin, Germany.; ^3^ Max Planck Institute of Geoanthropology, Kahlaische Str. 10, 07745 Jena, Germany.; ^4^ Max Planck Institute for the History of Science,Boltzmannstr. 22, 14195 Berlin, Germany.; ^5^ Department of Mathematics and Computer Science, Freie Universität Berlin, Arnimallee 14, 14195 Berlin, Germany.; ^6^ Department of Artificial Intelligence, Korea University, Seoul 136-713, South Korea.; ^7^ Max Planck Institute for Informatics, Stuhlsatzenhausweg 4, 66123 Saarbrücken, Germany.; ^8^ Institute of History and Philosophy of Science, Technology, and Literature, Faculty I–Humanities and Educational Sciences, Technische Universität Berlin, Straße des 17. Juni 135, 10623 Berlin, Germany.; ^9^ The Cohn Institute for the History and Philosophy of Science and Ideas, Faculty of Humanities, Tel Aviv University, P.O. Box 39040, Ramat Aviv, Tel Aviv 6139001, Israel.

## Abstract

Understanding the evolution and dissemination of human knowledge over time faces challenges due to the abundance of historical materials and limited specialist resources. However, the digitization of historical archives presents an opportunity for AI-supported analysis. This study advances historical analysis by using an atomization-recomposition method that relies on unsupervised machine learning and explainable AI techniques. Focusing on the “Sacrobosco Collection,” consisting of 359 early modern printed editions of astronomy textbooks from European universities (1472–1650), totaling 76,000 pages, our analysis uncovers temporal and geographic patterns in knowledge transformation. We highlight the relevant role of astronomy textbooks in shaping a unified mathematical culture, driven by competition among educational institutions and market dynamics. This approach deepens our understanding by grounding insights in historical context, integrating with traditional methodologies. Case studies illustrate how communities embraced scientific advancements, reshaping astronomic and geographical views and exploring scientific roots amidst a changing world.

## Teaser

An unsupervised ML model analyzes historical sources beyond human capacities through an atomization-recomposition approach.

## INTRODUCTION

The European early modern period has traditionally been regarded as the cradle of modern society, particularly highlighting advancements in science and technology. Science itself has often been portrayed as a progressive process, culminating in a scientific revolution that propelled Europe into modernity. The works of alleged heroes of science such as Nikolaus Copernicus, Galileo Galilei, and Johannes Kepler were given special significance in this process. Their publications were frequently and sometimes still are regarded as pivotal moments, encapsulating the essence of the astronomical revolution of this era (*1*–*9*). These views, largely still dominating public reception, are rightly being challenged today.

Recent history of science is beginning to overcome its Eurocentrism and adopt a more differentiated view on the processes that led to the emergence of science. In his influential work, *The Structure of Scientific Revolutions* (1962), Thomas Kuhn had already emphasized the role of scientific paradigms, moving away from focusing solely on the contributions of a few selected individuals to viewing scientific progress as a collective achievement of the broader scientific community (*10*). Even earlier, scholars like Braudel (*11*) and Bloch (*12*, *13*) aimed to bridge the spatial and temporal gaps between well-studied singular events by analyzing a broader collection of sources. Today, modern approaches, informed by this historiographical legacy, delve into a much broader array of historical sources than before to gain a more comprehensive understanding of the intellectual context within which the celebrated “heroes” of science worked and developed their novel insights. The development of science is no longer perceived as a linear progression but as a multifaceted process shaped by a variety of factors operating across different temporal scales (*14*–*16*). However, a substantial practical limitation obstructs these new approaches to the history of science and history in general: The sheer volume of available sources surpasses our current capacity to conduct historical investigation with a comparatively limited pool of trained historians.

Machine learning (ML), specifically deep learning (*17*–*20*), has established itself as a powerful way of making inferences from data at scale. A variety of historical studies could take advantage of the recent successes of deep learning in vision and language (*21*–*25*). Well-curated image datasets and benchmarks of historical sources (*26*–*30*), combined with pretrained ML models for computer vision such as U-Net (*31*), YOLO (*21*), or CLIP (*32*), enabled to extract relevant visual elements such as illustrations, drawings, or images and place them in relation to their accompanying texts on the level of a whole corpus (*29*, *33*–*35*). Likewise, recurrent neural networks (RNN) and, more recently, transformer-based architectures have enabled significant progress in optical character recognition and handwritten text recognition of historical content (*27*, *36*–*39*). Beyond mere data exploration and extraction, Assael *et al.* (*40*) proposed a sequence-to-sequence RNN to reconstruct ancient Greek inscriptions, which was later followed by a transformer-based architecture not only to restore inscriptions but also to generate local insights about their provenance and dating (*41*). Other ancient languages also benefited from deep learning approaches, such as Latin (*42*), Akkadian (*43*), and Hieroglyphs (*44*). Last, Explainable artificial intelligence (XAI) (*45*–*50*) reveals aspects of the internal processing of ML models, putting visual and textual features in relation to predicted outputs and thus enabling a novel, insightful exploration of humanities datasets (*51*–*53*).

Despite these advances, the analysis of historical data at large, including the numerical tables our work focuses on, presents very unique challenges from a ML perspective. In particular, higher-level interpretations by historians that shift from the formal assessment of individual units to semantic, corpus-wide assessments cannot rely on the availability of labeled datasets. Furthermore, general historical data are characterized by extensive heterogeneity and nonstationarity. These factors make end-to-end approaches particularly challenging, as they require a substantial amount of data that are now not available for the great majority of relevant tasks.

In this work, we demonstrate that these challenges can be addressed with our proposed “atomization-recomposition” approach. The approach rests on a data-efficient modeling of task-relevant features, where historical sources are decomposed into a collection of elementary units (the “atoms”) before being recomposed. This allows us to rely on intermediate atom-wise predictions, for which labels can be readily acquired. Furthermore, our approach integrates XAI for verifiability, transparency, and human interpretability. Overall, it enables a dataset-wide and robust processing of the historical content and provides accurate similarity predictions and groupings of historical data. In the following, we demonstrate the practical usefulness of our approach for a concrete historical research study.

In particular, we focus on the core astronomical knowledge of the early modern period, i.e., the set of widely accepted theories, methods, and results in the field of astronomy. A prime source for reconstructing this extensive core knowledge are university textbooks, which informed the broader student population and intelligentia (*54*). Historians have previously shown interest in textbooks (*55*, *56*). However, a comprehensive analysis of larger collections of this particular type of source has remained elusive due to the great amount of available material. Our research is uniquely poised in this context, as we leverage the “Sacrobosco Collection” (*57*–*61*) (section A.1 of Supplementay Materials and Methods). This very large and substantial thematic collection encompasses textbooks introducing geocentric astronomy to students across Europe from the final quarter of the 15th century up to 1650. During this period, the pre-Copernican geocentric worldview was a highly dynamic scientific field in which innovations quickly entered the core knowledge and left repercussions even at the level of the textbooks (*59*–*62*).

The collection contains approximately 359 editions of different textbooks, totaling around 76,000 pages of scientific content. These books were published starting in 1472, the year of the first edition (and of the first ever printing of a scientific, mathematical text). The year 1650, in which the last edition of the corpus was printed, marks the end of the slow decline of geocentric astronomy initiated almost a 100 years earlier by Nikolaus Copernicus, whose *De revolutionibus orbium coelestium* of 1543 introduced a mathematical system based on a heliocentric worldview. Assuming an average print-run of ~1000 copies, the Sacrobosco Collection can be considered as representative for about 350,000 textbooks that were circulating and used in Europe during the period considered (*58*, *63*, *64*) (section A.1.1 of Supplementary Materials and Methods).

Our study specifically addresses the mathematical education and culture had by students and the educated populace, i.e., the potential readers of the textbooks in our corpus. This entails understanding where this knowledge originated, the motivations behind its dissemination, and the modes of its circulation.

Computational astronomical tables are a central element of the mathematical apparatus of early modern astronomy. These tables can be understood as the sequential representation of the input and output values of mathematical relations, akin to equations. Before the advent of formulaic algebraic language toward the end of the period considered, this method was the predominant way to express mathematical relations, the meanings of which were described in the texts associated with the tables (*65*, *66*).

We investigate the astronomical tables as a proxy for the underlying mathematical knowledge, its transformation, and its dissemination. This investigation focuses on a collection of tables that must first be identified within the corpus, with their meanings being entirely unknown. Such a collection differs from curated tables, such as those containing observational data and chronologically ordered, which are compiled from the outset, often within an archive, and have inherent semantic attributes. In the type of investigation we undertake, historians would have to initially ascertain whether each table within the corpus represents new information or merely repeats existing knowledge. This would involve comparing each table against the others in the corpus to see whether they represent the same fundamental knowledge or not. Particularly with complex computational tables (fig. S7 for an example), only a few experts in early modern astronomy can accurately make these comparisons. Moreover, judgments can be complex, as tables may appear similar but be fundamentally different or vice versa (section A.1.4 of Supplementay Materials and Methods). The sheer volume of required table comparisons to track innovations, their spread, and their disappearances for a corpus of the size of the Sacrobosco Collection renders such a close reading analysis based only on traditional methods of the historian impossible. Hence, to support such investigations, computational methods can greatly streamline the steps necessary to identify the numerical tables in the corpus, group them according to a semantically meaningful similarity, and analyze the dynamics of their development across space and time.

The editions analyzed in this work come from different times and from different places and were frequently produced following very different standards. The heterogeneity of these printed books and the tables they contain is compounded by the intertwining effects of the processes of scientific knowledge transformation, the development of printing technology, and the mechanisms of the academic book market (*58*, *67*–*70*), each of which contributes differently to the diverse sources of data variability (sections A.2 and A.4 of Supplementary Materials and Methods).

Assessing the complex similarity structure of the numerical tables contained within the corpus poses challenges for both trained historians and conventional ML approaches, namely, end-to-end training and the utilization of pretrained models. While end-to-end approaches may be feasible for higher-level historical tasks, they rely on a substantial amount of data that is typically not available.

The acquisition of a labeled dataset would require a historian to perform many detailed analyses or similarity assessments of complex tables. This approach does not scale well, especially considering that the corpus contains approximately 10,000 pages with numerical tables (section A.1.4 of Supplementary Materials and Methods). Furthermore, the application of conventional ML approaches to this task seems unfeasible due to the high heterogeneity of these numerical tables (sections A.2.2 and A.8 of Supplementary Materials and Methods).

Our proposed atomization-recomposition ML approach efficiently addresses the labeling and heterogeneity challenges described above. In the case of numerical tables, we define the “atom” to be an individual digit. This allows us to decompose the ML approach into two main steps: first, building a ML detector for these digits (for which labels can be acquired handily) and then recombining the detected digits into more informative sequences of two digits or “bigrams.”

Besides the advantage of requiring only a limited number of single-digit labels to ensure their robust detection, this approach also allows for the detection of features that do not occur in the training data. For example, the bigram “25” could be detected on test pages even when the training pages contained only the bigrams “12” and “51.” Last, these spatially resolved bigrams can be pooled into a “bag-of-bigrams” that subsumes the content of an entire numerical page into a hundred-dimensional vector. Our atomization-recomposition approach is related to common practices in other domains, such as the vector space model and bag-of-words representations in Natural Language Processing (NLP) (*71*) or “visual words” in image classification (*72*–*74*). Our instantiation with historical data, however, innovates by addressing technical challenges specific to low-resource settings, such as the distinction of tabular from nontabular pages, the pixel-level detection of individual digits and their classification amidst high heterogeneity, the data-efficient recomposition of those digits into bigrams, and their page-wide aggregation (section A.3 of Supplementary Materials and Methods).

We validate our “bag-of-bigram” representation of tables and the implied similarities, using both nominal accuracies and XAI techniques such as Layer-Wise Relevance Propagation (LRP) and Second-Order Layer-Wise Relevance Propagation (BiLRP) (*75*, *76*). The BiLRP technique verifies that similarity predictions are grounded in meaningful pixel-level patterns. By leveraging the similarity predicted by our model over the entire corpus, our approach reaches its full potential, enabling previously impractical or even impossible historical investigations. Specifically, the examination of the geo-temporal circulation of the numerical tables provides insights into the widespread dissemination of mathematical education and culture in the frame of astronomy which otherwise remains obscured by an enormous, previously unanalyzable volume of astronomical tables.

Our approach allows not only for a systematic extraction of data-driven insights in large corpora, it also provides an example for the quantification of historical processes at scale. It thus aids in making more informed selections of historical source material which can then be analyzed using conventional methods of historical inquiry. The historical analysis presented on early modern mathematization serves as an example of how historical disciplines can benefit from ML and XAI methodologies. These methodologies aid in identifying case studies and assist in close-reading analysis of individual sources.

## RESULTS

**Fig. 1.**
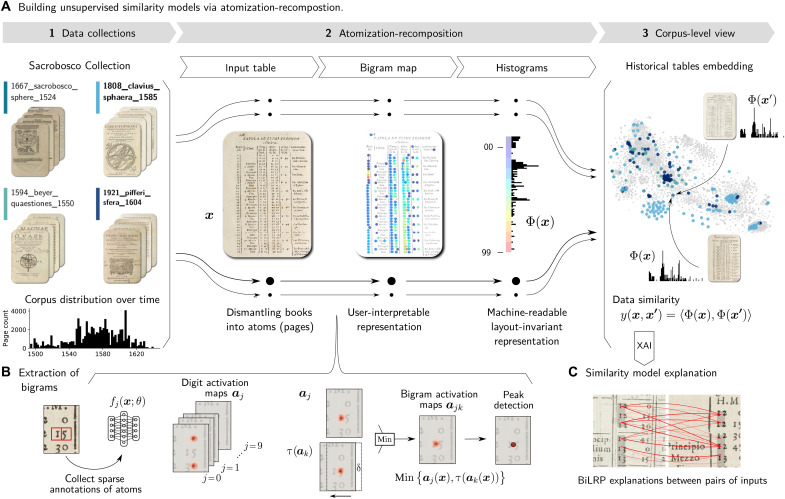
Atomization-recomposition framework for model learning under sparse annotation settings. (**A**) Overall computational workflow starting with an unstructured collection of books (Sacrobosco Collection), which are atomized into tables and single digits that a ML model can detect, recomposed into user-interpretable bigrams, and lastly into histograms that enable dataset-wide unsupervised ML-based analyses. (**B**) Details of the bigram extraction in the atomization-recomposition workflow. A neural network digit recognition model activates where digits are found in the input image, and the resulting digit activation maps are recomposed through specific operations into more task-specific numerical bigrams (section A.3 of Supplementary Materials and Methods). (**C**) The similarity scores on which ML-based analyses operate are verified via XAI, specifically the BiLRP technique (*76*), which highlights how the similarity scores arise from the pixel representation (section A.5 of Supplementary Materials and Methods).

Starting from the Sacrobosco Collection and seeking to achieve a homogeneous representation of such a corpus with a focus on the numerical tables, we proceed with the proposed atomization-recomposition approach. Using a ML classifier, we extract tables versus nontable pages from a few ground-truth annotations, enabling us to reliably extract the ~10,000 pages bearing tables contained in the corpus—we refer to this selection of pages as the Sacrobosco Tables corpus (section A.7.2 of Supplementary Materials and Methods). This first step enables us to reduce the heterogeneity arising from nonnumerical content. A neural network, shown in Fig. 1B, extracts spatially resolved individual digits from the numerical table pages. From approximately 2500 annotations, a single-digit detection accuracy of 96% could be reached, thereby achieving at minimal cost a further reduction of data heterogeneity, for example, with respect to font size, print quality, and nonnumerical elements such as the tables’ vertical and horizontal lines.

Building on this atomized representation of the Sacrobosco Collection, we proceed with recomposition, specifically, the meaningful aggregation of atoms into representations of numerical tables that are interpretable and have predictive abilities. Our first recomposition step consists of augmenting the detected individual digits with digit bigrams, which we achieve technically by juxtaposing pairs of activation maps, applying a slight horizontal shift, and looking for the intersections of the activation peaks. This processing step is illustrated in Fig. 1B, and the details of the procedure are given in section A.3 of Supplementary Materials and Methods. The result is a “bigram map” that can be rendered as a color map (an example is shown in Fig. 1). We observe that the latter is particularly interpretable for the user: Increasing numerical series appear as color gradients, and numerical anomalies easily stand out.

**Fig. 2.**
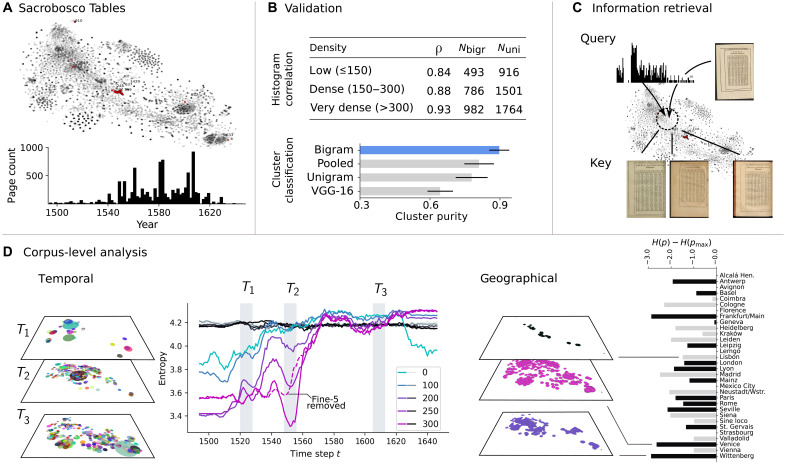
Extracting historical insights from bigram histograms. (**A**) Visualization of the Sacrobosco Tables corpus using t-SNE. A set of hand labeled, semantically identical tables providing the position of the Sun against the Zodiac over the course of the year is shown in red. The temporal distribution of printed table pages is shown below. (**B**) Validation of different table representations and Pearson correlation scores for different digit densities (number of digits per table page). (**C**) By providing query histograms or reference pages, our approach is able to generate a set of key candidates of tables that are identical or very similar to a given query table. (**D**) Left and middle: Transformation of knowledge over time as measured via the entropy of the cluster membership distributions (counts of tables within each cluster). Gray to black lines represent a random bigram embedding baseline, while the colored lines represent data from the Sacrobosco Collection. Different colors indicate filtering thresholds that are based on digit density per page, such as pages with at least 100 digit features. Resulting clusters are visualized for three distinct time intervals using the same t-SNE coordinates used for the full corpus. The disk diameter of each cluster is scaled according to its number of members. Notably, we detect an entropy drop for tables with dense numerical content between 1540 and 1560. This drop vanishes upon excluding the Fine-5 group, a subset of tables found in editions authored by Oronce Finé, that we have identified as the primary driver of the change in entropy. Right: Examining the geographic distribution of knowledge across print locations, sorted alphabetically, using entropy. Low-output cities that have printed less than 100 tables are colored in light gray, and t-SNE visualizations for three selected cities are shown.

A second stage of recomposition then converts, via spatial pooling, this human-readable map representation into a lower-dimensional bag-of-bigrams, which takes the form of a histogram that is invariant to the exact table layout. We validate the resulting histograms on a diverse subset of fully annotated table pages (section A.4.1 of Supplementary Materials and Methods) and achieve average Pearson correlations to the ground-truth histograms ranging from 0.84 for tables of low digit density to 0.93 for high density tables, as shown in Fig. 2B. The compactness of the bag-of-bigrams representation effectively addresses the curse of dimensionality in downstream applications while remaining highly predictive. We assess the performance of different table page representations in identifying clusters of identical table pages, with an exemplary visualization of the different page representations shown in fig. S20. We find that our proposed bag-of-bigrams representation is most effective for retrieving correct cluster members, reaching 90% purity, compared to 81% for a direct pooling of bigram activations (pooled), 78% for single-digit summaries (unigram), or 64% for a pretrained deep neural network representation from Visual Geometry Group 16 (VGG-16) (see also Fig. 2B). In addition, using XAI, specifically the BiLRP technique (*76*), we can verify that the similarity predictions built on our bag-of-bigrams representation are stably grounded in the numerical content (Fig. 1C), whereas unspecific methods based on pretrained models produce much less interpretable results (section A.5 of Supplementary Materials and Methods). While we evaluate our atomization-recomposition on numerical tables, which are important information carriers for our subsequent historical analyses, we emphasize that our approach could in principle be extended to other aspects of historical documents, such as structured mathematical diagrams (section A.8 of Supplementary Materials and Methods), by choosing appropriate atomization and recomposition steps.

### Corpus-level historical insights and case studies

Our approach allows (i) for historical investigations on a general, corpus level as it makes it possible to trace and analyze the geotemporal circulation of the numerical tables in the entire corpus and (ii) for the identification of particularly interesting clusters of similar tables which guide an informed selection of specific case studies to be ultimately analyzed through standard close-reading. The results of the corpus-level analysis are described below, along with the identification and investigation of two relevant, mutually interconnected case studies.

On a corpus level, we demonstrate that the process of astronomy’s mathematization, codified in textbooks and taught at the European universities, occurred alongside a process of accelerating dissemination of mathematical knowledge that took place during the last decades of the 16th century. This acceleration was ignited and fueled mainly by the competition between two key entities: the French Royal Chair of Mathematics and the Collegio Romano, the principal mathematical division within the Jesuit order (*77*) (section B.1.1 of Supplementary Text). Spreading mathematical knowledge was among the main goals of both institutions.

This process exhibits a dynamics that, on closer inspection, turns out to be caused to a large extent by the necessity to adhere to early modern marketing rules for academic prints. These rules required the rapid introduction of scientific works in various formats to the market, with multiple editions of each work released in close temporal proximity to one another (*78*–*80*), resulting in a high variability in printed pages with numerical content over time (Fig. 2A). The most relevant episodes of such high-frequency publication and republication of mathematical content occurred within a 5-year timeframe around 1550 and involved Oronce Finé, who was then the French Royal mathematician (Fig. 2D) (*81*). This publishing pattern may be attributed to market mechanisms that incentivized publishers to release multiple new editions within short timeframes (see section B.1.1.1 of Supplementary Text).

The accelerated circulation of mathematical knowledge represented in the corpus of textbooks was accompanied by a process of homogenization, where scientific works increasingly offered the same mathematical approaches (sections A.1 and A.1.2 of Supplementary Materials and Methods and section B.1.1 of Supplementary Text). By measuring the entropy of cluster membership vectors that represent the number of table pages in each cluster as presented in Fig. 2D, we show which places of print production contributed to this phenomenon most and which did so to a lesser extent. Low entropy scores indicate high redundancy, characterizing a printing process that repeatedly prints the same material, while higher scores signify a more diverse range of printed contents. The resulting entropy scores indicate that the mathematical knowledge presented in treatises produced, for instance, in post–Reformation Wittenberg is particularly homogeneous, presumably due to the political control exerted over scientific education in the city during this period (*82*–*84*). Similarly, the low entropy score for Frankfurt am Main suggests a high degree of content homogeneity. A closer look at the sources shows that this is because out of the 17 editions produced in the city, 13 are reprints of only two distinct editions (section B.1.1.2 of Supplementary Text).

The corpus-level analysis reveals instances where the pattern of spread of mathematical knowledge, embodied in the tables, deviates from broader established trends in interesting ways, diachronically, synchronically, or even semantically. This enable us to make informed decisions about specific case studies (section B.1.2 of Supplementary Text). To facilitate such studies, which allow an in-depth exploration of a particular phenomenon across its entire temporal and spatial trajectory of transformation, we provide a tool to identify clusters of tables identical and similar to one table selected by a domain expert (section B.1.2 of Supplementary Text).

**Fig. 3.**
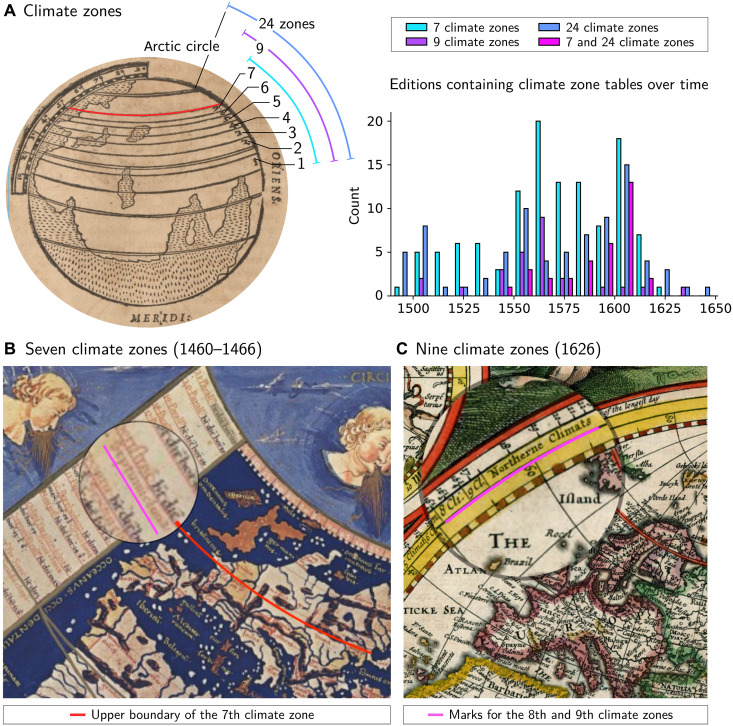
Historical case study 1. (**A**) Early modern illustration of climate zones depicting the original 7 climate zones and adapted to show the later addition of 9 and 24 climate zones. The temporal distribution of the number of editions containing these tables is shown in histogram bins of 10 years. From (*101*) (sign. F-5v). Hochschul- und Landesbibliothek Fulda. (**B**) World map, as conceived in the Hellenistic era by Ptolemy, and whose oldest known exemplar was drawn during the 15th century by following Ptolemy’s list of coordinates and metric. The seventh climate zone clearly excludes all regions north of Paris, including present-day Great Britain (the northern border of the seventh climate zone is delineated by a superimposed red line. The superimposed fuchsia line underlines the text “Septumm climatum”). From (*102*). (**C**) Robert Walton’s world map drawn in 1626. It includes all recently discovered territories on Earth but considers only nine climate zones as worth explicit mention. The ninth climate zone does include England but was originally introduced to include Wittenberg. Further zones toward North are only generically named. (The superimposed fuchsia line underlines the text “8 Cli./9. Cl./Northerne Climats”). From (*103*). Courtesy of Stanford University Libraries.

**Fig. 4.**
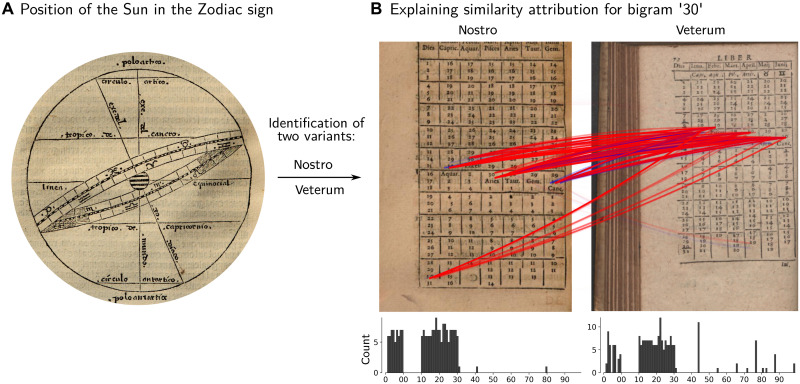
Historical case study 2. (**A**) Illustration displaying the orbit of the Sun (ecliptic) on the Zodiac subdivided into the twelve signs. From (*104*) (sign. b-IIII-4). Augsburg, Staats- und Stadtbibliothek. Uniform Resource Name: urn:nbn:de:bvb:12-bsb11218245-6. (**B**) Examples for two types of Sun-Zodiac tables: the ancient (veterum) and the 16th century (nostro) variations. The prediction of the similarity model is made explainable by highlighting the most relevant feature interactions, here using the bigram “30” as an example. It is clearly visible that the position columns are shifted by a fixed number of days.

In Figs. 3 and 4, we present two case studies selected through an integrated approach that combines data-driven cluster identification with expert domain knowledge (section B.1.1.3 of Supplementary Text). The first, which corresponds to the spatially and temporally most extended cluster, is dedicated to the method for geometrically subdividing the Earth’s surface from the equator to the poles based on the length of the solar day. The second is concerned with the calculation workflow necessary to retroactively predict the position of the Sun on the Zodiac during classical antiquity (sections B.1.2.1 and B.1.2.2 of Supplementary Text, with individual examples).

Since antiquity, the known world was considered as divided into habitable and inhabitable parts. The inhabitable parts were not considered to be entirely devoid of people but generally held to be inhabitable because of the hard conditions they presented for life. The habitable parts, covering roughly the longitudinal area of Europe and extending from North Africa northward to include Paris, were further traditionally divided into seven “climate zones.” A climate zone (land strips bordered by parallels to the equator) was defined on the basis of the length of the solar day on the summer solstice in those areas. This conception was fundamental for a variety of scientific disciplines, such as medicine, and continued to be taught until at least the mid-17th century (*85*). However, the early modern journeys of explorations had revealed that the ancient conception of the habitable part of the world was too limited (*86*). This situation is reflected in the sources under consideration, which display different types of climate zone tables: one for seven zones shown in Fig. 3B and another that encompasses the entire planetary surface from the equator up to the polar circle and thus conceptualizes 24 zones. In addition, a third transitional type of climate zone table, listing nine zones, could be identified (see Fig. 3C). Full maps are shown in figs. S44 and S45 in Supplementary Text.

By identifying and comparing a large number of relevant tables, we were first of all able to track the dissemination of the pertinent knowledge in detail over the 178 years under consideration as presented in Fig. 3A for the different variants of climate zone tables. We could establish that, contrary to what one might expect, the spread of the modern conception of 24 zones was unexpectedly not detrimental to the ancient one (section B.1.2.1 of Supplementary Text). The opposite is the case: The success of the innovation was largely dependent on its link to the traditional, ancient, and authoritative concept and associated worldview. The peak in the dissemination of the table representing the new conception of 24 zones can primarily be attributed to editions that also included the old table listing the traditional seven zones, as shown in Fig. 3A, where the publication frequency per decade is plotted for the editions containing 7, 9, and 24 climate zone tables and for those editions concurrently containing both 7 and 24 climate zone tables.

Second, by accurately assessing the similarity within the cluster of the climate zone tables, our approach enabled the identification of a third variant of climate zone tables. This variant initially expanded the old view but only so far as to incorporate European regions at higher northern latitudes, specifically to include Wittenberg by adding two zones as highlighted in Fig. 3C (visualizations that displays the spread of the climate zone are available in the Supplementary Materials). Although the dissemination of this conception of nine zones remained limited in both time and space, it represented a notable break from the traditional view. Historians of the early modern period have previously shown some interest in the use of the climate zone concept in fields like medicine. The concept itself, however, they have regarded mostly as a stable and inert knowledge structure inherited from antiquity. Against this view, we have been able to demonstrate that the Age of Discovery’s far-reaching transformations did not dismiss the concept of climate zones. On the contrary, the authority of the older conception was even used as a vehicle for innovation. The latter, however, only expanded the number and geographic validity of climate zones. This transformation may have been perceived by historical European actors as a validation of their scientific endeavors during the Age of Exploration, contributing to the context in which science later became a central element of Europe’s cultural identity (section B.1.3 of Supplementary Text).

The second case study presented in Fig. 4 (A and B) focuses on a scientific specialization, no longer extant, that closely connected mathematical astronomy and history. Starting from the 13th century, when Europeans created the epochal subdivision between antiquity, the Middle Ages, and the new epoch in which they were living, frantic activity began that aimed to reconstruct an exact chronology of ancient events (*87*, *88*). This was because, from the perspective of the day, antiquity represented the epoch during which the pinnacle of civilization and knowledge had been reached. In antiquity, the connection between the calendar and the Sun’s position within the signs of the Zodiac was already well-established. As a result, by providing the positional values for the Sun, it was possible to calculate the day of the year and vice versa. Consequently, in ancient Greek and Latin works, descriptions of events are often accompanied by specific astronomical observations that can be linked to the position of the Sun in the Zodiac.

After Philipp Melanchthon, one of the founding fathers of the Protestant Reformation, had urged young students to study astronomy in 1531 and 1538, warning that without it the history of humanity would be mere chaos (*89*, *90*), a particular scientific specialization emerged. Aimed at providing precise dates for ancient events, this field of study persisted into the 19th century, especially within German universities. Mathematically, the required calculations were challenging both because of the historical changes of the calendar systems and the precession of the equinoxes, which itself was not yet fully understood in the 16th century (*91*). Focused on this historical case, our approach provided us with the necessary means to identify and select relevant historical material which allowed us to investigate one of the first steps of a broad phenomenon of dissemination of mathematical culture in the framework of astronomy instruction at the universities.

First of all, we have been able to show that the values of the position of the Sun against the ecliptic were transposed into a handy table for the students for the first time in 1543 and have established that this table was printed and used only in Northern Germany and France (visualizations that display the spread of the nostro tempori tables are available in the Supplementary Materials). Second, and more relevantly, we were able to identify another table, which essentially provides the same information but pertaining to the position of the Sun over the course of the year for ancient times, called the veterum poetarum temporibus accommodata table. To communicate this information, a new table is required, since the position of the Sun relative to the zodiacal signs for a given date of the year changes over time. While the annual change is minimal, the change accumulates to a noticeable difference if longer time periods are considered. This new table is similar, but not identical, to the original handy table and therefore serves to display directly the position of the Sun as it was observed by the ancient writers. The new type of table was first conceived in Wittenberg and was apparently created to simplify the calculations otherwise required to convert the current (16th century) position of the Sun at a given day into its position on that day in antiquity, which was necessary to establish a connection to the calendar. A dynamic visualization of the spread of the Sun-Zodiac table for the ancient authors can be accessed via section B.1.2.2 of Supplementary Text. The focus on an astronomy-based reconstruction of ancient chronicles shows the early modern European interest not only in establishing own intellectual roots but also a specific chronicle that, because of the mathematical workflow to calculate it, could gain a high level of consensus and leave less space to interpretation. At the same time, the process of homogenization of the content of astronomy instructional textbooks was ongoing, driven largely by the imitation of textbooks conceived and produced in Wittenberg (section A.1.2 of Supplementary Materials and Methods). Through this process, this specific type of mathematization of astronomy, a project driven by the Reformers in particular Philipp Melanchthon, eventually turned into a knowledge of the ancient past that was shared all over Europe whereby it was not so much the chronicle that was shared but the knowledge to calculate it. As astronomy was becoming an element of a European cultural identity during the age of the journeys of exploration (section B.1.3 of Supplementary Text), then sharing the same calculation workflow to generate chronicles of the ancient events on a continental level likely reinforced the role of science as an identity-shaping factor.

## DISCUSSION

The present study has illuminated both qualitatively and quantitatively how mathematical knowledge as taught in the early modern universities in Europe has evolved in a context of institutional competition. This competition seems to have fostered a scientific knowledge sharing process in Europe even while, as is well known, the region was being fragmented by religious and political currents.

The pattern along which the conception of historical climate zones changed (from 7 to 7 + 2 and eventually to 24 climate zones) allows us to hypothesize that the emergence of a shared science in continental Europe, at least as the generally educated populace is concerned, was coupled to the increasing perception of an expanding world, albeit of one pivoting around Europe.

The computation of the position of the Sun with respect to the Zodiac for dates in antiquity, moreover, reflects the emergence and spread of a shared desire of a society to establish its own intellectual roots. Given the relevance acquired by the Wittenberg textbooks in astronomy all over Europe (section A.1.2 of Supplementary Materials and Methods), the concerted effort of the Wittenberg Reformers to accurately reconstruct the chronology of historical events of classical antiquity turned into chronicles of the ancient epoch. These chronicles could achieve wide consensus all over Europe exactly because they were based on a shared computational workflow, leaving little room for interpretation and thus being prone to creating agreement.

The development of a global cultural perspective in Europe, coupled with the emerging need to establish one’s own historical roots, contributed to the creation of a shared intellectual background against which the European scientific and cultural identity later evolved. Considering the high mobility of university students during the early modern period in Europe (*92*), and given the rate of dissemination of basic astronomical knowledge into vernacular communities, such as those involved in navigation (*93*), it is suggested that astronomical core knowledge became one of the fundamental cornerstones of what Benedict Anderson defined as Latin Elite community, a trans-regional community built on the basis of book printing and knowledge circulation (*37*–*46*, *94*) (section A.1.2 of Supplementary Materials and Methods and section B.1.3 of Supplementary Text).

The current investigation could be further extended by including, in addition to textbooks, works that were associated with the research frontiers of the time (*54*). In this way, the relation between the dissemination of a broad mathematical culture and those disruptive works usually associated with the idea of an astronomical revolution during the early modern era could be studied in more detail.

Moreover, by broadening the timeframe, for instance by including more recent sources, the transformation of mathematical knowledge could be investigated as it transits from the early modern tabular expression of mathematical functional relations to the more modern, formula-based one. By broadening the geographic scope, the same phenomenon could be investigated in a global perspective, potentially allowing for a quantitative assessment of the process of European intellectual colonization. Such spatial and/or temporal extensions of the source base would first require well-curated datasets of the relevant sources.

However, it is crucial to acknowledge the general limitations posed by data-driven approaches, including methodological aspects related to data quality and quantity, as well as model interpretability (section A.8 of Supplementary Materials and Methods), and challenges of generating ML-assisted historical insights (section B.1.1.3 of Supplementary Text). These factors need to be addressed to effectively generate research hypotheses. In our atomization-recomposition approach, we have demonstrated how these challenges can be mitigated through efficient modeling embedded within a process of scrutiny, independent testing, and thorough model evaluation. This evaluation incorporates XAI to make the underlying ML inference processes transparent and verifiable (see section A.8 of Supplementary Materials and Methods).

The scarcity of data and labels presents a central challenge, limiting the ability of ML approaches to capture the full richness of their sources by focusing on specific aspects of the data. In our historical use case, we have found that bag-of-bigrams offer a sufficiently complex representation to aid historians in corpus-wide examinations.

Only after carefully addressing these methodological challenges can emerging hypotheses be further pursued based on established methods in writing history: hypothesis-driven research. This is precisely the path that we have followed.

While this ambitious vision presents numerous challenges, we would emphasize that computational astronomical tables from the early modern period are exceptionally intricate sources that demand profound expertise for analysis. We have demonstrated that such analysis can be substantially augmented by ML methods. Therefore, we would like to express optimism that our general approach can be adapted and applied to other historical questions and sources.

The integration of humanities and ML technology needs to be problem specific and highly interwoven between the disciplines. Through close interaction between these two fields, a virtuous cycle of scholarly dialogue can be achieved, one that fosters innovation, insight, and meaningful advancement. In our study, the challenge of the sparseness and heterogeneity of historical data was solved by applying a general atomization-recomposition approach, highlighting how specific domain challenges can inform the development of ML methods. However, traditional humanities approaches continue to play an important role and can be effectively applied alongside new techniques, as this work effectively demonstrates.

The ultimate goal of which this work is a part is to develop an AI-based assistant capable of facilitating an accelerated science lab for in-depth historical research, interpretation, and reconstruction. This lab would streamline the research process by using ML to generate genealogies between historical sources, thus aiding in the selection of relevant sources from the vast pool of unexplored material before historians conduct close reading analyses. These selections would be guided by trend analyses, similar to those conducted in this study. The overarching mission of the lab is to promote a deeper and more comprehensive understanding of our historical origins.

## MATERIALS AND METHODS

### Data

The Sacrobosco Collection (*95*) represents the complex edition history of the astronomy textbook *De sphaera* of Johannes de Sacrobosco and consists of a corpus of 359 early modern printed editions, amounting to roughly 76,000 pages of material (*62*). These books were used at European universities for the introduction to the study of astronomy and geocentric cosmology, a mandatory component of the first curricular year. The dates of the editions of the corpus range from 1472 to 1650. This corpus enables the study of important historical questions, such as of the evolution and homogenization process of knowledge on cosmology.

#### Table pages

From all the pages of the Sacrobosco Collection, we select 9793 pages bearing one or more numerical tables, which we submit to the table similarity workflow as the Sacrobosco Tables dataset. By numerical table we mean any tabular arrangement of data in our corpus which has at least one column with (predominantly) numerical content. We specifically exclude tables of content and book indices. The preselection was supported by an off-the-shelf Convolutional Neural Network (CNN) (VGG-16 (*96*) trained to classify pages as bearing such numerical tables or not. The output of this CNN was checked down to a low probability of the assignment of a page as bearing a numerical table. Because of the human postprocessing, the list of of pages with numerical tables should have close to perfect precision and a very high recall. A list of all pages with numerical tables is provided as sphaera_tables_9793.csv (see folder data/corpus in code.zip), and the trained model instrumental in establishing this list is provided as sphaera_tables_classifier.h5 in folder data/trained_model via https://doi.org/10.5281/zenodo.10933231. The digital images of the pages that we refer to as the Sphaera Tables dataset can be obtained at sacrobosco_tables.zip via https://doi.org/10.5281/zenodo.10933231.

#### Preparation and acquisition of ground-truth

We have prepared four different ground-truth datasets to train and test our model at different processing stages: single digits and nondigit content to train the recognition model, fully annotated numbers to test the digit recognition and the bigram expansion, and Sun-Zodiac pages to evaluate the table similarity model. These sets are provided as numerical_patches.csv, contrast_patches.csv, digit_page_annotations.csv, and sun_zodiac.csv in the code and data repository (see folder data/training_data in code.zip accessible via https://doi.org/10.5281/zenodo.10933231).

##### Single digits

. To capture the nonstandardized print types that occur in historical corpora, we have selected a subset of important printers and for each have annotated five individual number patches from five different pages that contain numerical content, resulting in a dataset of 2494 annotated numbers. From this, a dataset containing a diverse set of 5208 single digits is created. We also have added contrastive nondigit patches that contain text, illustrations, or geometry from nontable pages.

##### Fully annotated numbers

. We have selected 11 pages and annotated each single digit contained on the pages with a bounding box. In addition, we have marked whether the individual digit is the first and/or the last digit of a number. This information makes reconstructing all numbers and thus also all bigrams contained on these pages straightforward. The annotated pages have been selected to cover a wide spectrum of different manifestation of numerical content in terms of writing direction, fonts, font sizes, the density of digit placement on the page, etc.

##### Sun-Zodiac pages

. To evaluate to what extent our approach can reproduce the salient relations between the tables in our corpus, we have chosen the Sun-Zodiac tables, which give the positions of the Sun relative to the signs of the Zodiac in degrees for each day of the year. This table is well-suited for evaluating our approach. The table’s layout varies across its appearances in our corpus, with each layout partitioning the full table differently. In some cases, the entire table is contained on one page; in other books, it is distributed over as many as nine pages. The table only comprises numbers from 1 to 31 per its Zodiac content (a maximum number of 31 days per month and 30° per sign of the Zodiac). The table thus populates only a subspace of the feature space that we exploit for our similarity assessments. Since this subspace is more densely populated than would be expected with a uniform distribution of the data over the entire feature space, this table is particularly difficult to discriminate with our approach, making it a good test case.

In our corpus, we find two variants of the Sun-Zodiac table: tables for the times of the ancient poets (writers) (veterum poetarum temporibus accommodata) where the Sun is 16° into Capricorn on the first of January and tables for “contemporary” times (nostro tempori) where, on the first day of the year, the Sun has advanced 3° and is located 21° into Capricorn. This difference essentially amounts to a shift of the columns listing the days of the year with respect to columns giving the angular locations, and thus, from the perspective of our similarity model, these two variations represent the same (more abstract) table.

We have identified 68 instances of the Sun-Zodiac table which cover a total of 250 pages in the corpus. A list of the pages containing the different versions of the Sun-Zodiac tables is provided as sun_zodiac_pages.csv along with a ground-truth histogram for the digit-features distribution of a prototypical, i.e., noise-free and complete, Sun-Zodiac table that is provided as sun_zodiac_hist.csv (see folder data/corpus in code.zip accessible via https://doi.org/10.5281/zenodo.10933231).

##### Climate zone table pages

. We have also collected a subset of material that is concerned with climate zone tables, which divide the surface of the “inhabited” world into zones that can be defined by the length of the solar day. The tables served as an indication of the overall meteorological conditions, which were in turn determinant information in the framework of Medieval and early modern medicine. We find three different principle variants of climate zone tables that use either 7, 9, or 24 zones. The 225 pages containing these tables are provided as clime_tables.csv (see folder data/corpus in code.zip accessible via https://doi.org/10.5281/zenodo.10933231). In each row, the csv file lists the occurrence of an individual climate zone table, specifying the type and providing metadata for the edition containing this table.

### The atomization-recomposition model in detail

#### Digit recognition model

As a first step, our goal is to train a single-digit recognition model for which we provide optimization and architecture details in the following. We built a seven-layer convolutional neural network using the Equivariant Steerable Pyramids framework (*97*), starting with an initial four-layer equivariant convolutional block with filter sizes {3×3, 3×3, 5×5, 5×5} and eight-rotational groups invariant to translations and rotations on the ℝ^2^ plane. Low-level features required to detect digits (lines, arches, and circles) thus generalize over spatial input transformations resulting in increased data efficiency.

Subsequently, a pooling layer selects the map with the maximum activation from the equivariant group. A series of three standard convolution layers with kernel sizes 5×5, 1×1, and 1×1 outputs 10 activation maps $a_{j}{\left(x\right)}_{j=0}^{9}$ corresponding to the digits 0 through 9. Last, we capture variations in scan orientation and page size by determining the scaling factor and rotation that result in the maximum activation of single-digit activation maps.

We optimized the model using equal amounts of single-digit and nondigit patches, resulting in around 8000 data points for training. These data were further augmented with small rotations (±10^∘^), translations (0.025 × img_width/img_height in *x* and *y* direction), scaling (0.8−1.2×), and shearing (±5^∘^) transformations.

Since numbers can occur in various contexts beyond a table, e.g., as a page number, we model the local page context and consider a border of 10 pixels around the digit’s bounding box. We used the Adam optimizer to minimize the mean square error between true activation maps and model outputs using the loss term *ℓ* = *ℓ*_bbox_ + 0.3 ⋅ *ℓ*_context_ and selected the model that performed best on the test set.

#### Bigram expansion

In the subsequent recomposition step, we combined these single-digit activation maps to detect digit task-relevant bigram features using a hard-coded sequence of processing layers. We compute the composed feature representations by applying an element-wise “min” operation

$$a_{\mathrm{jk}}^{\left(\tau \right)}\left(x;s,\theta \right)=\text{min}\left\{a_{j}\left(x;s,\theta \right),\tau \left[a_{k}\left(x;s,\theta \right)\right]\right\}$$

which signals the presence of bigrams *jk* ∈ {00, …, 99} at image scale *s* and rotation θ and can be seen as a continuous “AND” (*98*) operation. We also included additional feature maps that detect isolated single digits *j* ∈ {_0_, …, _9_} with “_” indicating that no digit is detected at the given location and that the translation operation τ shifting activation maps by a fixed number of pixels δ in the horizontal direction. To account for variations in spacing between characters, we generate bigram maps with multiple shifts δ and select at each spatial location the best shift via the max-pooling operation

$$a_{\mathrm{jk}}\left(x;s,\theta \right)=\underset{\tau }{\text{max}}\left\{a_{jk}^{\left(\tau \right)}\left(x;s,\theta \right)\right\}$$

The “max” can be understood as a continuous “OR” operation, checking at each location whether a bigram has been detected in any of the candidate alignments. Furthermore, isolated single digits can be detected by computing neighborhood maps using shifts ±δ. These neighborhood maps are computed from the single digit maps shifted in left and right horizontal directions and the further computing of a binary map that signals the absence of digits. Then, a “min” operation over digit map **a**_*j*_ and both neighborhood maps will indicate the presence of isolated single digits. This results in a total of 110 feature maps.

In our experiments, we use a reference page height/width of 1200 pixels, *s* ∈ {0.5, 0.65, 0.8, 0.95, 1.0}, θ ∈ {−90, 0, 90}^∘^, and δ ∈ {8, 10} pixels. Last, we select bigram maps from the sets of scalings, rotations, and shifts for which the feature map activity is maximized.

#### Pooling

As a final step, we apply spatial pooling to implement invariance with respect to the table layout and to reduce dimensionality, giving us a bag-of-bigrams representation for each page. We experimented with different pooling strategies and found that a standard peak detection algorithm resulted in the best task performance while allowing for a directly interpretable decoding of numerical features.

For the activity peak detection of bigrams, we started from a set of 100 bigram maps **a**_*jk*_ with *jk* = {00, …, 99} which are added to 10 maps for isolated digits ${\hat{a}}_{i}$ with *i* = {_0_, …, _9_}, resulting in $\bar{a}=\left(a_{i},a_{\mathrm{jk}}\right)$. Since the max-pooling used for the bigrams reduces the overall activity levels in comparison to the isolated digit maps, we introduced a scaling parameter α to the latter $a_{i}={\hat{a}}_{i}/\alpha$.

Next, we subtracted a bias term calculated as the product of a relative scaling parameter β and the maximum pixel value across all maps ${\text{max}}_{\left(x,y\right)}{\bar{a}}_{\left(x,y\right)}$. The resulting maps were rectified to filter out weak background activity. For each of the 110 feature maps, we computed occurring peaks using the center of activity mass and further determine the linkage matrix using the distances between centers to perform a hierarchical clustering, assigning close-by activated pixels into groups of pixels that belong to one bigram. To limit the size of clustered regions, we define a maximum distance parameter *d* and select parameters using histogram Pearson correlation scores on the training patches and set α = 3, β = 0.12, and *d* = 15. The resulting center of mass coordinates lastly give the digit location together with the digit label.

#### Explaining similarity models

To better understand the features that drive the similarity predictions, we apply XAI (*47*–*50*), specifically the purposely designed BiLRP method (*76*). This method assumes a similarity model of the type *y* = 〈ϕ(*x*), ϕ(*x*′)〉 where ϕ is a neural network based feature extractor, and *y* measures the similarity between **x** and **x′**. The method explains the produced similarity score *y* in terms of contributions of feature pairs $\left(x_{i},x\prime_{i\prime }\right)$. Conceptually, the method computes these contributions by performing a backpropagation pass from the top layer to the input layer. Each step of the backpropagation redistributes contribution scores from a given layer to the layer below. The method stops once the input features are reached. In practice, the explanation is computed more efficiently by computing multiple standard LRP explanations (*75*) (one for each element of the dot-product) and then recombining them at the input via a matrix product. To compute each LRP pass, we apply the LRP-0 rule (*99*) and pool resulting explanations over pixel regions of 15 × 15.

### Evaluation

The evaluation of the different representations used in our approach using ground-truth data annotations is described in the following.

#### Single-digit accuracy

The trained digit encoder is used to predict digit maps on the held-out test set. For each patch, the resulting activation map is computed, multiplied by a bounding box region mask, and lastly sum-pooled, resulting in a vector of size 1 × 10. The maximally activating vector index gives the predicted digit used to compute the single-digit accuracy.

#### Full-page bigram histograms

We use the digit model to compute 110 single-digit and bigram activation maps from which we extract histogram summaries by applying peak detection or spatial sum-pooling. Ground-truth histograms are computed by identifying and counting all bigram and isolated single-digit occurrences. Each bigram count, *h_jk_* is optionally mapped to its square root to better handle the difference of scale between frequently occurring and rare digits and bigrams, respectively. Last, the Pearson correlation between ground-truth and computed histograms is computed for each page.

#### Cluster classification

To validate the resulting clusters, we use a subset of the full corpus that contains one- and two-page instances of the Sun-Zodiac tables. The corresponding 71 table pages containing more than 45,000 single digits are split into train-test (50/50) sets, and a nearest-neighbor distance model is fitted on the training set. For all remaining data points, we assign the class label according to different distance models and compute the cluster purity of the test split over 10 random seeds. We have compared different ways of extracting page representations: (i) Bigram: Bigram histogram counts were obtained using the bigram model with peak detection and square root mapping. (ii) Pooled: Activity maps were obtained as in (i), but instead of peak detection, we directly applied spatial sum-pooling to the bigram maps. (iii) Unigram: Instead of computing bigram maps, we built a 10-dimensional unigram count histogram using peak detection. (iv) VGG-16: We used the pretrained encoder of the deep image classification network VGG-16 (*96*) and extracted spatially pooled output feature maps after the last of five convolutional blocks.

#### Visualization

For visualization of the table representations, we performed a t-distributed Stochastic Neighbor Embedding (t-SNE) projection and use the same t-SNE projection coordinates throughout the paper. After ensuring that resulting projections are robust across a range of values, the perplexity parameter is set to 500. Depending on the analysis, we filter the rendered data points, for example, based on a specific time interval or geographical location as shown in Fig. 2. This two-dimensional visualization of the table representation is used primarily for illustration purposes. Given the limitations of such projections, we do not rely on it to infer any insights directly but instead use it as heuristic to guide data exploration by domain experts.

### Historical Corpus-level analyses

#### Temporal analysis

The first edition of the Sacrobosco Collection (1472–1650) with at least one page of tables was produced in 1494 and the last in 1647. During these 153 years, the publication rate varied considerably. We thus implement a sampling-based temporal analysis. At each time step *t_i_*, we determine sampling probabilities for each table page using a truncated normal distribution $N\left(t_{i},\sigma^{2}\right)$, assigning probabilities to data points falling within the interval (*t_i_* − σ, *t_i_* + σ) and setting probabilities for data points outside this range to zero. We sample *N* = 80 data points at each iteration, assign cluster membership labels to them, generate a cluster count histogram with dimensions of 1 × *k* with *k* representing the number of clusters, and subsequently compute the entropy for each histogram vector. Clusters are computed using *k*-means clustering (*100*) with *k* = 1500 clusters. We have further studied the robustness of our results to the choice of hyperparameter in the Supplementary Materials. The temporal evolution of entropy scores is computed for digit density thresholds of {0, 100, 200, 250, 300}, which refer to the maximum number of digits detected on a page and average entropy curves over 20 runs for each threshold.

#### Geographical analysis

To study the varying knowledge production expressed by the tables printed across 32 different printing centers, we compute entropy as the difference in entropy between the *k*-means cluster distributions and an uninformed uniformly distributed production process *H*(*p*) − *H*(*p*_max_), where *p_k_* represents the probability of assigning a table to cluster *k* with *k* = 1500. The term *H*(*p*_max_) = log (*N_c_*) with *N_c_* the number of tables printed in city *c* captures the maximum entropy that a cluster distribution for each print location can achieve. These differences in entropy scores are by definition below or equal to zero. They are minimized for cities that output low entropy distributions, i.e., by repeatedly printing the same material.

## Supplementary Materials

### The PDF file includes:

Supplementary Materials and Methods Supplementary Text Figs. S1 to S48 Tables S1 to S3 Legends for movies S1 to S5 References
