# Supplementary material for: Historical insights at scale: A corpus-wide machine learning analysis of early modern astronomic tables
Source: Sci Adv. 2024 Oct 23;10(43):eadj1719. doi: 10.1126/sciadv.adj1719 (PMC11498222; doi:10.1126/sciadv.adj1719)
Supplement: Supplementary file 1 — Supplementary Materials and Methods Supplementary Text Figs. S1 to S48 Tables S1 to S3 Legends for movies S1 to S5 References [file sciadv.adj1719_sm.pdf]

Science Advances
AAAS

Supplementary Materials for

Historical insights at scale: A corpus-wide machine learning analysis of early modern astronomic tables

Oliver Eberle *et al.*

Corresponding author: Matteo Valleriani, valleriani@mpiwg-berlin.mpg.de; Klaus-Robert Müller, klaus-robert.mueller@tu-berlin.de

Sci. Adv. **10**, eadj1719 (2024)
DOI: 10.1126/sciadv.adj1719

The PDF file includes:

Supplementary Materials and Methods
Supplementary Text
Figs. S1 to S48
Tables S1 to S3
Legends for movies S1 to S5
References

Other Supplementary Material for this manuscript includes the following:

Movies S1 to S5

A Materials and Methods

A.1 The Sacrobosco Collection from the *Sphaera* Corpus

The *Sphaera* corpus contains four collections. One of them is called "Sacrobosco," as all the collected editions are related to one specific text: the *Tractatus de sphaera* by Johannes de Sacrobosco (– 1256). This collection is composed of 359 different editions of printed textbooks used across European universities to teach the introductory class on geocentric cosmology and astronomy during the early modern period. These 359 editions were published between 1472, the year of the first print (and of the first-ever print of a scientific, mathematical text), and 1650, which marks the decline of geocentric astronomy almost a 100 years after the publication of Nikolaus Copernicus's *De revolutionibus orbium coelestium* in 1543, which introduced a mathematical system based on a heliocentric worldview to early modern academia. For each edition, only one copy has been collected and this copy is in turn considered to be representative of the entire print run. The Sacrobosco Collection comprises approximately 76,000 pages.

A.1.1 The Dimensions of the Historical Process

To understand the scale of the historical process clarified by the findings of this research, it is necessary to ascertain how many copies of the editions comprised in our corpus were actually circulating in Europe during the period under consideration. To do so it is crucial to estimate the size of the print runs for each edition. Unfortunately, this information is often completely unknown, and only circumstantial inferences can be made. These inferences draw upon a few direct sources related to the subject, knowledge about decision-making processes or practices of early modern printers, technical aspects of early modern book production, and, given that the sources analyzed in this research are university textbooks, familiarity with the distribution and circulation rules specific to the academic book market along with knowledge concerning the institutional relationship between universities and their local print shops (*58, 69, 105*).

Owen Gingerich addresses some of these questions and concludes that the average print run for textbooks in the mathematical disciplines during the early modern period was about 1000 copies (*63, 64*). More recent studies have not significantly altered this average estimate, although they have revealed significant differences in the sizes of individual print runs. For example, Isabelle Pantin has demonstrated that the size of the print run for astronomy textbooks used at the University of Paris during the second half of the 16th century was determined based on negotiations between university lecturers and local print shops (*106*). One parameter for making such decisions was either the estimate or else actual number of newly enrolled students, indicating to the printer the minimum size of the print run required.

Moreover, the same printer had to consider additional aspects of marketing strategies, such as the network of printers to which they belonged and through which copies of the same edition could be sold, including to students enrolled at nearby universities (*80, 107*). Due to specific aspects of book production, the investment rate per copy significantly decreased with the increase in the size of the print run, leading to print runs that tended to exceed what the market could absorb (*70, 108*). It is therefore reasonable to assume that, in the context of major universities such as those of Paris and Padua, or densely populated universities like the one in Wittenberg (*109, 110*), the print run could easily exceed 1000 copies. Conversely, an edition primarily intended for educating the youth at court, such as Pifferi's textbook, was likely never printed more than a hundred times (*111*) (*112, 71–114*).

Despite the considerable variability in the actual print runs for academic textbooks in the mathematical disciplines during the early modern period, it still seems reasonable to conclude, following Gingerich, that the average print run for these books was approximately 1000 copies. Therefore, the Sacrobosco Collection under examination here represents approximately 350,000 textbooks circulating during the 178-year period considered, utilized by students and lecturers across a geographic area extending from Krakow to Lisbon and from London to Rome (Figs.

S1,S2).

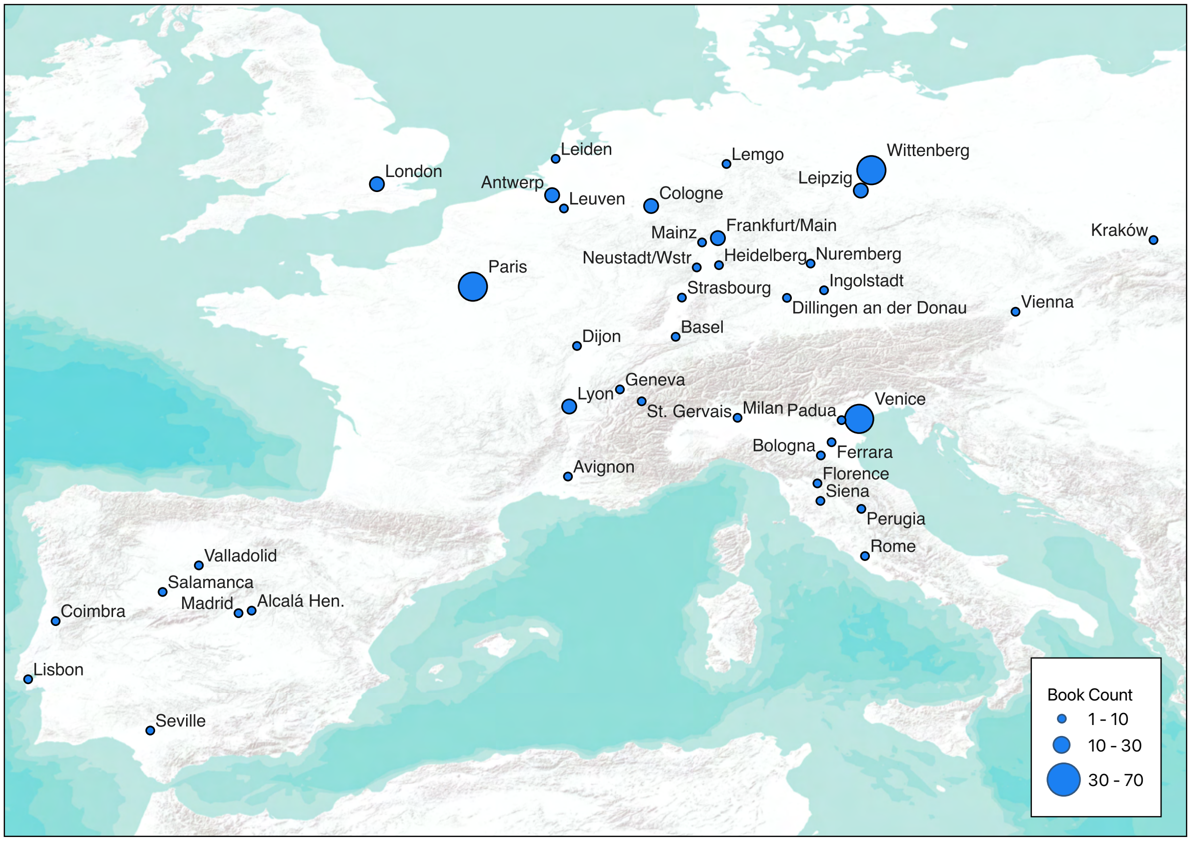

Figure S1: **The geography of the editions.** Geographical distribution of the production of the editions of the Sacrobosco Collection (1472–1650).

The treatise *Tractatus de sphaera* from which our corpus receives its name was originally compiled and published in Paris in the first half of the thirteenth century. As an elementary text on geocentric cosmology, the tract was used in astronomy classes at almost all European universities during the first year of the curriculum. Regardless of their ultimate field of study, these classes were mandatory for all students, as the discipline of astronomy belonged to the quadrivium. The quadrivium represented the curriculum of studies that any student had to accomplish during the first years at the universities in order to be allowed to gain access to further curricula, such as medicine, jurisprudence, or theology. Despite the relative simplicity of the

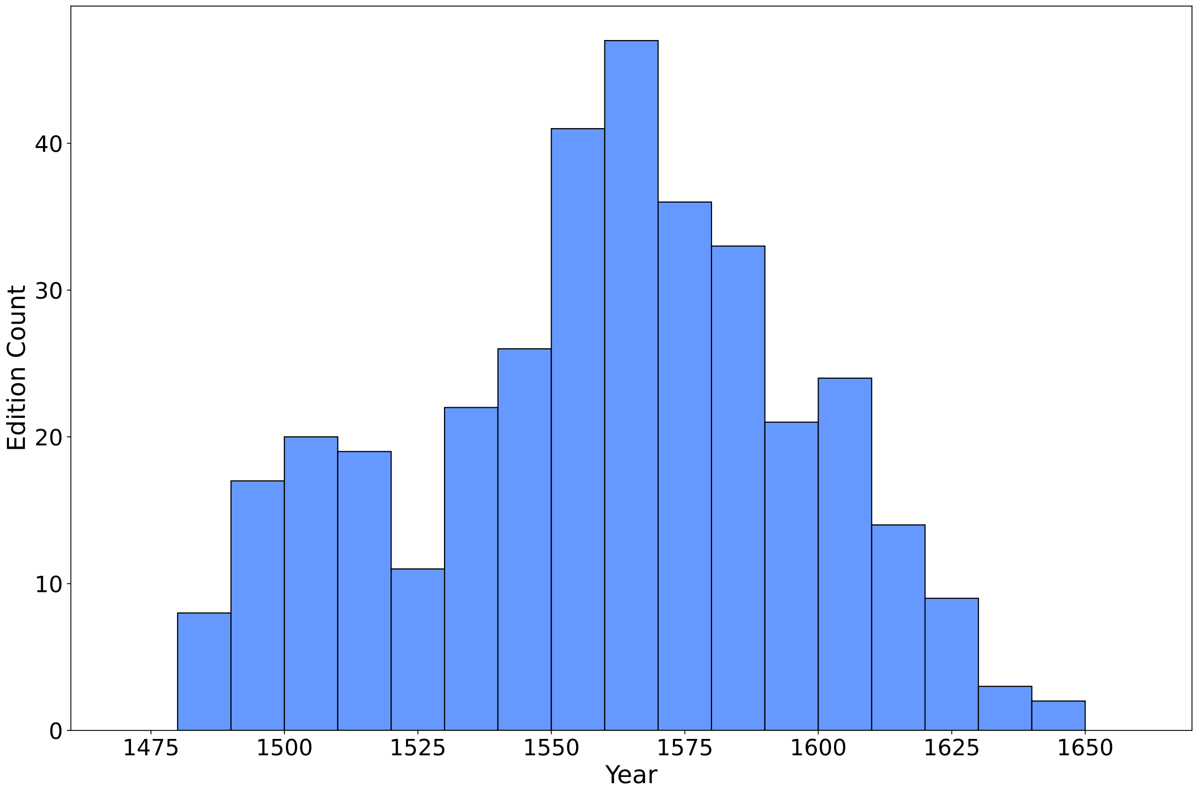

Figure S2: **The temporality of the editions.** Temporal distribution of the production of the editions of the Sacrobosco Collection (1472–1650)

.

treatise's content, its importance to understanding the transformation of knowledge stems from the fact that it was used from the thirteenth to the seventeenth century and was subject to continuous modifications by means of commentaries and further texts that were placed or printed together and which deepened attention to more specific, related subjects. This motivates to use this particular collection to investigate the broader mechanisms of knowledge transformation during this period. We rely solely on printed editions of textbooks that contain the *Tractatus de sphaera* in order to construct a structured and systematic dataset for the computational analyses discussed here, which cannot be generated by considering the older, late medieval and handwritten historical sources because of the lack of a census of them.

Focusing the research on university textbooks means that the work presented in our main article examines processes of scientific transformation on a large scale concerning the dominant knowledge of the educated society of early modern Europe. In other words, the corpus under examination reveals the "core knowledge" (*54*) possessed by those who became the readers of seminal works such as those of Copernicus and Galileo. It reveals their background knowledge and how this changed over time.

In general, we suggest a corpus analysis that follows three different axes, which can be re-aggregated at the end. The three axes emerge as based on three different types of data, into which we de-compose and dissect the historical sources. We call these different kinds of data "knowledge atoms." These are "text-parts," "visual elements" such as scientific diagrams and illustrations, initials, printers' devices, and "computational tables." The present work focuses on the investigation of the last of these knowledge atoms represented by numerical and alphanumerical tables, most of which result from calculations following astronomic computational workflows. In the case of our collection, the *Alfonsine tables* were the basis for many of these calculations (*65*).

Because of their complexity such computational tables could not hitherto be analyzed in great quantity either by humans or by machines. The page-based statistics for the collection shown in Fig. S3 highlights how book production varied over time and, more specifically, how table pages have increasingly been included as part of standard textbooks. In particular, it also shows that a) tables were almost nonexistent in university textbooks before the mid-sixteenth century, b) after their appearance, they quickly became a fundamental semantic element of these textbooks, as evidenced by the acceleration in their rate of publication. This acceleration is notably demonstrated by the fact that the increase in the absolute number of pages containing tables is not correlated with the total number of pages published, and even less with the total number of textbooks published, as displayed in Figure S2. As will be argued, focusing on the

computational astronomic tables is instrumental in investigating the process of mathematization of astronomy as taught at European universities during the early modern period.

The great variety of numerical and numerical computational tables in the collection considered here informs our modeling approach presented in the main paper and enables us to analyze and reconstruct scientific knowledge as disclosed and externalized by such tables. Before moving to this main subject, however, we briefly sketch the historical results already achieved on the basis of the other knowledge atoms while the data infrastructure needed to execute such research is described in the section A.7.1. This overview concerning the results of previous research is necessary to contextualize the results presented in this work and better understand their implications. Our dataset is retrieved from the research project "The Sphere. Knowledge System Evolution and the Shared Scientific Identity of Europe" (https://sphaera.mpiwg-berlin.mpg.de).

A.1.2 Studying Knowledge Systems

The dataset described in this section serves as the foundation of what we consider to be a knowledge system. Such a system results from the re-integration of the identified knowledge atoms into diachronic and synchronic graphs. We first describe the taxonomy used to categorize the 359 editions and then those graphs resulting from the analysis of the knowledge atom "text-part," which describes self-contained text sections in a book.

The rigorous historical analyses that form the foundation of the research resulted in the identification of five different edition classes within the collection, clearly differentiated by the form of their content in such a way to allow the identification of the modes of knowledge production in the period examined here (Figure S4).

The "original treatises" class represents a total of 17 editions, which exclusively contain the original text of the *Tractatus de sphaera* without added contemporary commentaries. The 48 editions, classified as "annotated original treatises," contain the original work of Johannes

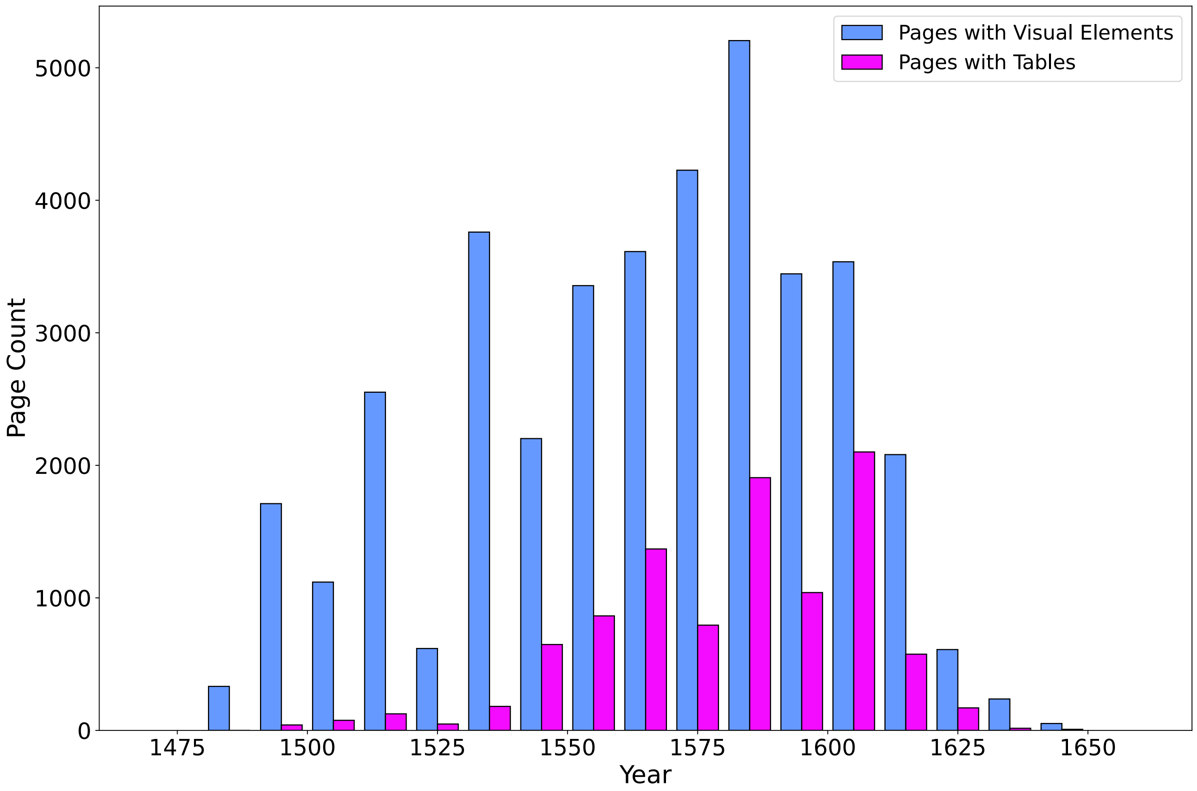

Figure S3: **Number of pages.** The histogram shows the variation of the number of pages containing visual elements and computational tables in the Sacrobosco Collection.

de Sacrobosco, with additional commentaries by various authors. As "Compilation of texts", we define a class of 43 editions, which include the original *Tractatus de sphaera* along with other original treatises by various authors, while the class "compilation of texts and annotated originals" contains 124 editions which include a commented or annotated *Tractatus de sphaera* along with other treatises. The final and largest class is constituted by editions defined as "adaptions", which numbers 127 and displays texts that are strongly influenced by the content and structure of the *Tractatus de sphaera*, but do not include the original treatise itself.

Each of these editions is dissected into text-parts. Each text-part represents a textual component that is both larger than a single paragraph and also conveys a coherent body of information. These text-parts are then classified into two main categories, 322 "content" and 261 "paratext"

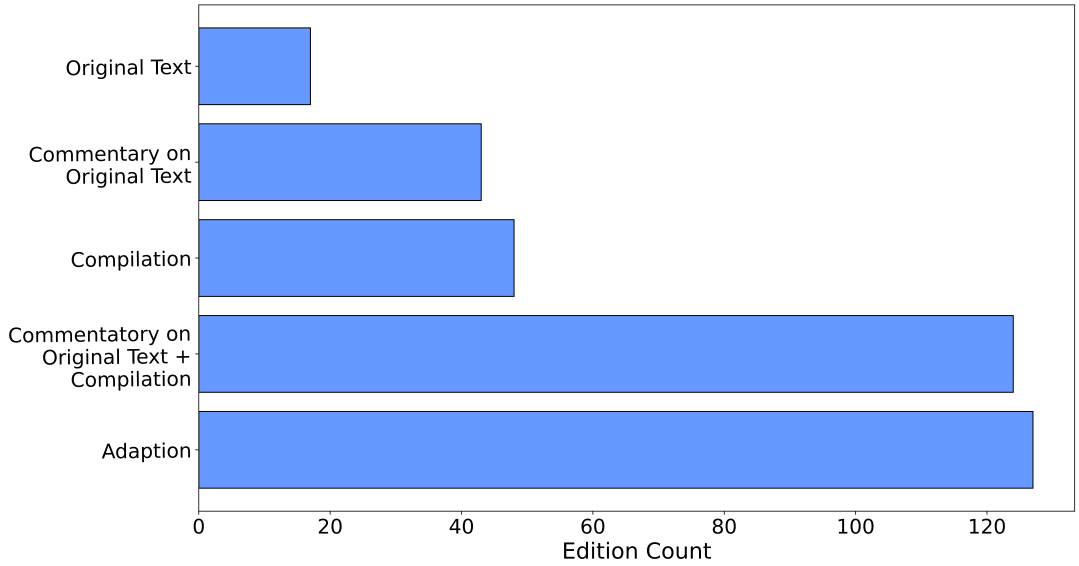

Figure S4: **Taxonomy for the editions constituting the Sacrobosco Collection.** Editions that contain the original medieval tract only; those that contain the original treatise with at least one commentary; those that contain the original treatise and other treatises (compilations); those that contain the original treatise, at least one commentary, and other texts; adaptions.

text-parts, the former referring to text-parts containing scientific treatises, while the latter refers to short texts that are often added to original content, containing poetry, letters to the reader, prefaces, dedication letters, or other literary compositions useful for the historian to understand the social, institutional, and political context in which the editions were conceived and produced (*113*). We built graphs (both diachronic and synchronic) among the editions on the basis of semantic relations among the text-parts that they contain.

To build a synchronic graph on the basis of the text-parts, we performed a content-related analysis in order to assess their mutual semantic relations: we related the text-parts to each other using the relationships “commentary of,” “translation of,” and “fragment of.” The diachronic graph is instead represented by the re-occurrences of text-parts over time. The integration of both graphs creates a high-dimensional matrix that, by adding the available historical metadata, allowed us to establish the multiplex networks by means of which we investigated the emer-

gence of epistemic communities within the corpus (*59, 60*); epistemic because the text-parts data represent the textual content of the textbooks.[1]

The dynamics of the 322 text-parts of the Sacrobosco Collection classified as "content" exhibit interesting non-linear behavior. Of these text-parts, 194 occurred only once, while the remaining text-parts re-occurred between 2 and 58 times. When these re-occurring text-parts are ordered chronologically according to their first date of appearance, two distinct phases with different re-occurrence dynamics can be discerned, separated by a transition phase (Figure S5).[2]

All re-occurring text-parts published before 1531 stop being re-published after 1545, while all the others first appear starting in 1531. The transition phase is precisely between 1531 and 1545 inclusive. If we consider only the text-parts that re-occurred at least once, we observe that: a) the percentage of text-parts that re-occur, calculated based on the total number of parts in each phase, is slightly higher in the second phase, b) the percentage of text-parts that re-occur in multiple cities, compared to those re-printed in the same location (local market), is also higher in the second phase, c) the ratio of the frequency of re-occurrence of text-parts in different locations to those that appeared in the same locations during the two phases changed significantly—from a ratio of 1.32 to 1.06 during the first phase to a ratio of 4.85 to 1.99 during the second. Considering the geographic distances, these figures indicate that, in addition to the distinction between the two phases, a sudden acceleration of re-occurrences took place after the transition phase.

As the text-parts denote the semantic content of the textbooks, the change in the dynamics of their re-occurrences can be interpreted as a change in the dynamics of the circulation of knowledge, specifically regarding the textual content of the historical sources at hand.

The first and most fundamental result of our previous network analyses concerns the process

[1]To interactively explore the dynamic of re-occurrence of the text-parts also according to their mutual semantic relationships, see https://sphaera.mpiwg-berlin.mpg.de/adoption.

[2]For readability, the plot displays only the text-parts that re-occur at least three times. For the complete dataset to replicate the analysis, see Section 3.

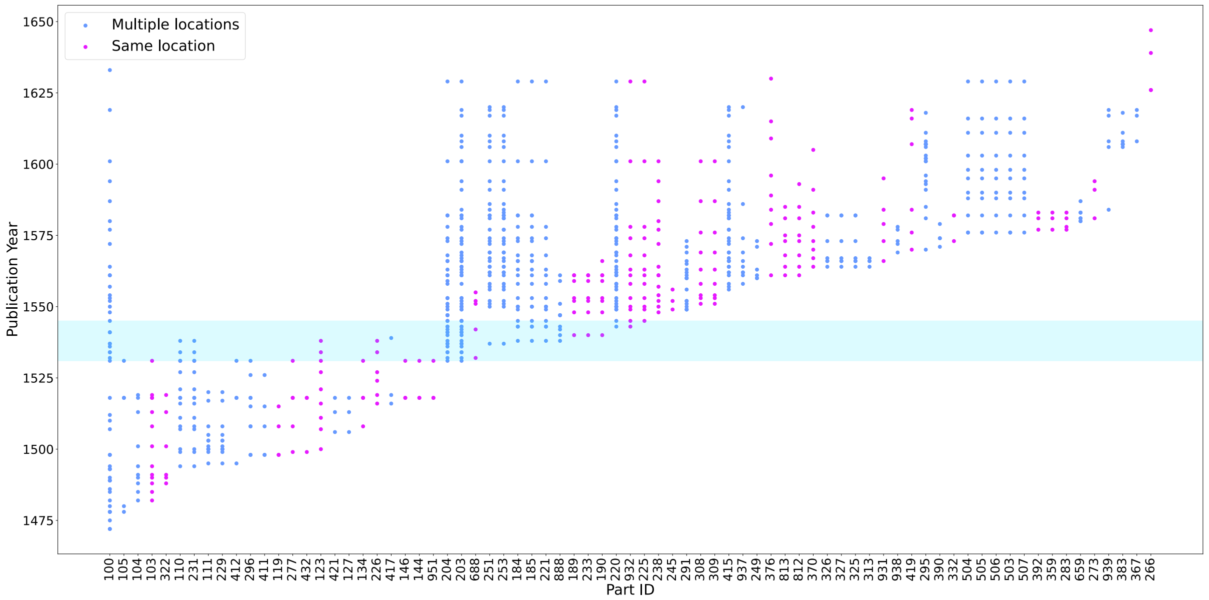

Figure S5: **Re-occurrence dynamics of content text-parts.** The dynamics show two phases separated by a transition phase (1531-1545): re-occurring text-parts published before 1531 are never published after 1545. The percentage of text-parts that re-occur, as well as the percentage of text-parts that re-occur in multiple locations rather than just one, is higher during the second phase. The ratio of the frequency of re-occurrence of text-parts in multiple locations compared to the frequency of re-occurrence in the same location changes significantly from the first to the second phase, shifting from a ratio of 1.32 to 1.06 during the first phase to a ratio of 4.85 to 1.99 during the second phase. These figures demonstrate an acceleration in the dynamics of text-part re-occurrences. As text-parts represent the semantic content of the textbooks, considering the geographic behavior of the re-occurrence dynamics, the plot illustrates an acceleration in the circulation of knowledge after 1531, likely prompted by the increased number of new text-parts introduced into the knowledge system. To simplify the visualization, we show only the text-parts that re-occur at least three times in the Sacrobosco Collection.

of knowledge homogenization and, specifically, the underlying mechanism, which we now can best describe as a mechanism of imitation (*58, 80, 114*). We were able to identify families of treatises characterized by their inherent text-parts similarity, which at the same time executed a strong influence—their content was imitated—on the content of other treatises produced elsewhere. By matching this analysis with the metadata, we were finally able to identify that the dominant family of treatises that gave birth to such a process was produced in reformed Wittenberg during the 1530s. Assessing all the reasons that brought the scientific production of Wittenberg into the sights of European scholars of the period remains a complex task and, as it will be shown, the present work represents a fundamental step forward in the understanding of this complex process, as shown in the main article. At this stage, however, it can already be stated that while the Protestant Reformation created a confessional, institutional, and political division in Europe, it also created the backdrop against which scientists made their first step toward the formation of a community that begins to show some of the traits characteristic of the modern international scientific society (see Section 3 of the main article). Other editions that could be identified and that we defined as "Enduring innovations" and "Great transmitters" show the relevance of Wittenberg, especially around the middle of the sixteenth century (*60*). At this point Wittenberg changed its strategy, moving from a more radically innovative position toward integrating innovations and tradition in a way that would have supported the primacy of Wittenberg's scientific literary output in Europe for many decades, thereby furnishing the fuel for a long-term process of homogenization. In conclusion, we were already able to show that, at the end of the sixteenth century, based on a mix of imitation and a center-emanating output of innovations, students across Europe were all learning the same astronomy and cosmology, at least concerning the scientific knowledge conveyed through the textual apparatus of the textbooks under investigation.

But the textual apparatus is not the only means used to convey knowledge in the textbooks.

During the early modern period, written text was considered highly authoritative. Science was produced mostly by commenting on older texts, be these medieval as in the case of the Sacrobosco Collection or from classical Greek or Roman antiquity. The texts of reference which were commented on usually were not changed or updated. They were, however, illustrated, and with regard to the visual apparatus the situation was different. Since the late middle ages, the use of visualization became increasingly prominent in Western science, a trend that continues to the present day. While medieval manuscripts of Sacrobosco's *De sphaera* rarely display more than five illustrations, early modern editions developed a visual apparatus that consisted of 40 to 50 illustrations (in certain extreme cases, even more than 70). While our research focused on the visual apparatus is ongoing (*29, 115–118*), traditional analyses seem to indicate that the leading role played by Wittenberg textbook production in the process of knowledge homogenization extended to the scientific visual apparatus (*82, 95*).

Finally, the third kind of knowledge atom, the numerical table, is the one our main article is pivoted around and, therefore, will be introduced in a separate section.

A.1.3 Numerical Tables and Their Role

The specific treatise around which the Sacrobosco Collection is centered (Sacrobosco's *De sphaera*) is a *qualitative* introduction to geocentric astronomy. Qualitative here means that students could learn the composition and the elements of the cosmos, in certain cases also by working with the corresponding mechanical device, the armillary sphere (Figure S6). Finally they apprehended fundamental notions concerning the movements of the celestial bodies: for instance that the outer sphere, the sphere of the fixed stars (firmament) moves from east to west on a daily basis and from west to east by about one degree every 70 years (precession of the equinoxes). What they could not learn by any means from this text was, for instance, how to calculate in advance the position of a celestial body such as a planet. This fundamental trea-

tise, which remained in use at nearly all European universities for about 400 years, was *not* an introduction to mathematical astronomy. During the thirteenth and fourteenth centuries, the period before the one considered here, only very few scholars had the chance and the skills to enter the realm of mathematical astronomy through the study of extremely difficult and rare works such as Ptolemy's *Almagest*. Outside this expert culture, astronomy was fundamentally non-mathematical; it was part of natural philosophy, which was essentially the result of a speculative search for causes of natural phenomena. Astronomy, like the other disciplines of the quadrivium (geometry, arithmetic, and music) was considered to be a mathematical discipline. But in the general cultural context of the Middle Ages, besides the fact that only a few scholars really possessed such mathematical knowledge, the mathematical apparatus of astronomy was considered only to be an instrument for calculations and not a method to describe the real world, only its appearance. Mathematical astronomy was not natural science.

The path toward modern astronomy and thus in part to modern science can be interpreted therefore also as a process of *mathematization*. Practical knowledge, for instance, such as the knowledge accumulated by specialized artisans and engineers in the frame of mechanics and machine building, was integrated into mathematics and gave rise to theoretical mechanics starting from the sixteenth century. For instance, from the integration of the practical knowledge of the artillerists and geometry, the new science of ballistics emerged during the sixteenth century (*120*). In the case of the so-called mathematical disciplines, the process of mathematization was realized following two different directions simultaneously (*121*).

On one side, the disciplines themselves evolved. Contrary to what is commonly believed, the above-mentioned studies have demonstrated that the geocentric worldview was not a stagnant scientific theory but rather a subject of lively debate. A myriad of observational data collected since antiquity still needed to find an appropriate theoretical framework. This dynamic led to the identification of specific sub-areas of study—for instance nautical astronomy—which in turn

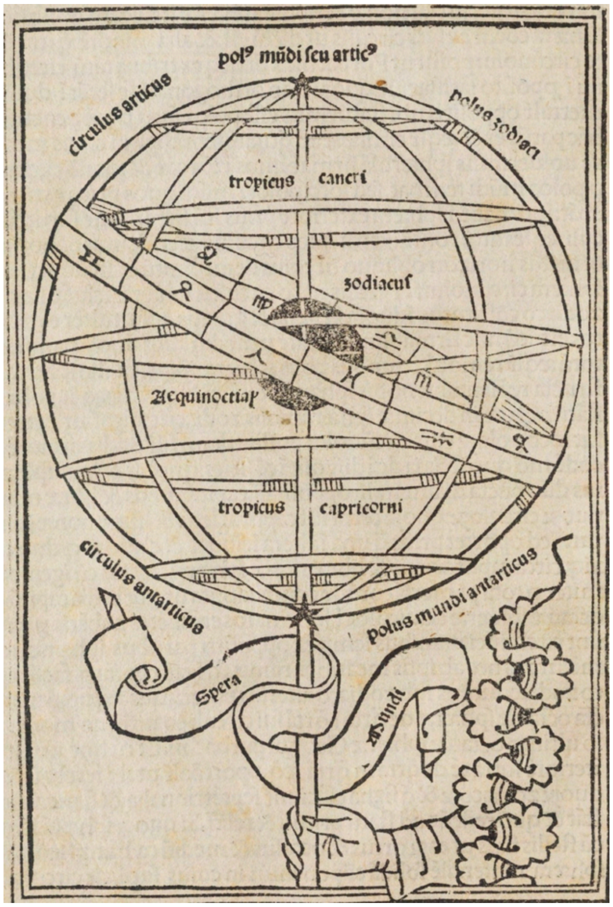

Figure S6: **Armillary sphere.** Typical graphic representation of an armillary sphere in a *De sphaera* textbook. An armillary sphere is a mechanical representation of the geocentric cosmos and, at the same time, a scientific instrument. From: (*119, sign. a-III-2*). Courtesy of the Library of the Max Planck Institute for the History of Science.

resulted in the creation of new textbooks. These texts were designed to be more accessible and focused on teaching not an all-encompassing mathematical system of the cosmos, but rather its individual aspects, such as the movements of each single planet or of only the outer sphere of the stars. These new texts—most famous among them those entitled *Theoricae planetarum*, from which students could learn a mathematical treatment of the orbits of each planet unconnected to the general view of the cosmos—actually were new text-parts added to the original tract of Sacrobosco. They lowered the threshold of access to mathematical knowledge in astronomy

and for centuries kept the traditional texts as relevant introductory material.

The lowered threshold complemented the second direction of the process of mathematization, one due to the emergence and increasing relevance of the universities, a genuine late medieval innovation in the framework of educational institutions. The late medieval and early modern universities linked disciplines that in previous centuries were not connected in such a systematic way. Particularly relevant for astronomy was, for instance, the increasing integration with medicine, which was to a good part the result of the reception of Islamicate science. Largely due to a revival of Galen's theory of critical days (*122*), astrological medicine became a fundamental scientific and cultural component of European society. As soon as sickness occurred, physicians were required to know the positions of the planets on the day of appearance of the sickness in order to be able to deliver a suitable prognosis. They were therefore very accomplished in using the *Theoricae* and its volvelles, paper instruments to determine positions of celestial bodies, to make precise calculations backwards in time.

Cultural trends like the one just described increased the demand for a mathematical approach to astronomy. The resulting process of astronomy's mathematization was a phenomenon characterized by an increased number of aspects of mathematical astronomy taught to an increased number of people. This process is inherently connected to the homogenization of scientific knowledge, as is clearly demonstrated by the comparison of, for instance, a pair of astronomy textbook editions, one from the fourteenth and one from the seventeenth century. What remains unclear, however, is exactly how such a process of mathematization worked, which kind of mathematics was really involved, what came first and how was it developed, whether all the attempts to introduce mathematical astronomy in a standard curriculum were successful, who promoted such process, and when and where it occurred. This process has never been reconstructed in detail concerning the history of astronomy—that is, leaving aside history of arithmetic and geometry—and the reason for this is that, until now, the historical sources that

can mainly disclose to us such a process could not be analyzed systematically. These sources consist of thousands of numerical tables, namely computational astronomic tables that were printed in the textbooks. In practice, we need to (1) identify recurring instances of particular tables across all printed editions, and (2) observe diachronic and synchronic trends in the inclusion of tables in the editions, averaged over the entire collection. Regarding the composition of the textbooks, it is relevant to mention that numerical tables are almost always connected to the textual apparatus. This means that if two similar tables are found in two different textbooks, it almost always implies that these textbooks also share the text-part to which the tables are connected. However, the reverse does not hold true. If a text-part that contains a table re-occurs, it does not necessarily imply that the re-occurrence of the text-part also contains a table.

Example of a Computational Astronomic Table

In Figure S7, we present an example of a computational astronomic table frequently encountered in the collection. This table of the "right ascension" gives the degree of the celestial equator measured from the vernal equinox eastward that rises together with each degree of the ecliptic in the "right sphere," i.e. for an observer at the equator of the earth (*66, 24–28*). Positions on the ecliptic are specified by degrees into the signs, with each sign listed in separate column of the table. Counting begins with the beginning of Aries. Thus for instance, 10 degrees into Taurus would correspond 40 degrees along the ecliptic from the beginning of Aries.

The relation between the equatorial latitude and the celestial latitude of a point on the ecliptic was derived by means of spherical geometry. The computational workflow in the table's background can be expressed in modern notation as:

$$\alpha = \arctan(\cos(\epsilon) * \sin(\lambda)/\cos(\lambda)),$$

where ϵ denotes the angle of the ecliptic, λ is the angle along the ecliptic and the right ascension,

i.e. the angle along the equator is given by α.

It is relevant to note that the vernal equinox coincided with this first point of Aries in antiquity. Hipparchus defined this point, also known as the Cusp of Aries, as the reference point for specifying celestial equatorial longitude (even though the vernal equinox entered Aries only approx. 100 years after Hipparchus' death). Due to the procession of the equinoxes the vernal equinox wanders about 1 degree along the ecliptic in 72 years. Thus in the sixteenth century the vernal equinox point would have been about halfway into Pisces and, strictly speaking, the tables in Figure S7 give the right ascension for an ancient observer in the first century BCE and are presented in Table S1.

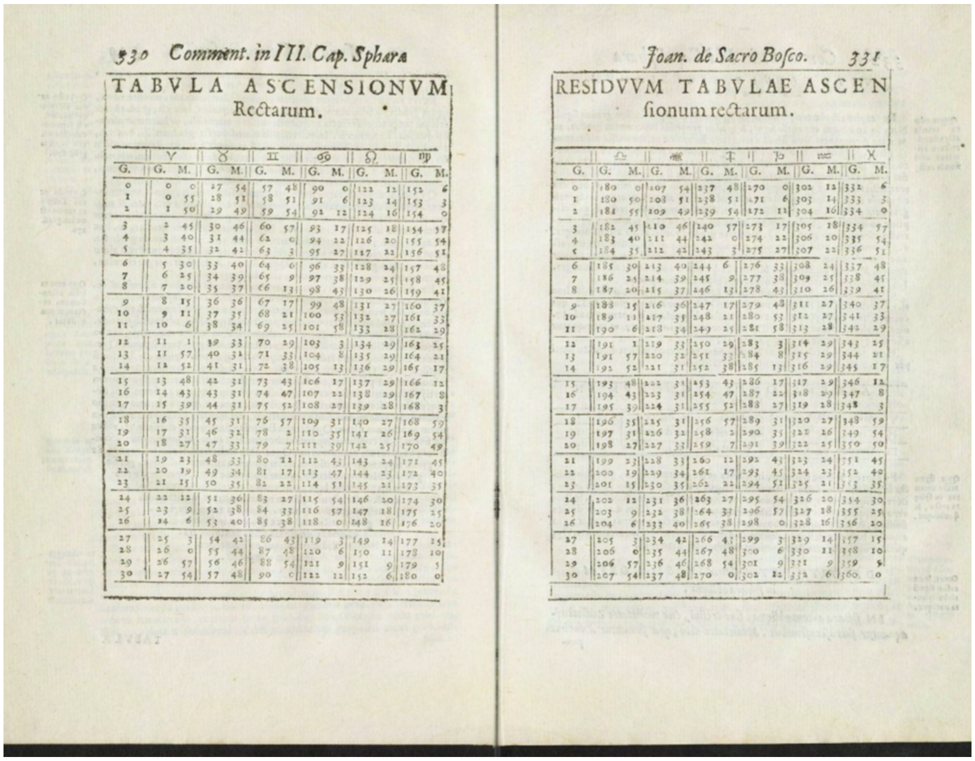

Figure S7: **Table of *right ascensions*.** Example of a computational astronomical table. From: (*123, p. 530*) published in 1585. Many exemplars of this table are contained in the collection. Courtesy of the Library of the Max Planck Institute for the History of Science.

	♈	♉	♊
1	0 55	28 51	58 51
2	1 50	29 49	59 54
3	2 45	30 47	60 57
4	3 40	31 44	61 60
5	4 35	32 42	63 3
6	5 30	33 40	64 6
7	6 25	34 39	65 10
8	7 21	35 37	66 13
9	8 16	36 36	67 17
10	9 11	37 35	68 21
11	10 6	38 34	69 25
12	11 2	39 33	70 29
13	11 57	40 32	71 34
14	12 53	41 32	72 38
15	13 48	42 31	73 43
16	14 44	43 31	74 47
17	15 40	44 31	75 52
18	16 36	45 32	76 57
19	17 31	46 32	78 2
20	18 27	47 33	79 7
21	19 24	48 33	80 12
22	20 20	49 34	81 17
23	21 16	50 35	82 22
24	22 13	51 37	83 28
25	23 9	52 38	84 33
26	24 6	53 40	85 38
27	25 3	54 42	86 44
28	25 60	55 44	87 49
29	26 57	56 46	88 55
30	27 54	57 48	90 -0

Table S1: **Rendition of the first three columns of the table of the right ascension, calculated according to the modern formula.** The angle used for the obliquity of the ecliptic is 23.5 degrees. There is an excellent correspondence to the values in the table given in Figure S7.

A.1.4 From Individual Tables to Corpus-Level Analysis: Assessing Similarity

Judging whether two tables are similar—in the sense that they express basically the same information—is a complicated and time consuming process which can only be accomplished by experts. As an example in Figure S8, we provide two different versions of a table of the

declination of the Sun with respect to the celestial equator. The relation expressed in this table is the angular distance of points on the ecliptic to the celestial equator. As can be read off the first row, the table, like in the case discussed in A.1.3 is completed under the assumption that the vernal equinox coincided with this first point of Aries based on comparable mathematical relation derived from spherical trigonometry.

While expressing the same astronomical relation, there are some substantial differences between the two tables in Figure S8 expressing this same relation. While the table on the right, taken from an edition of Oronce Finé covers merely one page, the table from which we show one page on the left, taken from an edition of Christophorus Clavius, stretches over nine pages in total. The reason for this is that Clavius lists the declination for corresponding points on the ecliptic for steps of 5 arc minutes along the celestial equator, while the step size in Finé's table is of one full degree. Thus only every 12th value in Clavius's table corresponds to a value in Finé's table, explaining why the Clavius's require more space. Somewhat anachronistically speaking, both tables list arguments and function values for the same function, but the step-size in which the argument progresses is much smaller in one than in the other.

This is, however, not the only difference between the tables. While Clavius specifies the declination in degrees and minutes, Finé, in addition to this, also adds arc seconds. Clavius thus for example gives a declination of 0 degrees 24 minutes for the point one degree into Aries, where Fine gives 0 degrees 23 minutes and 22 seconds. It is thereby somewhat surprising that Clavius, who obviously aims for higher precision using the smaller step-size, provides the more coarsely rounded results for the declinations. Moreover, Clavius's value is obviously not attained by rounding the value to be found in Finé, and we can infer that both values and thus in essence both tables resulted from separate, independent calculations.

This example has highlighted the analytical effort and level of expertise required to asses if and in which sense two tables are similar. It makes clear that such effort is indeed unattain-

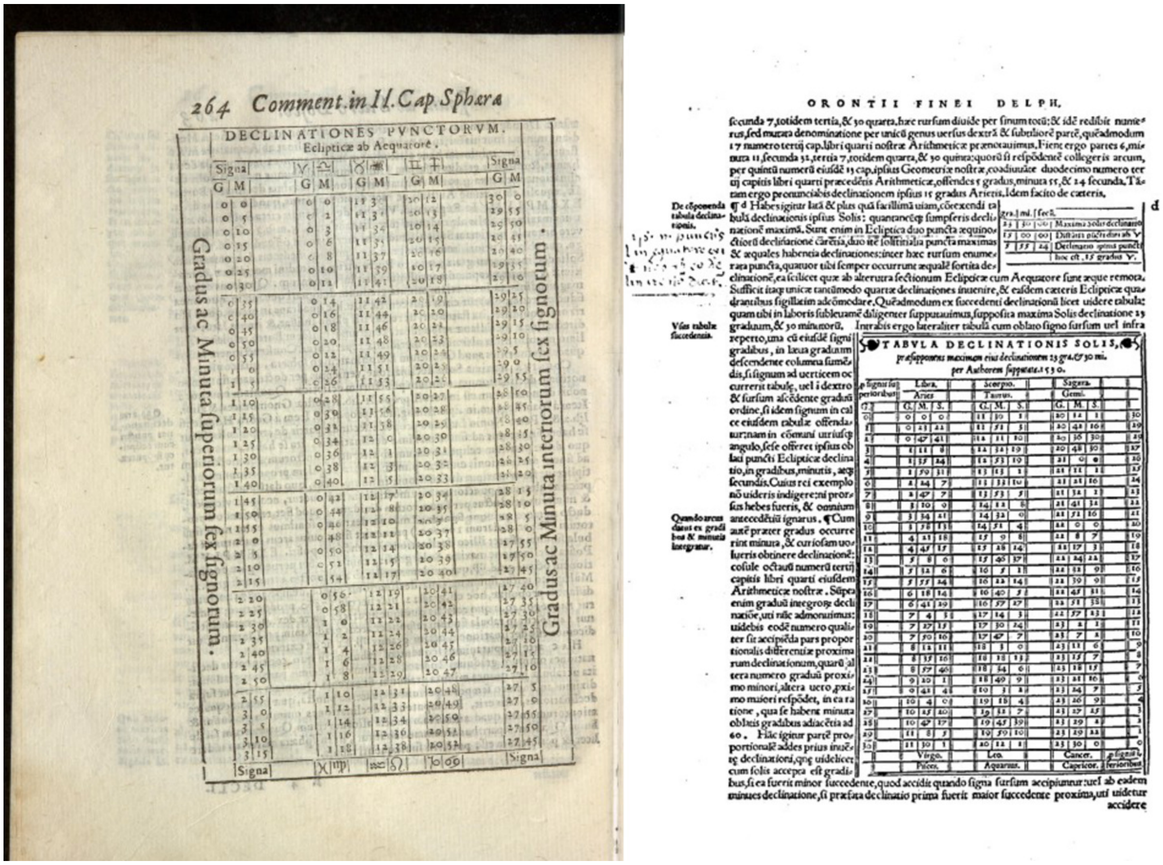

Figure S8: **Table of the *declination of the Sun*.** Left: (*124, p. 264*) published in 1591 (table continues over the next 8 pages). Right: (*125, Folio III-v*) published in 1532. Left: Courtesy of the Library of the Max Planck Institute for the History of Science. Right: Public Domain, Google-digitized.

able in a collection like ours with thousands of tables implying a myriad of comparisons. An expert would need to carefully inspect each of the individual digits composing the table. But even before this step, the tables would first have to be identified via a manual lookup of the $\approx 76,000$ pages of the Sacrobosco Collection. Now, the required analysis for the first time can be automated to a large extent or at least facilitated by the use of machine learning. By means of a page classifier described below (Section A.7.2 in Supplement Materials and Methods), first we were able to identify $\approx 10,000$ pages containing tables, which we also refer to as the Sacrobosco Table corpus. This implies that a manual assessment of table similarity would require a

meticulous examination of each table content from which similarity scores can subsequently be computed, or up to $10,000 \times 10,000$ manual pairwise table comparisons for an optimal result. This aspect ultimately clarifies why this material has remained inaccessible until now. However, this situation has changed due to the machine learning model we propose, as described below.

Building on the collection of automatically detected tables and using our model, we can now predict the similarity between every pair of tables, so that groups of similar tables (clusters) can be extracted or alternatively a list of most relevant tables can be retrieved from queries. However, for such machine learning approach to deliver accurate results (and to understand the reasons as to how we have developed the model), one needs to make sure that it applies reliably and systematically to the high heterogeneity of historical data, in particular the heterogeneity present in tabular data.

A.2 Data Heterogeneity

The challenge of heterogeneous data emerges across many domains and is one of the key limiting factors to automate data analysis processes. Heterogeneous data is characterized by a lack of uniform character and composition across instances in a dataset and more than 90% of big data can be called heterogeneous (*126*). Typical examples are unstructured collections of texts and images, i.e. from different online sources, biological, geographical or medical sensor data, as well as climate records. The field of information fusion offers methods that combine data from different sources in order to improve information content via integration.

In real-world applications data heterogeneity poses a serious challenge, even in fairly ideal scenarios in which sensors are comparable in function and measurement quality and standardized data acquisition protocols are in place. The heterogeneity of medical data, for example, is a key challenge to achieving robust models across hospitals and populations. Sources of heterogeneity can be divided into the following main categories: (i) technological heterogeneity,

due to different sensor manufacturer, recording protocols and data management; (ii) expert or institutional heterogeneity, caused by individual experts inferring different information from comparable material; (iii) underlying differences in the observed population and their environmental conditions. Each of these categories adds to the complexity of data and makes it difficult for ML models to generalize to unseen data and infer robust predictions (*127, 128*). Similar sources of heterogeneity are typical for historical corpora of materials which have emerged over the course of centuries and have been digitized only recently .

A.2.1 Heterogeneity in the Sacrobosco Table corpus

We illustrate some examples of heterogeneity in the Sacrobosco Table corpus using digit and non-digit patches in Figure S9 and Figure S10 respectively. We further analyse the various sources that produce the high heterogeneity of historical corpora and focus on the Sacrobosco Table corpus specifically:

> *Technological heterogeneity* is a result of both the historical printing process which has caused irregularities during typesetting as well as the more recent and non-standardized digitization process across libraries and research projects. Typical cases include: (1) the non-standardized digitization processes by archives and libraries over the last decades which has resulted in electronic copies that are extremely heterogeneous with regard to resolution, colors, size, and both production and post-production procedures. This heterogeneity is also attributable to different hard- and software set-ups. In addition, (2) the fragility of some historical material may not permit a standard digitization set-up, which extends to the fact that the section of the scanned page can vary greatly, as in Fig. S12 and that the page orientation is not standardized.

> *Institutional heterogeneity* concerns the question of what *similarity* between pages is

based upon, i.e. (3) whether layout and decorative elements are considered when judging table similarity (stylistic overlap) or whether similarity is based purely on semantic overlap.

Population differences reflect varying print traditions and printing quality as well as the preservation practice and status of the material. In the case of the Sacrobosco Collection, the original material treatises are (4) in very different states of preservation which is a result of their individual histories in the last 500 years. Moreover, (5) tables are printed in very different layouts, that is, the same table can "look" very different across books, as for example in Fig. S11; (6) depending on layout and format of the book, the same table can be found on one single page or stretched out over many successive pages in different books; (7) many of the tables are alpha-numerical, where the fractions of the "alpha" and the "numerical" components greatly vary; (8) each early modern printer had his/her own type-font and (9) numerical tables with many numbers were tedious to typeset resulting in a rather high level of noise of the actual with respect to the "correct" numbers. Finally, (10) pages can in part also be damaged, folded (Fig. S13), wrinkled, stained or de-saturated.

This high heterogeneity is further highlighted by the electronic copies of historical sources used in the entire Supplementary Material. We have consciously not post-processed these images, but left in the exact same way they can be found in the repositories of libraries and archives. As mentioned, such heterogeneity precludes using standard ML solutions and we will next describe different directions to deal with heterogeneous material before introducing our *atomization-recomposition* approach of analysis.

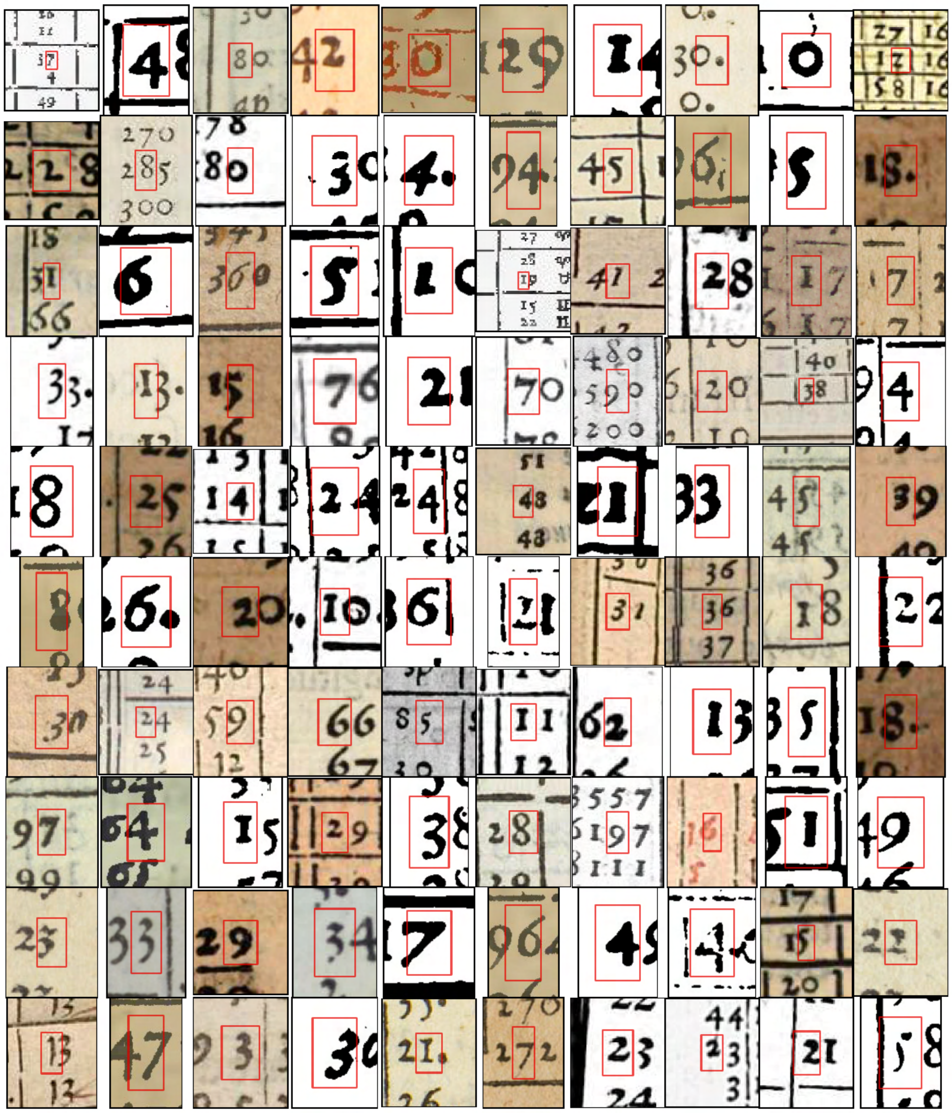

Figure S9: **Digit patches.** A hundred examples of the great heterogeneity in historical printing. The patches displayed are directly extracted from the scanned material before any pre-processing was applied. They are randomly selected digit patch examples used for the training of the digit recognition network.

A.2.2 Standard Approaches to Heterogeneous Data

Before model optimization, standardizing heterogeneous material through pre-processing is usually advantageous. This allows the ML model to focus on the extraction of task-related

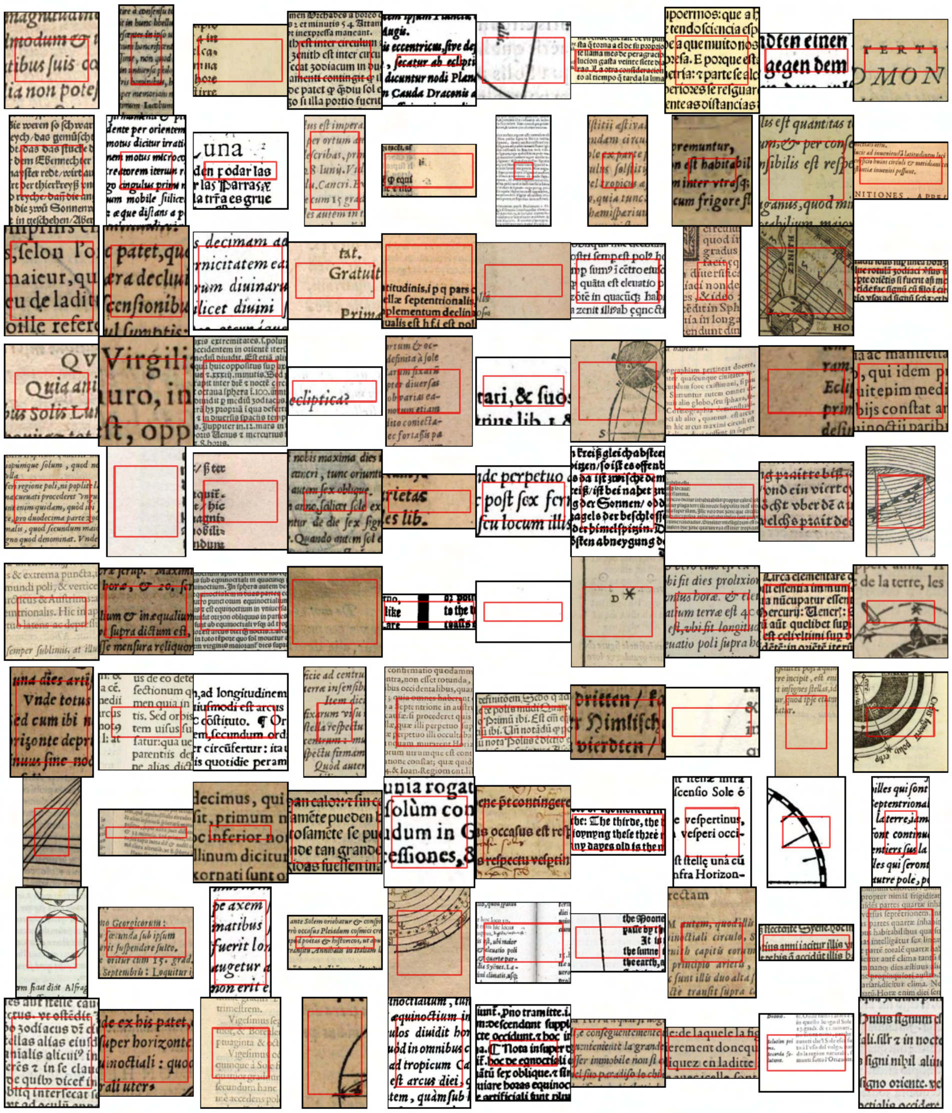

Figure S10: **Contrast patches.** Examples of non-table patches used as contrastive learning signal. Patches are extracted via randomly sampling regions from non-table book pages in the collection.

features rather than identifying and filtering various types of noise. Pre-processing includes standard centering of data using corpus statistics, thresholding and binarization of inputs, or transformation of input features, e.g. using whitening to de-correlate the data. This can be a powerful step to alleviate the heterogeneity that can be attributed to factors distinguishable

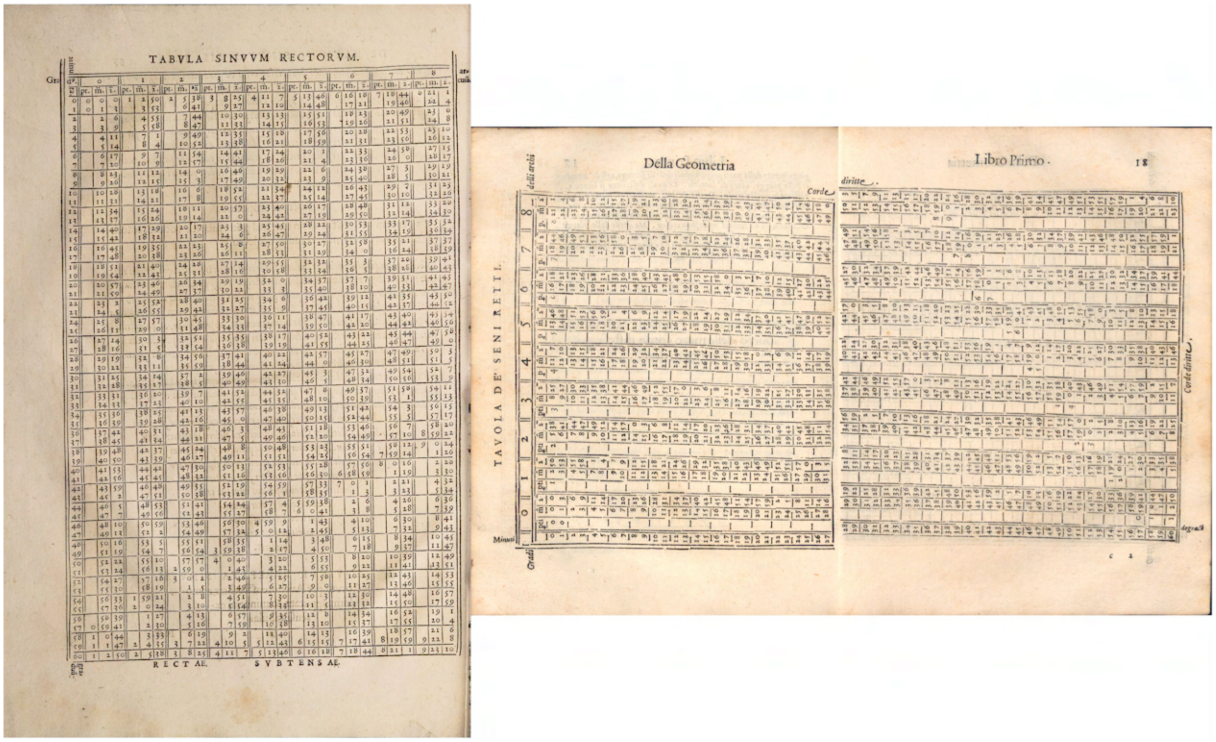

Figure S11: **Heterogeneity in layout of table content.** The same table of sinus values as published in two different works in 1542 and 1587. Typeface, layout, orientation, and number of pages on which the table is set are different. Left: (*129, p. 99v*), Right: (*130, Libro primo della Geometria, pp. 17v–18r*). Courtesy of the Library of the Max Planck Institute for the History of Science.

from the relevant signal via a statistical analysis of the raw input data, e.g. variations in color distributions across images, sensor noise, or varying signal strength.

Data heterogeneity that arises as a result of more complex variations usually has to be handled as part of an end-to-end training pipeline. This assumes that sufficient amounts of training data from sufficiently variable sources are available, and that these data can be used to extract representations that are invariant with regards to various types of heterogeneity. This being the case, one can attempt to infer structured information by transfer-learning from pre-trained models, but this requires that data distributions lie on the same or very similar data-manifolds as the training set. End-to-end deep-learning approaches especially have been a driver to bring

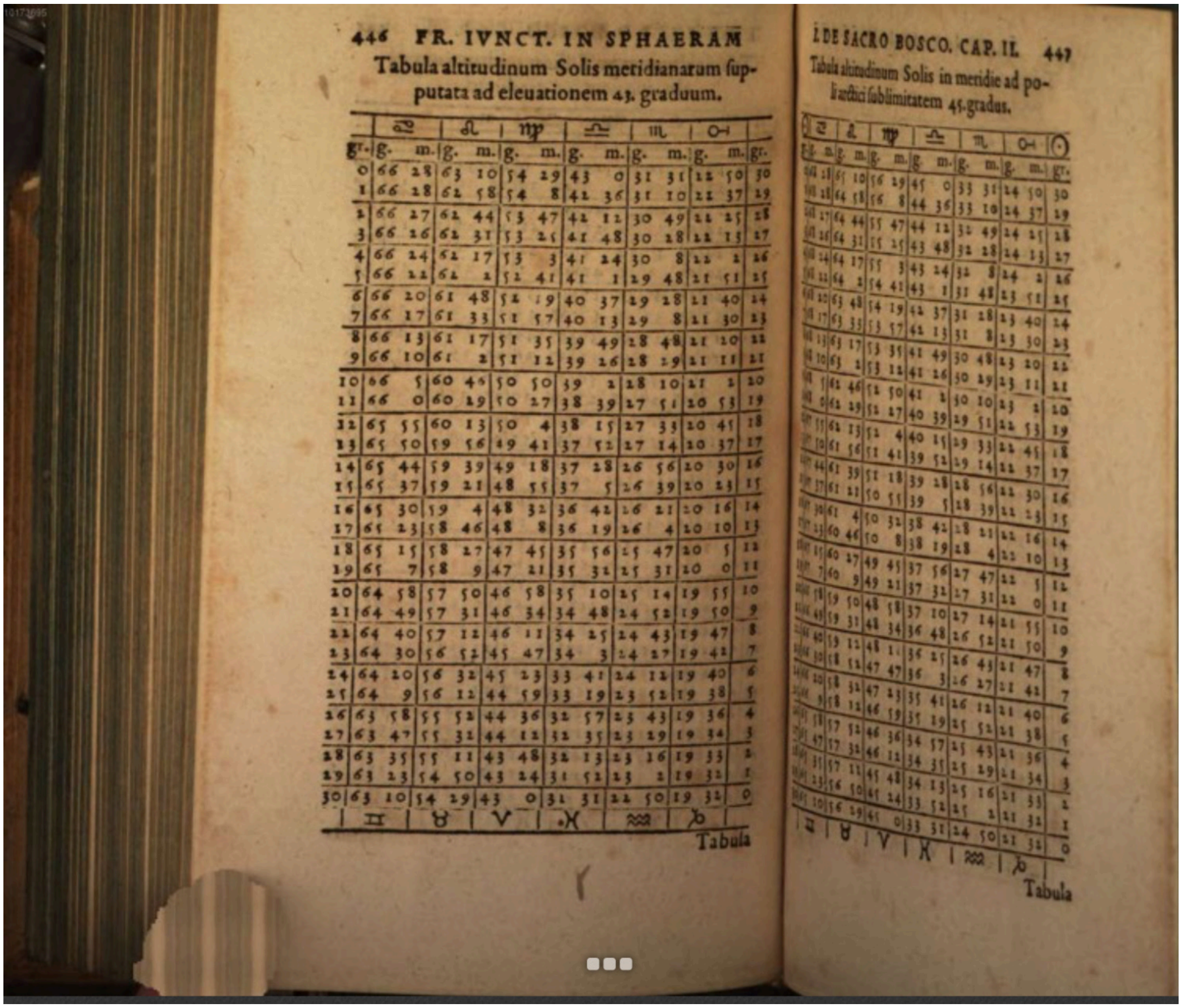

Figure S12: **Heterogeneity resulting from the acquisition process of electronic copies.** Due to the frequent impossibility of completely opening ancient rare books in order to avoid damaging the binding, electronic reproductions include the facing page captured with a different angle to the lens of the camera. In the bottom left corner, we can additionally see an example of how devices used to fixate the page during scanning are digitally covered during post-processing. From: (*131, pp. 446–447*). München, Bayerische Staatsbibliothek, Uniform Resource Name: urn:nbn:de:bvb:12-bsb10173695-4.

annotations to unstructured data. Prominent examples are segmentation models (*31, 133*) that are trained to extract object boundaries on images and have shown very promising ability for transfer to domain-similar material. These pre-trained and transferred models can serve as the

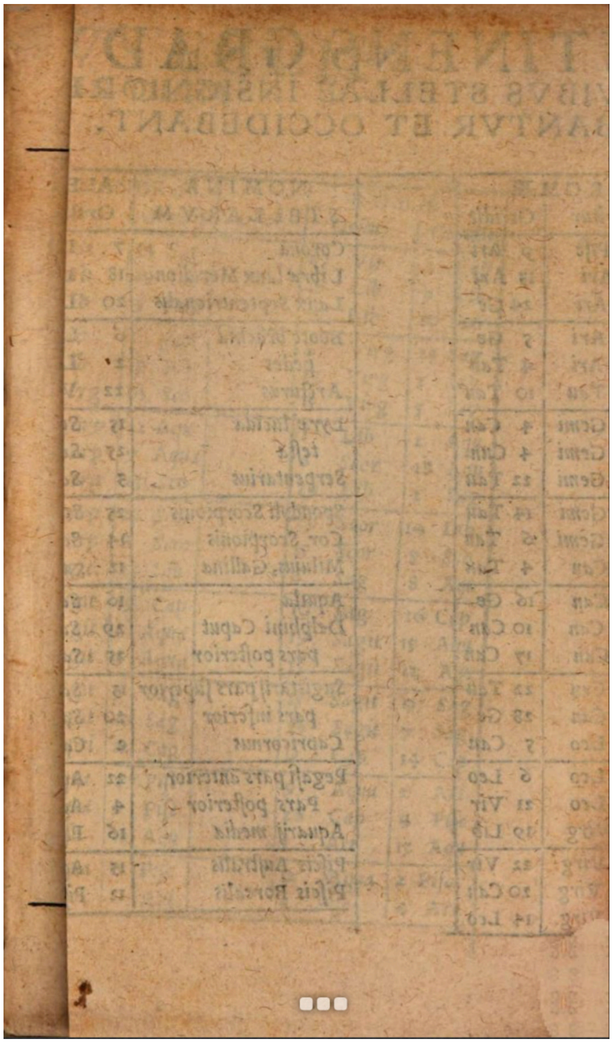

Figure S13: **Lost data due to folded table page.** The hitherto impossibility for historians to access tables on a large scale has meant that numerical tables and even computational astronomic tables have often been treated as historical sources of secondary relevance. This wrong assumption is confirmed by the practice of some archives and libraries to leave large-size numerical tables bound in the ancient books folded closed while scanning them. From: (*132, Unnumbered foldout*). München, Bayerische Staatsbibliothek, Uniform Resource Name: urn:nbn:de:bvb:12-bsb10998883-7.

basis for subsequent object classification and knowledge discovery in heterogeneous material. But again, the main limiting factor is the availability of either ground truth bounding boxes or object masks, which require human or even expert annotations. While community efforts

have resulted in the availability of such data in some domains, a transfer to novel applications remains extremely challenging, i.e. microscopy data in the biomedical sciences or historical material in the digital humanities.

Rather than collecting additional annotated data from various domains, the field of domain generalization aims to enhance the model's ability to handle semantically similar data from out-of-training distributions. This approach makes it possible to bring structure to unseen domains and improves invariance and robustness properties across data from different sources (*134–137*). Achieving these goals requires good knowledge of the data domain, as well as comprehensive labels that are similar enough to enable successful generalization. In our case concerned with table similarities, however, it is not possible to be provided with such labels in advance, which makes our development particularly innovative.

When dealing with historical material, we are limited to intermediate labels, e.g. character-level labels of digits. Nevertheless, we can leverage this data to build more complex features by employing our proposed atomization-recomposition approach.

A.3 The Atomization-Recomposition Approach to Represent Historical Material

In order to deal with the different types of variability in the Sacrobosco Collection, we will next give a detailed description of our modeling steps. Our proposed approach involves an initial atomization step, which entails breaking down the intricate composition of numerical features into their basic components. In our setting, this atomization refers to identifying single digits, the basic building block used to compose more complex numerical strings. Identifying single digits allows the model to handle heterogeneity at a much lower data complexity, as previously suggested in the remote-sensing literature (*138*). This further allows the use of simpler and in total less annotations, while still being able to handle challenges related

to robustness and invariance at a lower data complexity. The subsequent recomposition step provides the possibility to build in expert knowledge and design relevant features necessary to solve the final task. The **atomization-recomposition** approach can be summarized as follows:

Atomization

1. Determine the basic building blocks (atoms) in the input data.
2. Collect or extract atom annotations.
3. Train and validate the atom recognition model.

Recomposition

4. Recompose the atoms to build task-relevant features.
5. Verification of features using explainable AI.
6. Evaluate the model on the final task of interest for which annotations are attainable.

Detailed descriptions of the main steps of atomization and recomposition, along with the necessary validation and explainable AI-based verification are described below (Section A.5 in Supplement Materials and Methods). A visual demonstration on a pair of historical table pages is provided in Figure S17.

A.3.1 Pre-processing

As a first step, we apply binarization to the full corpus. This involves normalizing each image using min-max normalization, applying a percentile filter at 0.8 and using the 10% and 90% quantiles of the pixel value distribution as the high and low cutoff values, which produces the binarized image. This process addresses heterogeneity in color, difference in page background texture, as well as variations in image contrast and brightness. We define a reference page height of 1200 pixels to which all pages are scaled in proportion to their original dimensions using bilinear interpolation. This allows us to capture the statistics of the page features in sufficiently high resolution while still enabling the processing of full pages on standard GPU-hardware. We

used Tesla P100 and V100 GPUs with 16GB/32GB storage.

A.3.2 Atomization

The backbone of our approach lies in the robust recognition of the basic atoms. To achieve this, our model has to be able to detect the correct digit with high accuracy, while avoiding the production of activity with non-digit contexts such as text, symbols, or illustrations. We first introduce the recognition architecture built from the (i) *encoder* followed by the (ii) *convolutional_encoder*, forming our 7-layer neural network as shown in Figure S14. The encoder consists of a 4-layer block of equivariant convolution layers as proposed in the framework of Equivariant Steerable Pyramids (*101*). After all layers but the last, we use ReLU activation functions. The subsequent convolutional encoder processes extracted features of the first block further to build the digit detectors which output the single-digit activation maps. This block consists of three standard convolutional layers of kernel sizes $\{5\times5, 1\times1, 1\times1\}$, strides of 1×1 and padding of $\{2\times2, 0\times0, 0\times0\}$. For model implementation, we have used the `PyTorch 1.8.1` (*139*) framework.

Stylistic Invariance

To capture the significant differences in historic fonts appearing throughout the corpus, we have carefully designed the dataset to cover a representative set of fonts by sampling patches from different printers. The distribution of annotated digit patches over printers is shown in Figure S15. This results in a total of 2,494 annotated full number patches from which 4,687 single digit patches are extracted.

```
DigitModel(
  (encoder): Sequential(
    (0): R2Conv([8-Rotations], kernel_size=3, stride=1,
padding=1, bias=False)
    (1): ReLU(inplace=True)
    (2): R2Conv([8-Rotations], kernel_size=3, stride=1,
padding=1, bias=False)
    (3): ReLU(inplace=True])
    (4): R2Conv([8-Rotations], kernel_size=5, stride=1,
padding=2, bias=False)
    (5): ReLU(inplace=True)
    (6): R2Conv([8-Rotations], kernel_size=5, stride=1,
padding=2, bias=False)
    (7): GroupPooling([8-Rotations])
  )
  (convolutional_encoder): Sequential(
    (0): Conv2d(64, 64, kernel_size=(5, 5), stride=(1, 1),
padding=(2, 2), bias=False)
    (1): ReLU(inplace=True)
    (2): Conv2d(64, 32, kernel_size=(1, 1), stride=(1, 1),
bias=False)
    (3): ReLU(inplace=True)
    (4): Conv2d(32, 10, kernel_size=(1, 1), stride=(1, 1),
bias=False)
  )
)
```

Figure S14: **Atom recognition architecture.** An initial encoder block extracts invariant feature representations that are then combined into single digit representations in a second convolutional encoder block.

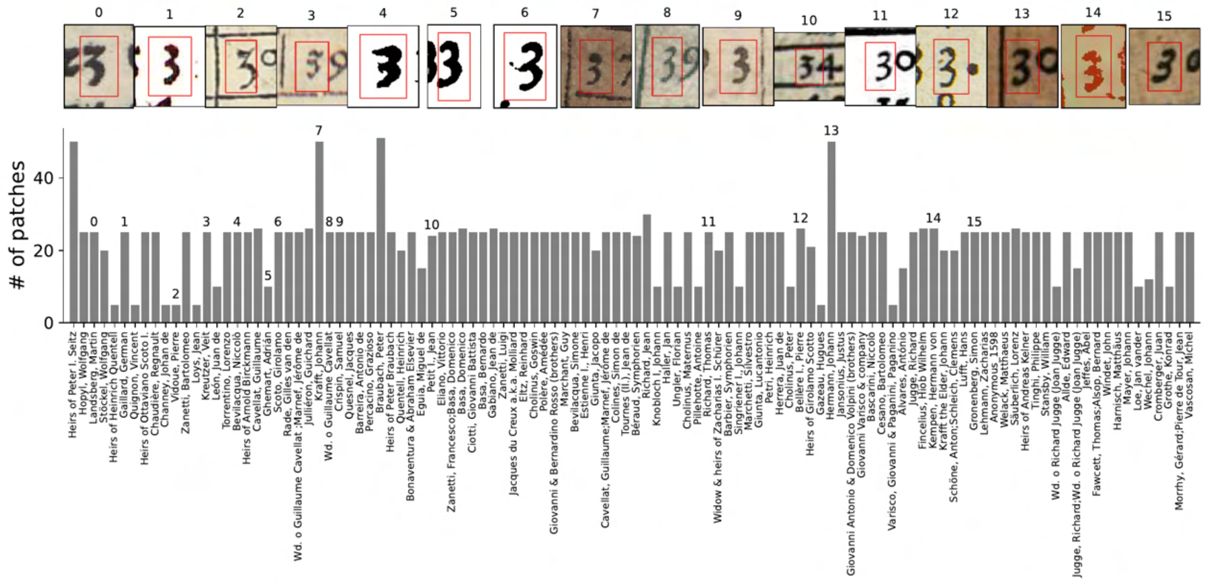

Figure S15: **Distribution of digit patches.** Histogram of number of annotated patches for each printer. For a randomly selected subset of printers, we show examples of the digit “3” they produced.

Local Scale and Rotation Invariance

To enhance the robustness of the learned representations against variations in print style and scale, we augment the training data patches through the following transformations: (i) Rotations of $\pm 10°$, (ii) translations by $(0.025 \times$ img_width/height) in both the x- and y-directions, (iii) proportional scaling by a factor of $(0.8 - 1.2\times)$ using bi-linear interpolation, and (iv) shearing transformations of $\pm 5°$ in both spatial directions. Each step of the augmentation process involves selecting a random value from the specified range for each augmentation, performing the patch transformation, and adding the resulting patch to the training dataset. In total, we sample as many augmented data points as there are annotated training patches.

Background Invariance Through Contrastive Learning

At a semantic level, each page can consist of a combination of many distinct elements including illustrations, text, mathematical equations, and tables. Each kind of element can be further divided into sub-categories, e.g. illustrations can be geometric diagrams, star maps, depictions of scenes, etc. and similarly tables can contain mostly text, mostly numerical values or—as is often the case—a combination of both. This poses an additional challenge during processing, since the recognition network has to be able not only to detect our desired features, but in parallel has to learn to ignore the entire non-digit content. Considering for example that the letter "O" is visually very similar to the digit "0," we aim to prevent page similarity to be based on such effects. To achieve this, we use all pages that do not contain any tabular structure in the Sacrobosco Collection and subsample pages from a diverse set of printers and books, similar to the selection of digit patches for annotation. A subset of these contrast patches is shown in Figure S10 and illustrates the diverse elements that can occur in the collection.

Training For model optimization, we use 80–20 train/test splits of the dataset and the digit model parameters are then trained using equal amounts of single-digit and non-table patches. We find that including context improves digit recognition, and thus we include a border of 10px surrounding the digit bounding box. We minimize the mean squared error between true activation maps and model outputs using the loss term $\ell = \ell_{bbox} + 0.3 \cdot \ell_{context}$ with the Adam optimizer.

The effect of training with or without contrast patches is further investigated in Fig. S16. For a random subset of fully annotated pages we show patches as processed by the single digit model trained on only digit patches (top row) and a model trained using equal amounts of digit and contrast patches (bottom row). It can be clearly observed that both approaches successfully attribute activity to the single-digits that occur in the various tables, and both achieve compa-

rable classification accuracies of 95–96%. However, naive training using digit patches only produces considerable activity over text, letters, and geometric elements as visible in S16.a (top row). We use the fully annotated subset of Sacrobosco pages to compute the ratio of all activity that falls inside the digit bounding boxes as compared to all page activity (S16.c) and find that without contrastive training, almost 60% of the activation occurs on non-digit locations, a number we can reduce to 9% when including contrast patches.

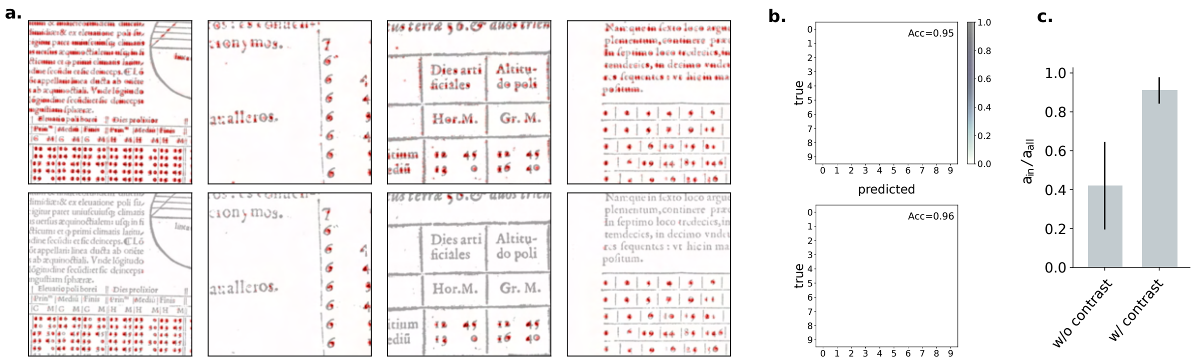

Figure S16: **Effect of contrast patches.** (**a**) Pooled single-digit activation of the model either after training on single-digit patches (top row) or after adding additional contrastive non-digit patches during training (bottom row). (**b**) Confusion matrices for the two different training scenarios. (**c**) Fraction of summed activation that falls inside annotated digit bounding boxes compared to the total page activation.

Global Scale Invariance

In the Sacrobosco Collection, we are faced with differences in global scale, which are a result of the different sizes of the movable type used during the printing process, i.e. larger or smaller typesetting, and also resolution differences occuring during the digitization process that can lead to several orders of pixel height and width spans in the data. We address these differences using a multi-scale feature pyramid similar to the framework of steerable pyramids (*140*). This approach offers the benefits of parameter efficiency, as it does not introduce additional trainable parameters, and preserves model transparency by relying on the linear decomposition

of images at various scales.

We implement this multi-scale approach by re-scaling the image to a reference height or width (portrait or landscape orientation) of 1200px at the reference scale $s = 1.0$ using bilinear interpolation. We obtain input images for each scale $s \in S = s_1, ..., 1.0, ..., s_K$ and pass each through the atom-recognition network. From the collection of resulting outputs, we determine the scale $s^* = \max_{s \in S} \sum_j \boldsymbol{a}_j(\boldsymbol{x}; s)$ that maximizes the spatially pooled activity over features j.

Global Rotation Invariance

Variations in page orientation likewise can result from either the printing process, where a landscape layout may have been chosen for better legibility of certain tables or illustrations, or the more recent digitization process. We address both of these factors in a similar way to how we address scale by including page input rotations of $\theta \in \Theta = \{-90, 0, 90\}^\circ$ and selecting the rotation that maximizes the activity response of our model: $\theta^* = \max_{\theta \in \Theta} \sum_j \boldsymbol{a}_j(\boldsymbol{x}; \theta)$.

With the different sources of heterogeneity addressed, we are now able to robustly extract single-digit activation maps. These representations will serve as the necessary building blocks to recompose more complex and task-relevant features, i.e. bigrams.

A.3.3 Recomposition

To efficiently achieve the recomposition of single-digit activation to bigram maps, we apply a hard-coded structure on top of the learned recognition model to compute bigram maps via an element-wise "min" operation:

$$\boldsymbol{a}_{jk}^{(\tau)}(\boldsymbol{x}; s, \theta) = \min \big\{ \boldsymbol{a}_j(\boldsymbol{x}; s, \theta), \tau(\boldsymbol{a}_k(\boldsymbol{x}; s, \theta)) \big\},$$

which signals the presence of bigrams $jk \in$ 00–99 at scale s and rotation θ, and can be seen as a continuous "AND" (*102*) operation. In addition, we build features that detect isolated

single digits $j \in \{\_0\_, \ldots, \_9\_\}$ with "_" indicating that no digit activity is present in the neighborhood. For this, the single digit activation maps and two binarized neighborhood maps with shifts $\pm\delta$ that signal absence of a digit feature are computed, and another "min" operation over all three maps outputs the final digit map.

The function τ denotes a translation operation that shifts activation maps by a specific number of pixels defined by δ. Multiple shift values of δ are used as candidate alignments and digit compositions are obtained by applying a spatial max-pooling layer:

$$\boldsymbol{a}_{jk}(\boldsymbol{x}) = \max_{\tau} \left\{ \boldsymbol{a}_{jk}^{(\tau)}(\boldsymbol{x}; s, \theta) \right\}.$$

Here, the "max" operator can be seen as a continuous "OR," assessing at each location whether a bigram has been detected across the different candidate alignments. This results in total number of 110 feature maps. In our experiments, we used $s \in \{0.5, 0.65, 0.8, 0.95, 1.0\}$, $\theta \in \{-90, 0, 90\}^{\circ}$ and $\delta \in \{8, 10\}$ pixels.

Activity Peak Detection

Having solved the challenge of identifying task-relevant features, next we would like to arrive at a summary representation of page content. To accomplish this, we can directly perform spatial pooling of activity over feature maps $\boldsymbol{a}_{jk}$.

While this represents a straightforward and effective method that yields meaningful similarities, as demonstrated in Section A.3.4, it may not provide clear insight into how the amount of pooled activity corresponds to the presence of features on a page. For example, a pooled activity score of 100 could arise from two highly prototypical bigrams that strongly activate the network—or from four less prototypical examples with weaker activation. In a first step, we apply thresholding before the spatial pooling operation since we are interested in the presence of features. Furthermore, we introduce a peak detection step to transform the raw activation

maps into bigram count maps.

We construct 100 bigram feature maps $\boldsymbol{a}_{jk}$ with $jk = \{00, \dots, 99\}$ to which we add 10 distinct maps that represent the presence of isolated single digits $\hat{\boldsymbol{a}}_i$ with $i = \{\_0\_, \dots, \_9\_\}$. In total, this results in 110 numerical features $\bar{\mathbf{a}} = (\boldsymbol{a}_i, \boldsymbol{a}_{jk})$. As a result of the the max-pooling operation used to identify bigrams, their feature maps show reduced activity levels in comparison to the isolated single digit maps. To harmonize activity levels, we introduce a scaling parameter α to the single digit maps: $\boldsymbol{a}_i = \hat{\boldsymbol{a}}_i/\alpha$, and further subtract a bias term computed as the product of relative scaling parameter β and the maximum pixel value across all maps: $\beta \cdot \max_{(x,y)} \bar{\mathbf{a}}_{(x,y)}$. We reduce weak activity of background features via ReLU rectificaion and further extract the feature regions that occur at non-zero locations to compute activity peaks using the center of activity mass. Using the distances between the identified centers, a linkage matrix is constructed, grouping close-by activated pixels into groups of pixels that belong to a single bigram. To avoid clustered regions larger than our features of interest, we define a maximum distance parameter d. Using Pearson correlation scores between ground truth annotated tables and our model-based bigram histograms on the training dataset, we select parameters $\alpha = 3$, $\beta = 0.12$ and $d = 15$. Using the center of mass as the digit location and its extracted feature label, we now have obtained a human-readable digit decoding as presented in Figure S17 (lower left overlay) that can serve as a useful verification and insight step during the historical analysis.

A.3.4 Demonstration of the Recomposition Steps on a Pair of Tables

In Figure S17, we present two example pages at different processing steps of the recomposition stage of our approach. The original page is displayed in full in the background and overlaid with the single digit activity. The inserts show bigram activity (top left) and the extracted digits (bottom left) after peak-detection was applied. Finally, we show the full histogram (bottom right).

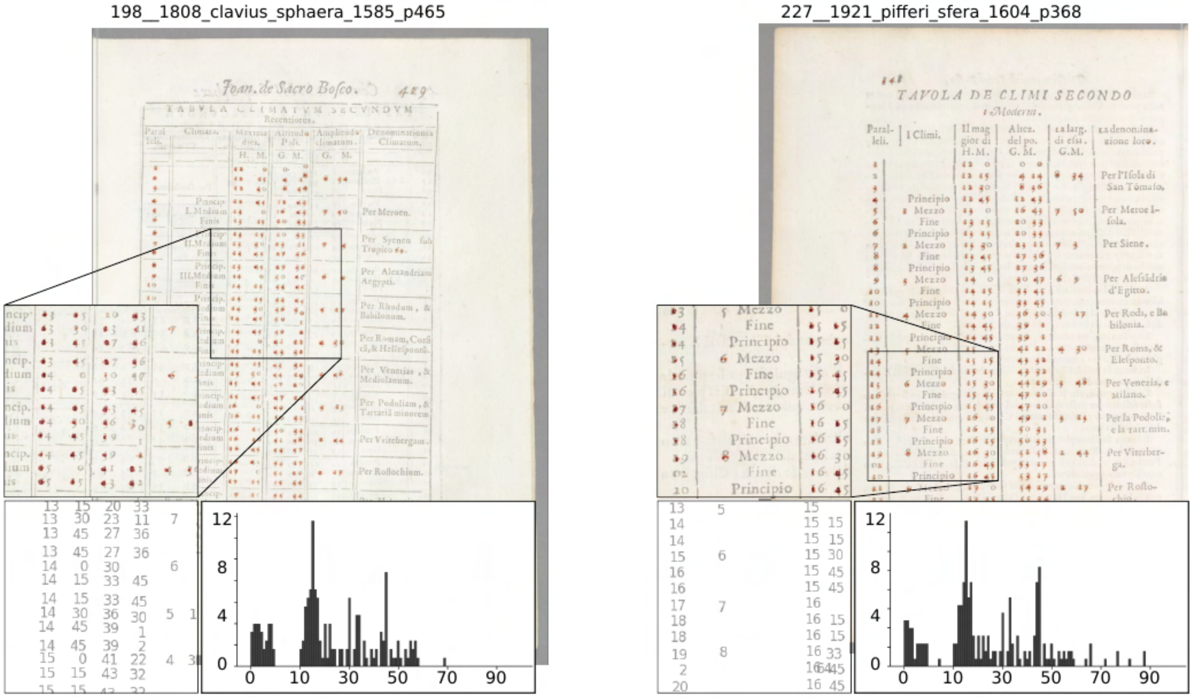

Figure S17: **Sequential processing steps to determine two table pages with the same content.** The background image shows the single digit activation maps pooled over digits 0–9. The zoomed in overlay contains the resulting bigram activations, below the extracted digits and the resulting histogram representation.

Comparing Activity Pooling with Peak Detection

We next want to quantify if, in addition to the above described advantages, the peak detection can also be used to provide a more accurate histogram representation. We have experimented with a non-linear mapping, i.e. the square root, of the histogram counts to take the scale differences between very frequent and rare number features into consideration. This allows to balance the vanishing contribution that less frequent occurrences have in presence of very frequent bigrams when computing distances or correlation scores. We use the fully annotated table pages and extract all occurrences of bigrams and isolated single-digits to compute ground-truth histograms for each page. The following approaches are used for comparison: (i) pooled bigram activity (Pooled), (ii) square root transformed pooled bigram (Pooled_sqrt), (iii) counts from the

peak detection processing (Bigrams), (iv) square root transformed peak detection histograms (Bigrams_sqrt), (v) square root transformed pooled unigram activity (Unigram_sqrt), and (vi) spatially-pooled VGG-16 output feature maps after the last of five convolutional blocks (VGG-16). In Figure S18, we see that peak detection based representations (Bigrams, Bigrams_sqrt) indeed increase Pearson correlation scores over the pooled activations. In addition, applying the square root transformation further improves correlation in both the pooled and peak detection scenarios. This can be explained by the increased sensitivity towards less frequent bigram counts.

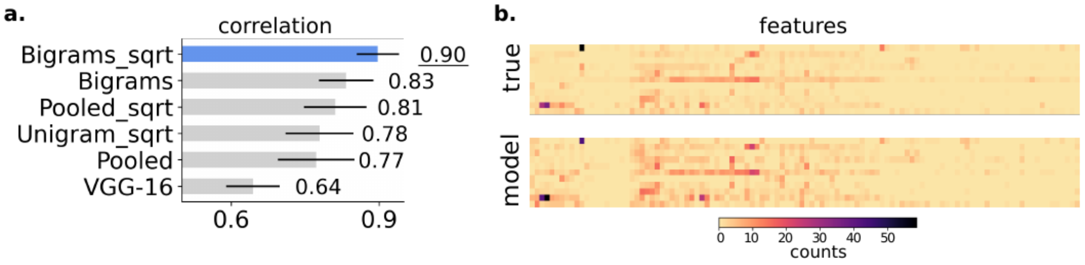

Figure S18: **Correlation on fully annotated pages. a.** Pearson correlation scores for different table representations. **b.** Ground truth and best model-based table histograms. In total there are 2261 bigram features in the source material.

Evaluating Cluster Classification Performance

In addition to the validation of the accurate detection of digit distributions in the previous section, we argue that the ML approach additionally has to be evaluated on the task we are ultimately interested in: in our case, the detection of groups of semantically similar tables. For this, we have used a subset of the Sacrobosco corpus that contains one and two-page instances of the sun-zodiac tables that are described in more detail in Section A.7.2 and have been annotated by a domain expert. The resulting 71 table pages contain more than 45,000 single digits, which are split into train-test (50/50) sets and the training set is used to fit a nearest-neighbor distance

model. For all test data points, a class label is assigned by the model according to the closest distance and, finally, test set cluster purity is computed for ten random seeds.

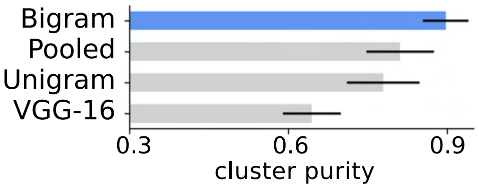

Figure S19: **Cluster purity of sun-zodiac tables.** Comparing the performance of different page representations in retrieving the correct cluster members as measured via cluster purity.

We have compared the following approaches to compute table page representations: (i) Bigrams_sqrt: Bigram histogram counts were obtained using the bigram model with peak detection and square root mapping. (ii) Pooled: Activity maps were obtained as in (i), but instead of peak detection, we directly applied spatial sum-pooling to the bigram maps. (iii) Unigram: Instead of computing bigram maps, we built a ten-dimensional unigram count histogram using peak detection. (iv) VGG-16: We used the pretrained encoder of the deep image classification network VGG-16 (*100*) and extracted spatially-pooled output feature maps after the last of five convolutional blocks. A visualization of the different page representations used to compute table embeddings is given in Figure S20.

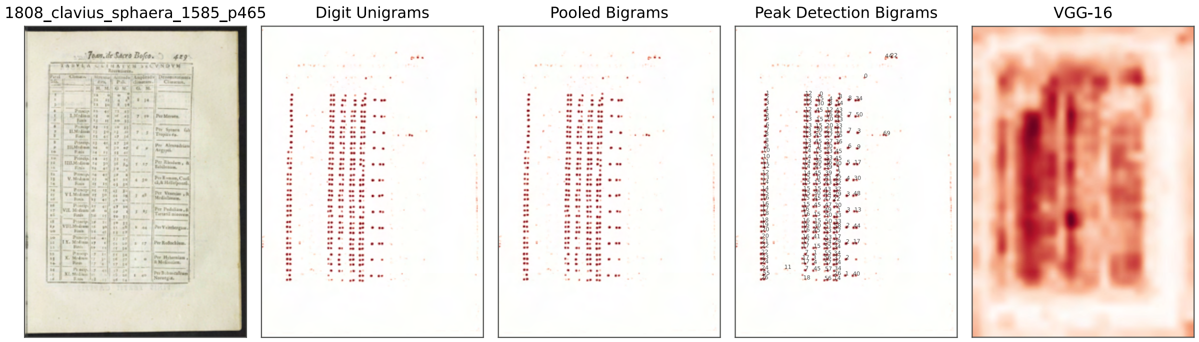

Figure S20: **Table page representations.** Different approaches used to compute table page representations shown as heatmaps.

Results in Figure S19 show that the bigram-based approach outperforms simpler unigram-

based or activity-based representations as well as VGG-16 representations with cluster purity at around 90%.

A.4 Limitations of Classical OCR Approaches for Digit Recognition

While traditional OCR approaches rely on simple computer vision algorithms to segment and extract characters from pages (*141*), the most recent approaches use more complex networks that segment the page and extract text regions, and a combination of convolutional and recurrent neural networks for character recognition and transcription (*142–144*). However, historical corpora, including the Sacrobosco Collection, present a major challenge for many OCR approaches due to their high degree of heterogeneity, characterized by diverse languages and fonts, complex page designs, and the myriad issues that arise from bad scans, faded text, bleed-through, smears, and damage incurred over time (*145*) (cf. Section A.2.1). While impressive progress recently has been achieved to bring standard OCR approaches to historical data, the representation of digits and specifically tables has not been addressed so far.

A.4.1 Evaluation on Fully Annotated Pages

In order to investigate the effectiveness of our approach with respect to traditional OCR methods, we compare our results with the output obtained from Latin OCR, a model build by The Duke Collaboratory for Classics Computing and trained on a large collection of Latin texts covering almost two millennia (*146*). Similar to our analysis in Section A.3.4, we use the fully annotated subset of the tables from the Sacrobosco Collection and compute Pearson correlation coefficients between ground truth histograms and the extracted model histograms. On average, we observe that our Peak Detection (PD) approach results in higher correlation scores that also vary less across different pages than the Latin OCR, which shows the effectiveness of our approach to detect digits (Table S2). In order to better understand the results, we report

the Pearson correlation on three different groups of pages (see Table S3), low number density pages ($\leq$ 150 bigrams/page), dense pages (150–300 bigrams/page), and very dense pages (>300 bigrams/page). The Pearson correlation scores in Table S3 clearly show that while our approach outperforms OCR in all of these classes, the margin grows with the numerical density on a page.

	mean	median	std
Latin OCR	0.747	0.849	0.272
PD	0.871	0.938	0.166

Table S2: **Pearson correlation.** The correlation is determined between ground truth annotations and our peak detection approach as compared to a state-of-the-art OCR system.

density	ρ_{OCR}	ρ_{PD}	$N_{\text{bigr.}}$	$N_{\text{uni.}}$
low ($\leq$ 150)	0.76	0.84	493	916
dense (150-300)	0.86	0.88	786	1501
very dense (>300)	0.49	0.93	982	1764

Table S3: **Effect of digit density on the Pearson correlation.** The correlation is determined at different digit density levels for a state-of-the-art OCR system and our peak detection approach.

A.5 Model Validation Using Explainable AI

Making modern and typically complex machine-learning models more robust to data distribution shifts and adversarial attacks is crucial for their application in science, society, and industry. The traditional ML evaluation pipeline seeks to validate the nominal accuracy of the model, but unfortunately, highly accurate ML models can ground their predictions in unexpected ways, i.e. via overconfidence in certain data features, reliance on spurious correlations, or classification sensitivity to noise. Thus, it is crucial to further validate the learned representations as well as the model's inner workings using additional techniques such as visualization and explainable AI (*147*).

Visualization and projection techniques, including clustering, are useful to analyze full datasets by representing them in a lower-dimensional space that can be directly interpreted by humans. Combined with labels, they can be used to measure how well a learned representation is able to separate datapoints from different classes, i.e. using cluster purity, normalized mutual information, or the Rand index. In the absence of any label information, formed clusters can be evaluated using distance scores as in the Silhouette Coefficient or Dunn's index. These unsupervised measures do not necessarily reflect user expectations since data points can be clustered perfectly, but built on unexpected or unwanted data features. Thus, manual validation of the projection or a subset thereof is crucial in moving towards a conclusive evaluation.

In order to evaluate the ML model itself and the features that are used for a certain prediction, the field of explainable AI (*46, 49, 148–150*) has developed techniques to make models transparent and reveal their inner logic. This transparency enables the development of more trustworthy systems which are of crucial importance when we are interested in generating novel domain insights. Historians, for example, need to be able to clearly understand which features in a document or collection of documents lead to a certain model prediction in order to arrive at well-grounded historical inferences (*53*).

A broad range of methods have been proposed for Explainable AI, and we briefly present here the "Layer-wise Relevance Propagation" (LRP) method (*75*) (see also e.g. (*46, 49, 149, 151*)), which applies to a broad range of complex classifiers, has advantageous computational and robustness properties, and an extension of which, called "BiLRP" has been developed to provide explanations for similarity models.

The LRP method considers a neural network composed of multiple layers, with input $\boldsymbol{x} \in \mathbb{R}^d$ and output $f(\boldsymbol{x}) \in \mathbb{R}$, e.g. the activation for a given class in the last layer. LRP seeks to attribute the prediction score to the input layer, specifically, producing scores R_i for each input feature x_i with $i = 1 \ldots d$. To achieve this, LRP operates layer-wise, starting in the top layer

and then redistributing the function output to the neurons one layer below. This redistribution proceeds layer after layer by means of propagation rules until the input layer is reached, at which point the explanation can be collected.

For illustration, let j and k be indices of neurons in two consecutive layers, a_j, a_k be the associated activations, and w_{jk} the weight connecting the two neurons. In the forward pass, activations between these two layers are typically related via the equation $a_k = \max(0, \sum_{0,j} a_j w_{jk})$. For such layers, LRP redistributes using propagation rules of the type:

$$R_j = \sum_k \frac{a_j(w_{jk} + \gamma w_{jk}^+)}{\sum_j a_j(w_{jk} + \gamma w_{jk}^+)} R_k,$$

i.e. neurons that are active and to which the model responds strongest receive more relevance than their counterparts. The parameter γ can be interpreted as a robustness parameter that needs to be tuned for explanation quality. When setting the parameter γ to 0, the procedure can be shown to reduce to simple methods such as Gradient $\times$ Input. Other redistribution rules can be used for different layers. We refer to (*103*) for further examples of propagation rules.

In order to bring verifiability to our approach, in particular, our similarity model of table pages is of the type $y = \langle \phi(\boldsymbol{x}), \phi(\boldsymbol{x}') \rangle$ where the $\boldsymbol{x}, \boldsymbol{x}' \in \mathbb{R}^d$ are two input examples, where $\phi : \mathbb{R}^d \to \mathbb{R}^h$ is a feature map (typically a neural network) and where $y \in \mathbb{R}$ is the predicted similarity score. For such models, the LRP approach is not directly applicable and one needs to consider its extension BiLRP (*76*). BiLRP recognizes that models with dot product outputs are intrinsically locally bilinear (instead of locally linear as for LRP) and thus better explained in terms of *joint* feature contributions.

BiLRP proceeds in a similar way as LRP, redistributing the relevance scores from layer to layer but this time using the propagation rule:

$$R_{jj'} = \sum_{kk'} \frac{a_j a_{j'} (w_{jk} + \gamma w_{jk}^+)(w_{j'k'} + \gamma w_{j'k'}^+)}{\sum_{jj'} a_j a_{j'} (w_{jk} + \gamma w_{jk}^+)(w_{j'k'} + \gamma w_{j'k'}^+)} R_{kk'}, \tag{1}$$

which bears resemblance to the standard LRP rule but includes many terms that are doubled. In this rule, j and k are neurons in two consecutive layers of the branch processing image $\boldsymbol{x}$, and where j' and k' are neurons in two consecutive layers of the branch processing image $\boldsymbol{x}'$. In other words, pairs of activations can only be relevant if they jointly activate and if the model responds to both of them. Like for the standard LRP, the parameter γ controls robustness of the explanation. If γ is set to zero, the explanation reduces to that of a simple second-order explanation called Hessian $\times$ Product (*76*). In practice, due to the quadratic growth of elements of the sum, the BiLRP procedure can be applied more efficiently by computing standard LRP passes for each of the individual elements of the dot product, and recombining the produced explanations using a matrix product. Resulting scores are then only combined at the input into the full relevance matrix.

The information in this matrix can be visualized by plotting the scores as connections between pixel locations i and i'. It can be beneficial to reduce pixel-level granularity of the explanation by grouping pixels into patches $(\mathcal{I}_1, \mathcal{I}_2, ...)$ and $(\mathcal{I}'_1, \mathcal{I}'_2, ...)$. We compare the explanations computed by our bigram network to a standard VGG-16 representation as shown in Figure S21. Explanations for the high similarity in the bigram network are indeed based on numerical content shared among the two images. Since we explain the dot product of histograms computed by spatial pooling over the page, we observe that feature interactions of the same digit can appear at different locations as visible for the bigram "12." While similarity between the VGG-16 embeddings is of comparable strength to the similarity score of the bigram representation, we find that it is predominantly based on task-irrelevant interactions, such as table borders and generally, geometric shapes, that interact across bigrams. In comparison to the bigram network, we observe that overall relevant interactions are less pronounced, which indicates the lack of a meaningful similarity structure that matches related items, and that some negatively relevant interactions contradict the similarity score. This highlights that model robustness and conformity

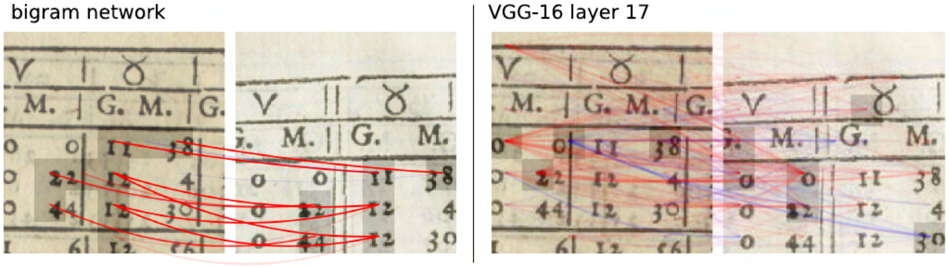

Figure S21: **Explaining similarity.** Left: Detailed BiLRP explanations highlighting the relevant feature interactions of predicted similarities between the two input tables for our bigram approach in red. Right: Resulting BiLRP explanations for the pretrained object recognition model VGG-16. Negatively relevant interactions are shown in blue.

with user expectations are not necessarily reflected by high model prediction scores, and that in order to produce reliable insights from ML models we need to verify their inner workings. We conclude that without having to collect ground truth expert-annotations of the table similarity, we are able to verify the proposed bigram approach from a single pair of tables.

A.6 Generating Historical Insights

The growing adoption of machine learning in diverse scientific domains has not only facilitated the extensive automated analysis and structuring of data but has also emerged as a valuable instrument for generating insights within specific domains, such as quantum chemistry (*152–155*), astronomy (*156, 157*), the climate and earth sciences (*158–160*), biomedicine (*161, 162*), and neuroscience (*163–166*). ML approaches have been especially fruitful in domains in which computer-aided experimentation and mathematical tools are already integral part to the research process. The automatic storage and processing of experimental data in those domains serves as valuable training data for ML models.

ML-assisted insight discovery has primarily found its application in the natural sciences (*50, 167*), but other fields have also begun to explore the potential for ML techniques to push existing boundaries of their respective domains. Examples include natural language processing

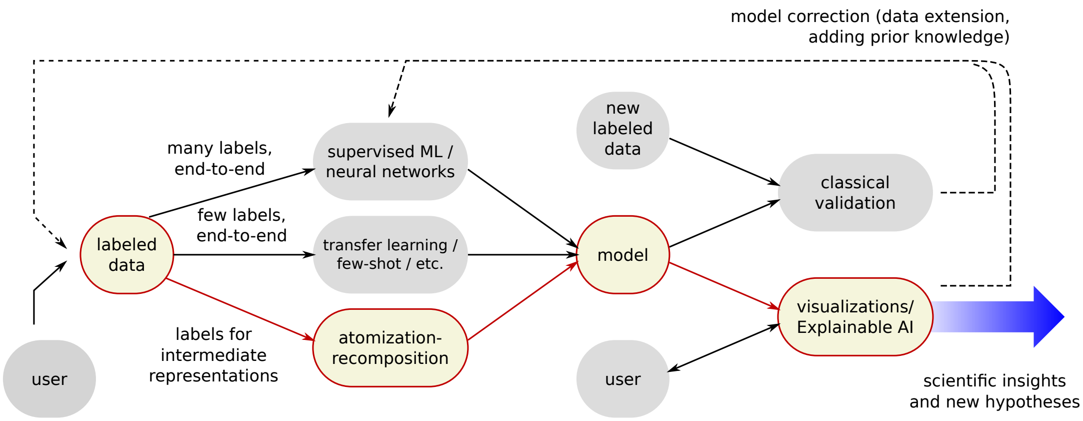

Figure S22: **Insights using Machine Learning.** Our proposed atomization-recomposition approach is embedded into the general machine learning and validation pipeline. The extraction of scientific insights relies on the analysis of interpretable model predictions in the form of visualizations and explanations by the user.

for under-represented languages, such as Sub-Saharan languages, or low-resource problems in the digital humanities and historical sciences (*168, 169*).

Machine learning in the humanities has been used for a broad set of tasks mirroring the diversity of disciplines ranging from archaeology, to history, literary studies, linguistics and philosophy. Most widely explored applications can be divided into the analysis of networks, images, and texts. Network studies construct a graph connecting items according to available metadata with the goal to explore and visualize large data, identify relational patterns, or execute an analysis of the community structure (*59, 60*). The analysis of image material takes advantage of advances in computer vision, and has been widely explored, for example, in the automated analysis of image style (*170, 171*), the extraction of similarity structures (*172, 173*), for the image-based classification of visual material (*29, 33, 174–177*), and for the extraction of images from historical documents (*29, 33*). Textual material has been analyzed in the context of topic modeling (*178–180*), ML-assisted annotation and text completion (*41, 181*) as well as modeling ancient languages (*182–184*). In addition, hybrid approaches, e.g., for the task of reconstructing ancient text from images (*41*), have been explored as well.

In Figure S22, we outline a general pipeline involving ML and XAI for extracting scientific insights from the data. ML models can be built in various ways depending on the practical constraints.

If sufficient training data and appropriate task-relevant labels are available or can be easily collected, a supervised model, typically a neural network, can be trained in an end-to-end fashion to optimize prediction accuracy (*185*). If few or no labels are available, one needs to leverage specific properties of the data and task. For example, if the task is related to another task with many labels, it can be tackled using transfer learning techniques (*186*). In a similar fashion, the relatedness of instances of a same class can be addressed via semi-supervised or few-shot learning techniques (*187*). Alternatively, in the absence of a suitable transfer task or specific forms of data relatedness, one can opt for the atomization-recomposition approach we have pursued in this work, where the labeling is performed at a more basic level (the level of "atoms," as referred to in our atomization-recomposition approach), and where the atoms constitute a starting point for recomposing a representation that solves the task.

All the approaches above produce a ML model. The model's predictive abilities can then be assessed using classical validation techniques, e.g. measuring prediction accuracy on a separate test dataset that was not utilized during the training phase. The model can also be further tested via XAI techniques, in order to verify that the high measured accuracy is not the consequence of a Clever Hans effect (*147*). Once the model is validated, the model predictive capabilities, possibly enhanced by visualizations or XAI, can be leveraged for generating insights.

A.7 Data Availability

A.7.1 Data Infrastructure

As mentioned, each of the 359 editions that form the Sacrobosco Collection is represented by a single digital copy that is considered to be a representative sample of the entire edition

print-run, resulting in a corpus that contains almost 76,000 pages. The result of this analysis is stored in a knowledge graph (*99*). The knowledge graph is modeled according to the Conceptual Reference Model of the International Committee for Documentation (CIDOC-CRM) (*188*), as well as its extension for bibliographic records, FRBRoo (*189*). The CIDOC-CRM ontology and its extensions provide a useful and standardized framework for modelling and storing humanities and cultural heritage data; the framework also strives to create coherent and shareable datasets across research institutions. This ontology relies on a predefined set of classes and properties, as well as constraints, to ensure the consistent recording and storing of cultural heritage and humanities data (*190*). Following the CIDOC-CRM standards, knowledge atoms were inserted into a knowledge graph, where entities (e.g., "books") are connected to each other through semantic relations validated by historians, effectively creating the *Sphaera* Knowledge Graph (*117*).

This knowledge graph forms the basis for all further investigations of the Sacrobosco Collection, and has expanded to become a number of times larger than its original size due to multiple consecutive historical and computational research cycles (*117, 191*).

The initial instance of the *Sphaera* knowledge graph stored metadata related to the physical version of the book, which included information that can be acquired by simply looking at each edition's digital copy. This information included the individuals involved in the edition's production (e.g. author, publisher, printer, and/or translator) as well as relevant information on the physical copy, such as the number of pages, physical format, and material, as well as the location of its printing and publishing. Further historical research gathered information on each person involved, such as their dates of birth and death, or alternatively their years of activity in cases when the former information is unknown, as well as any mutual kinship relations.

A.7.2 Data

In the following, we describe how we have obtained the Sacrobosco Tables corpus and provide details of the annotation process regarding ground truth used for training the digit recognition model as well as the evaluation of model and historical analyses.

Table pages

From the approximately 76,000 pages of the Sacrobosco Collection, we have selected 9793 pages bearing one or more numerical tables, which we submit to the table similarity workflow as the Sacrobosco Tables dataset. By numerical table, we refer to any tabular arrangement of data in our corpus which has at least one column with (predominantly) numerical content, and specifically exclude tables of content and book indices. This selection was supported by an of-the-shelf CNN (VGG-16 (*100*)) trained to classify numerical table pages. The output of this CNN was checked down to a low probability of the assignment of a page as bearing a numerical table. Due to the human post-processing, the list of pages with numerical tables has virtually perfect precision and very high recall. A list of all pages with numerical tables is provided as `sphaera_tables_9793.csv` (see folder `data/corpus`in `code.zip`) and the trained model instrumental in establishing this list is provided as `sphaera_tables_classifier.h5` in folder `data/trained_model` via https://doi.org/10.5281/zenodo.10933231. The digital images of the pages that we refer to as the Sphaera Tables dataset can be obtained at `sacrobosco_tables.zip` via https://doi.org/10.5281/zenodo.10933231.

Preparation and Acquisition of Ground Truth

Four different ground-truth datasets have been prepared to train and test our model, *single digits* and *non-digit content* to train the digit model, *fully annotated numbers* to test the digit recognition and the bigram expansion, and *sun zodiac pages* to evaluate the table similarity model. These sets are provided as `numerical_patches.csv`, `contrast_patches.csv`, `digit_page_annotations.csv` and `sun_zodiac.csv` in the code and data repository (see folder `data/training_data` in `code.zip` accessible via https://doi.org/10.5281/zenodo.10933231).

Single-digits In the period covered by our corpus, print types were far less standardized than they are today. To capture the wide range of typological variations present in our corpus, we have selected each printer that contributed at least one book to the collection. From the printed output for each of these printers, we have selected (where possible) five pages bearing numbers, and annotated on each of these pages five individual numbers by bounding boxes, annotating in addition the writing direction (upright, turned left, turned right), resulting in a total of 2494 annotated numbers. Single digit patches were obtained by dividing the annotation boxes into equal segments corresponding to the number of individual digits in the annotated number. As the types for the individual digits from zero to nine vary in width, this introduces some error in the single digit patches that becomes larger the greater the number of digits there are in the annotated number. After annotating about a third of the selected pages, we thus decided to restrict the annotation on the remaining pages to digit bigrams (adjacent digits, regardless of whether they form a two digit number or are a part of a longer number) but retained the annotations produced before. In total, the single digit dataset contains 5208 samples.

Non-digit content In order to correctly model non-digit page content such as text, illustrations, or layout geometry, we extracted patches from non-table pages as contrastive examples.

Fully annotated numbers We have selected 11 pages and annotated each single digit contained on the pages by a bounding box. In addition, we have marked if the individual digit is the first and/or the last digit of a number. With this information, all numbers and thus also all bigrams contained on these pages can be reconstructed. The annotated pages have been selected to cover a wide spectrum of different manifestation of numerical content in terms of writing direction, fonts, font sizes, density of digit placement on the page, etc.

Sun-Zodiac table pages To evaluate to what extent our approach can reproduce the salient relations between the tables in our corpus, we have chosen the sun-zodiac tables, which give the positions of the sun into the signs of the zodiac in degrees for each day of the year. This table was printed in varying layouts, where the different layouts partition the full table differently. In some cases the entire table is contained on one page, in other books, it is distributed over as many as nine pages. The sun-zodiac tables are thus a well-suited example for evaluating our approach in dealing with heterogeneous source material. Moreover, due to its content, the table only comprises the numbers 1 to 31 (max. 31 days per month, and 30 degrees per sign of the zodiac). The table thus only populates a subspace of the feature space that we exploit for our similarity assessments. Since this subspace is more densely populated than would be expected with a uniform distribution of the data over the similarity space, this table is particularly difficult to discriminate under our approach which makes it a good test case.

Two variants of the sun-zodiac table were identified: tables for the times of the "ancient poets" (*veterum poetarum temporibus accommodata*) where the sun is 16 degrees into Capricorn on the first of January, and tables for "contemporary times" (*nostro tempori*) where on the first

day of the year the sun has advanced 3 degrees and is located 21 degrees into Capricorn. This difference amounts to a shift of the columns listing the days of the year with respect to columns giving the angular locations. From the perspective of our similarity model that pools the identified numerical features spatially, these two variations represent the same (more abstract) table.

Altogether, we have identified 68 instances of the sun-zodiac table that cover 250 pages in the corpus. A list of the pages containing the different versions of the sun-zodiac table is provided as `sun_zodiac_pages.csv` along with a ground truth histogram for the digit-features distribution of a prototypical, i.e. noise-free and complete, sun-zodiac table that is provided as `sun_zodiac_hist.csv` in folder `data/corpus` in `code.zip` accessible via https://doi.org/10.5281/zenodo.10933231) (see also Section B.1.2.2 in Supplementary Text).

Climate zone table pages We further collect a subset of material focused on climate zone tables. These tables divide the surface of the "inhabited" world into climate zones defined by the length of the solar day. The zone served as an indication of the general meteorological conditions, which was in turn determinant information in the framework of Medieval and early modern medicine. We find three different principle variants of climate zone tables that use either 7, 9, or 24 climate zones. The 225 pages containing these tables are provided as `clime_tables.csv` (see folder `data/corpus` in `code.zip` accessible via https://doi.org/10.5281/zenodo.10933231). Each row of the csv file corresponds to one individual climate zone table, specifying its variant and providing metadata for the edition containing this table (Section B.1.2.1 in Supplementary Text).

A.8 Limits and Advantages of the Application of Machine Learning and XAI to Historical Analysis

The number of sources analyzed in historical studies is contingent upon the research question and the epistemological approach chosen by historians. Recent trends in historical research

have shifted attention to sources that were largely overlooked in the past, as with university textbooks in our study. The sheer volume of these sources surpasses the human capacity for analysis using traditional methods, especially close reading. Consequently, we propose complementing traditional historical analysis methods with the application of ML techniques. While the need to employ ML thus arises from research questions within the historical disciplines, the application of ML methods might ultimately allow for and prompt new forms of research questions in the future, and thus enrich historical research as discussed in the final section of the main paper.

Our ML-based atomization-recomposition framework holds the potential to unlock further intricate historical analyses in the future, such as understanding the complex interplay between various data including visual, textual, and numerical elements, information related to the materiality of the sources, and social and institutional embeddings of historical actors themselves. Our approach has the potential to expand the possibility of genealogy generation between historical sources without delving deep into the sources themselves; until now, this has remained a very complex and difficult task. Examples include the field of computational stemmatology (*192, 193*) or cases when NLP methods allow historical sources to be related to each other, for example, through stylometric methods (*194*) (Section A.8 in Supplement Materials and Methods). The same atomization-recomposition approach, informed by historical domain knowledge, could also facilitate a data-centric analysis of other complex data types found in historical sources, such as structured mathematical diagrams. These diagrams are abundant in the Sacrobosco Collection and in other works from the early modern period, as well as more generally in mathematical writings from antiquity to the present day. Numerous questions regarding the historical development of such diagrams and geometrical knowledge in general have been addressed in the literature of the philosophy of science and the history of science (*195–199*). While initial efforts to understand these diagrams based on ML methods are underway (*200*), analyzing these highly heterogeneous diagrams, for which high-quality annotated data is scarce,

could benefit from initially atomizing them into basic geometric and textual features (derived from the drawings and their annotations). Subsequently, recomposing them into historically-informed features could open the door to more explainable and grounded predictions.

While this approach opens new doors leading to a better understanding of historical data, it also encounters the general challenges associated with the application of modern ML methods. In particular, the inherent data-dependent nature of models raises the question of out-of-domain generalization abilities, while the high non-linearity of these models further poses challenges regarding model interpretability. However, as demonstrated in our research, these limitations can be effectively addressed so that, by utilizing ML to assist historical investigations, we can also surpass constraints inherent in traditional approaches based on close reading and, specifically, the constraints related to human resources. In the same vein, there are appeals for productively combining computational approaches with traditional in-depth analysis (*201*).

In terms of data dependence, a significant limitation when applying ML methods to historical research lies in the availability of well-curated data. Although we have outlined methods to address the inherent heterogeneity and varied quality of digitized historical source material in this study, our research still depended on a corpus of sources furnished with high-quality metadata. The meticulous preparation of the Sacrobosco Collection took several years and involved two senior historians, two post-doc fellows, and three student assistants. International collaborations further enriched our dataset by bringing additional scholars into contact with it. To apply our methods to other historical inquiries, there is a presumption that historical source data must be similarly enriched and contextualized with metadata.

With respect to the generalization of our ML methods, we have evaluated them according to well-established standards in ML. For each phase of our process, we have presented the relevant evaluation metrics using appropriately selected test sets. For instance, we have quantified the performance of our digit recognition model (Sections A.4.1), the bigram recomposition (A.3.4),

and the clustering performance based on our representation (A.3.4 in Supplement Materials and Methods). This demonstrates that, within our corpus, the applied methods offer satisfactory performance for the intended task. Since ML models can take undesired strategies for making correct predictions (*147*), we have further used XAI methods to ensure that our learned representations do indeed use task-relevant features.

If our approach were to be applied to a different corpus of numerical tables, its ability to generalize to this new dataset would need to be assessed in a similar manner. For example, digit recognition might decrease due to the presence of different printers using unique type fonts. Additionally, the discriminative capability of our representation with respect to tables might be compromised if the distribution of numbers in the tables of the new corpus significantly varies. Should our methodology be transferred not directly to other tables, but in a structural manner to analyze other elements in the sources, such as illustrations, tests akin to the ones used here would be required to gauge the model's ability to generalize to the material at hand.

To deal with the limited availability of labels and overall data instances, our atomization-recomposition approach is designed to reconstruct the information content of the tables up to a sufficient level of representation, i.e., the level of bigrams instead of full numbers. As we have pointed out, achieving complete reconstruction would be nearly impossible due to the absence of annotated data for a fully supervised model. Moreover, the effort to generate such data would be disproportionate to its benefits. Consequently, we only retain the necessary information for our specific objective of identifying similar tables. It is, in part, due to this limitation that our method does not replace but rather complements traditional historical analysis. Based on its representation, our model will never discern the mathematical astronomical "meaning" of a table, by for instance providing the right ascensions for a particular celestial object. However, it can aid a historian who, examining such a table, wishes to locate similar instances amidst vast datasets, in order to study their spatial distribution or temporal occurrences.

Fundamentally, for all the reasons mentioned, ML models will never and are not intended to capture the complete richness of historical sources; they can only represent specific aspects. The choice of these aspects is ultimately driven by the research interests of historians. With these models, however, historians can tackle questions that would be otherwise unapproachable, primarily due to scale constraints. Consequently, these two methodologies must complement each other. When they do, they can invigorate the historical disciplines with novel approaches, methods, and insights as discussed in the final section of the main paper.

If the path outlined in this paper is consistently pursued, it holds the potential to unlock intricate historical analyses, such as understanding the long-term interplay between texts and images. More pressingly, there is the possibility of automatically generating genealogies between texts even before engaging in a thorough close reading. The next ambitious goal, following the current research, is to achieve this using our atomization-recomposition method. This task holds significant relevance within the historical disciplines. A major hindrance so far has been that historical sources often appear in languages or language variants for which no well-curated datasets exist. Our method might help bridge this gap, enabling the identification of pertinent phenomena at the corpus level. Once this is achieved, it paves the way for pinpointing the right clusters of texts that can then undergo a close reading—essentially a case study, informed by a selection made possible through the corpus-level analysis with the assistance of the ML model.

B Supplementary Text

B.1 Insights about the Sacrobosco Collection – General Trend Analyses, Limitations and Case Studies

The potential of our approach is most clearly demonstrated by the fact that it enables, for the first time, an automated investigation of the astronomic tables across the entire corpus of textbooks. It puts us in the position to analyze trends over the large parts of or the entire corpus, and

to reveal geographical singularities or semantic shifts over time. In this way, it offers a new basis to develop case studies and, as will be shown, to reveal unexpected historical findings. We will first present results concerning the general process of astronomy's mathematization as it was taught at European universities between 1472 and 1650, then move to a corpus-level analysis and investigate the mathematization process's temporal and spatial dynamics. After reflecting on the limitations of our approach, we move on to two important case studies that demonstrate its compatibility with standard historical analysis. A general and integrative summary of the historical findings concludes this supplement.

B.1.1 Mathematization of Astronomy in the Framework of Teaching as a Result of Institutional Competition

We start by inspecting the histogram embedding space of the Sacrobosco table pages with regard to additional information about the collection. As this embedding space comprises 110 dimensions, we perform a dimensionality reduction to two dimensions to visually inspect the histogram distribution. This reduction is realized through a t-SNE projection. It is important to note that it is inherently impossible to fully capture the structure of high-dimensional data in just two dimensions, and the limitations of t-SNE have been widely discussed in the literature (*202–204*). Yet, the effectiveness of t-SNE in preserving the original data structure depends on the data itself. If the high-dimensional data is organized on or in close proximity to a non-linear, low-dimensional manifold, the t-SNE projection will preserve the data structure rather well. Moreover, in our inspection of the data distribution, we employ t-SNE projections not as an analytical tool but as a heuristic one. Trends indicated by t-SNE projections are validated by reverting to the original material and analyzing it with the conventional tools of the historian.

First, in Figure S23 (top row), we use the meta-information regarding the publication year and the unique book identifier available for each book to color the t-SNE projected data points accordingly. As visible in Figure S23-a, the visualization using the publication year provides

indications about what pages were printed in proximate time periods *and* are semantically similar, for instance the group of pages on the bottom right. We can further analyze this group by investigating which editions these pages are extracted from as indicated in Figure S23-b and find that these pages stem from multiple books. This allows domain experts to combine different layers of information and to gain corpus-level insights in order to develop hypotheses that can then be investigated further in a targeted analysis.

Second, we can add information from the automated analysis to the visualization. In Figure S23-c, we color code the bigram density on the pages and find that in the t-SNE projection the less dense tables are to be found in the top left corner and center whereas the very dense tables are predominant in the lower right corner. Finally, we show in Figure S23-d the size (number of cluster members) of the cluster that a page was assigned to in a k-means ($k = 1500$) clustering. This tells us that most pages are contained in clusters of less than 30 members, and only a small subset of pages is assigned to larger clusters of around 70 similar tables likely to be found in the low bigram frequency domain.

Historical Interpretation of the Data Distribution in Histogram Embedding Space

The region on the bottom-right of the t-SNE projection of the embedding space shows a high number of table pages clustered closely together suggesting the existence of a group of semantically closely related table pages. We call this area the High-Density-Tables Subset, in short HDT-Subset.

From Figure S23-b, we see that these pages stem from many different editions, and Figure S23.c tells us that they contain tables with a high density around 400 bigrams which are not assigned to great-size clusters as evident from Figure S23-d. Finally, against the background of the year-based projection (Figure S23-a), it is clear that the HDT-Subset of the projected embedding space hosts editions that were published starting from the middle of the sixteenth

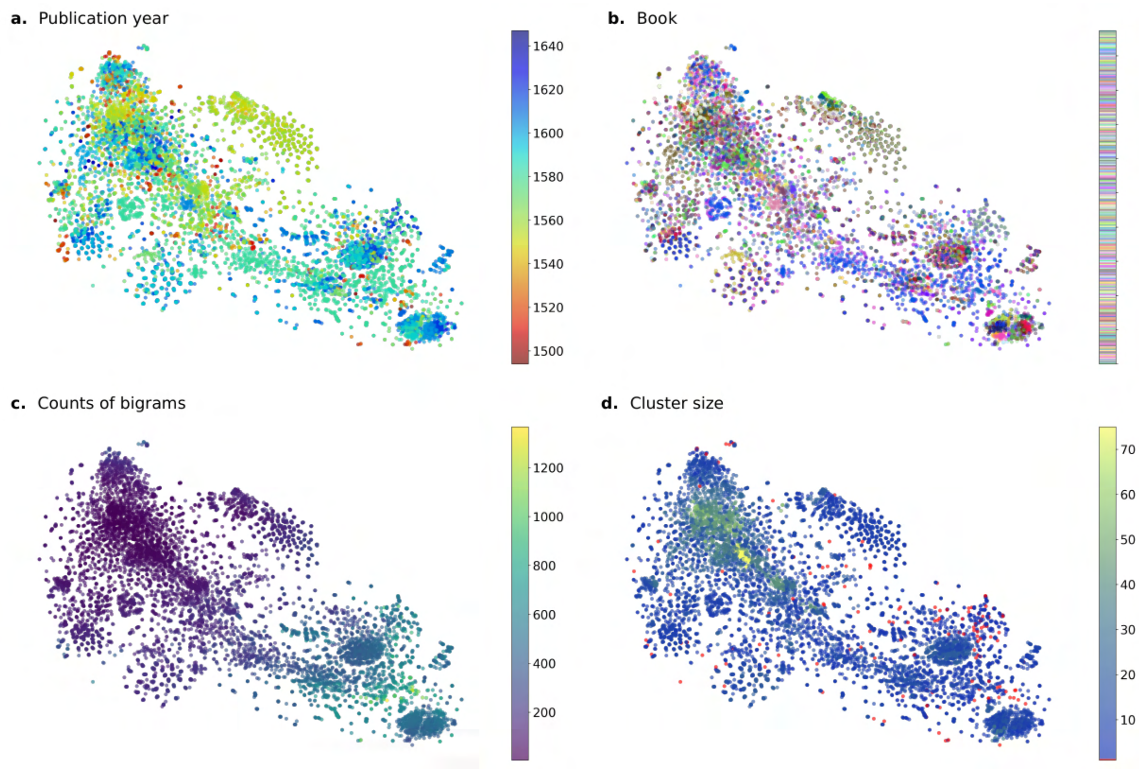

Figure S23: **t-SNE visualizations of the Sacrobosco Tables corpus.** Each data points corresponds to one page and color reflects additional meta-information (top rows) or model-based output (lower rows). We use the available data regarding (**a.**) the publication date and (**b.**) the book title to color each point. The extracted bigram histograms are used to visualize (**c.**) digit density of a page and (**d.**) the size of the assigned cluster for every page.

century and until the end of the historical time interval considered here, 1650. This group was subjected to closer inspection by the historian, excluding five early editions (the orange points on the bottom-left side of the region in Figure S23-a) which were published much earlier than the rest of the editions in the group and which, while close, are situated somewhat marginally with regard to the large group in the projection.

If we consider all tables contained in the editions contributing to the HDT-Subset (Figure S24), it becomes evident that starting around 1585, these tables represent almost the total number of tables published. They are therefore primarily responsible for the acceleration of table publications during this period (Section A.1.1 in Supplementary Materials and Methods).

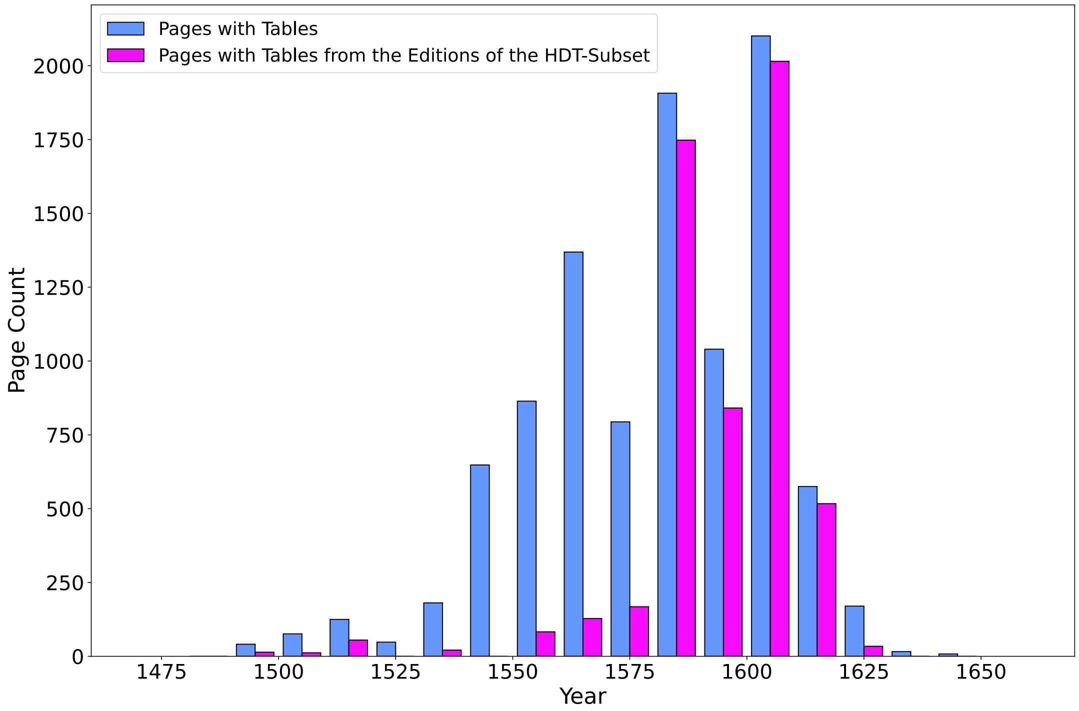

Figure S24: **Pages with tables.** The ratio of published pages from editions containing tables and contributing to the HDT-Subset (magenta) to all published pages containing tables in the entire Sacrobosco Collection (blue).

When considering only the pages containing tables from the HDT-Subset, the percentage of these tables compared to the total number of published tables also quickly increases during the same period. Specifically, the percentages rise from 1.64% for the years 1570–1580 to 3.30%, 10.87%, and 13.85% for the subsequent four decades.

This indicates that the acceleration of table publication that began around the mid-sixteenth century (Section A.1.1 in Supplementary Materials and Methods) eventually resulted in a light but continuous general increase in the rate of homogeneity among these tables and, consequently, among the textbooks. Moreover, stronger phenomena of homogenization are observed in specific temporal phases or geographic locations (Sections B.1.1.1 and B.1.1.2 in Supplementary Text).

Mutual Awareness of Powerful Institutions

By combining these different layers of information, we were able to return to the original data and identify a subgroup of editions that share a great number of semantically similar tables. Examining the group closely reveals that it is constituted by exactly forty editions (see `data/corpus/Metadata_year_tsne_bottom_right.csv` in `code.zip` accessible via https://doi.org/10.5281/zenodo.10933231). The printing dates range from 1551 to 1622. At first sight, these editions appear to be heterogeneous, implied by the fact that they were produced not only across a time interval of over 70 years but also in twelve different cities in Europe. However, upon examining the authors of these textbooks, we observe a peculiarity: thirty-six out of forty editions involve only four scholars. The first five editions, published between 1551 and 1556, are for instance five different texts all compiled by the French Royal Mathematician Oronce Finé and all published by the same printer and publisher, Michel Vascosan, in Paris (*205*). Twenty further editions are commentaries on the original tract of Sacrobosco compiled by the then-leader of the scientific section of the Collegio Romano (Christophorus Clavius), which was the center of the Jesuit Order where scientific knowledge was produced to sustain the innumerable Jesuit colleges all over Europe (*77*). Starting in 1582, we find eight treatises compiled by Thomas Blebel in Wittenberg. The dominant role of Protestant Wittenberg in producing and disseminating scientific knowledge between 1530s and 1560s has already been demonstrated (*59*). Up to this point, the scholar Thomas Blebel has not been investigated by historians of astronomy, but the findings based on our new method strongly suggest that Blebel's works represented an attempt by the Wittenberg community to cope with the works and the success of influential and institutionally powerful scholars such as Finé and Clavius.

Finally, we find three further editions written by the influential late Italian astronomer Francesco Giuntini, who distinguished himself thanks to the introduction of a series of very

long-lasting scientific innovations (*60*) with strong clerical patronage. In conclusion, this region of the embedding space represents clusters of editions generated in the frame of powerful institutions and communities, and the fact that the editions belonging to this region contained almost the entirety of the published tables while their respective amount of similar tables increases means that the communities were observing and imitating each other, possibly due to the influence of cultural and institutional competitions between them. While the t-SNE projection initially suggested grouping and analyzing these editions for commonality, the results of this analysis, as presented above, demonstrate that there is indeed a communal structure shared by these editions. This structure is clearly captured by the distribution of our data representations in embedding space and meaningfully preserved in the t-SNE projection.

As numerical tables in scientific textbooks are the external indication of the process of mathematization (Section A.1.3), we can hypothesize that one of the driving forces of the mathematization of astronomy during the second half of the sixteenth century was an institutional competition that involved the Paris scientific institution expressed by the Royal power, the Protestant leading university of Wittenberg, the Jesuit order, and individual scientists working in a well-protected institutional context.

From a methodological point of view and even without looking at the individual tables contained in these editions (which we do in the next sections), our method allows us to draw historical inferences and reach relevant historical conclusions based on a collocation of material and metadata that otherwise would be non-accessible even to domain experts. Moreover, it can also be stated that our method helps in generating specific historical micro research questions by identifying singularities in great corpora of sources.

B.1.1.1 Corpus-level Analysis 1: Temporal Dynamics of the Mathematization of Astronomy

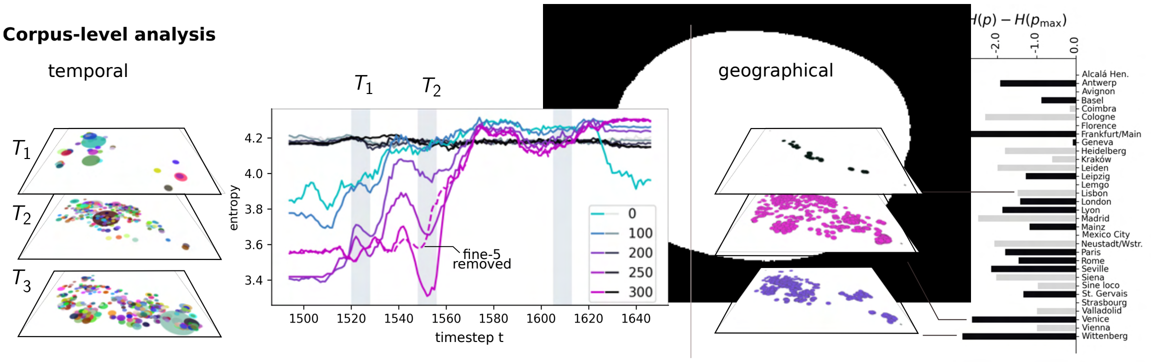

Figure S25: **Corpus-level analysis.** *Left* Evolution of knowledge over time as measured by the entropy of cluster membership vectors (representing the number of tables in each cluster) at each time step. Gray to black lines represent a random embedding baseline, while the colored lines represent data from the Sacrobosco Collection. Different colors indicate filtering thresholds that are based on digit density per page, such as pages with at least 100 digit features. Resulting clusters are visualized using t-SNE for three distinct time intervals, with the disk diameter of clusters scaled according to their number of members. Notably, we detect a significant entropy drop for tables with dense numerical content between 1540 and 1560. This drop vanishes upon excluding the *Fine-5* group, a subset of tables found in editions authored by Oronce Finé, that we have identified as the primary driver of the change in entropy. *Right*: Examining the geographic distribution of knowledge across print locations, sorted alphabetically, using entropy. Low-output cities with less than 100 tables are colored in light gray and t-SNE visualizations for three selected cities are presented.

Moving to the corpus-level analysis, we first investigate the temporal dynamics of the process of mathematization of astronomy during the early modern period by investigating the temporal dynamics of the entropy of the distribution of high-density numerical tables over clusters of similar tables. The editions of the Sacrobosco Collection (1472–1650) that contain at least one numerical table were printed over a time span of 153 years (1494–1647). Throughout this period, publication rates changed considerably, and thus we use a sampling based temporal analysis to assign sampling probabilities using a truncated normal distribution $\mathcal{N}(t_i, \sigma^2)$, setting probabilities for data points outside the interval $(t_i - \sigma, t_i + \sigma)$ to zero. At each iteration, we select $N = 80$ data points, assign a cluster membership label to them and extract cluster count histograms of size $1 \times k$. Finally, the entropy $H(p_{cl}) = -\sum_k p_{cl,k} \log(p_{cl,k})$ of the clus-

ter probability vector $p_{cl} \in \mathbb{R}^{1 \times k}$ is computed. This analysis is computed for the corpus using different digit density thresholds of $\{0, 100, 200, 250, 300\}$, successively filtering pages of low numerical content. For each threshold, entropy evolution is averaged over 20 runs. Results are presented in S25 (left).

In Figure S27 we present additional cluster visualizations throughout the corpus evolution. Disks color codes for cluster membership and their size is proportional to number of cluster members at this time step.

As shown in Figure S27, it remains challenging to discern visually whether significant changes occurred during the transformation of the corpus. Our analysis highlights that when we focus on high-density tables, we uncover significant temporal changes in entropy over time. These changes are far less pronounced if all tables are taken into account. This effect can be traced back to the observation that low-density tables contain less intricate mathematical data, such as enumerated lists, that did not change much over the considered time period. We show exemplary pages grouped by different density levels in Figure S26.

We additionally validate our results against a baseline in which we randomly sample histogram representations $h_{rand} \sim \mathcal{N}(0, 1)$. This serves as a model of a knowledge process that does not consider any change of information or knowledge transfer across printer locations and publication dates and thus is expected not to show any significant entropy changes. We confirm this as presented in Figure S25 (left).

Since the entropy evolution analysis is based on the clustering assignment of pages, we want to control that our results are robust and consistent for a range of reasonable number of clusters k. In Figure S28, we repeat the analysis for $k = \{100, 500, 1000, 1500, 2000, 3000\}$ and observe that if the clusters are sufficiently large for small k, we are not able to observe strong temporal changes as visible for $k = 100$ since the clusters are semantically too diverse. For, $k = 500$ we start to observe the emergence of the entropy drop for high-density pages between 1550

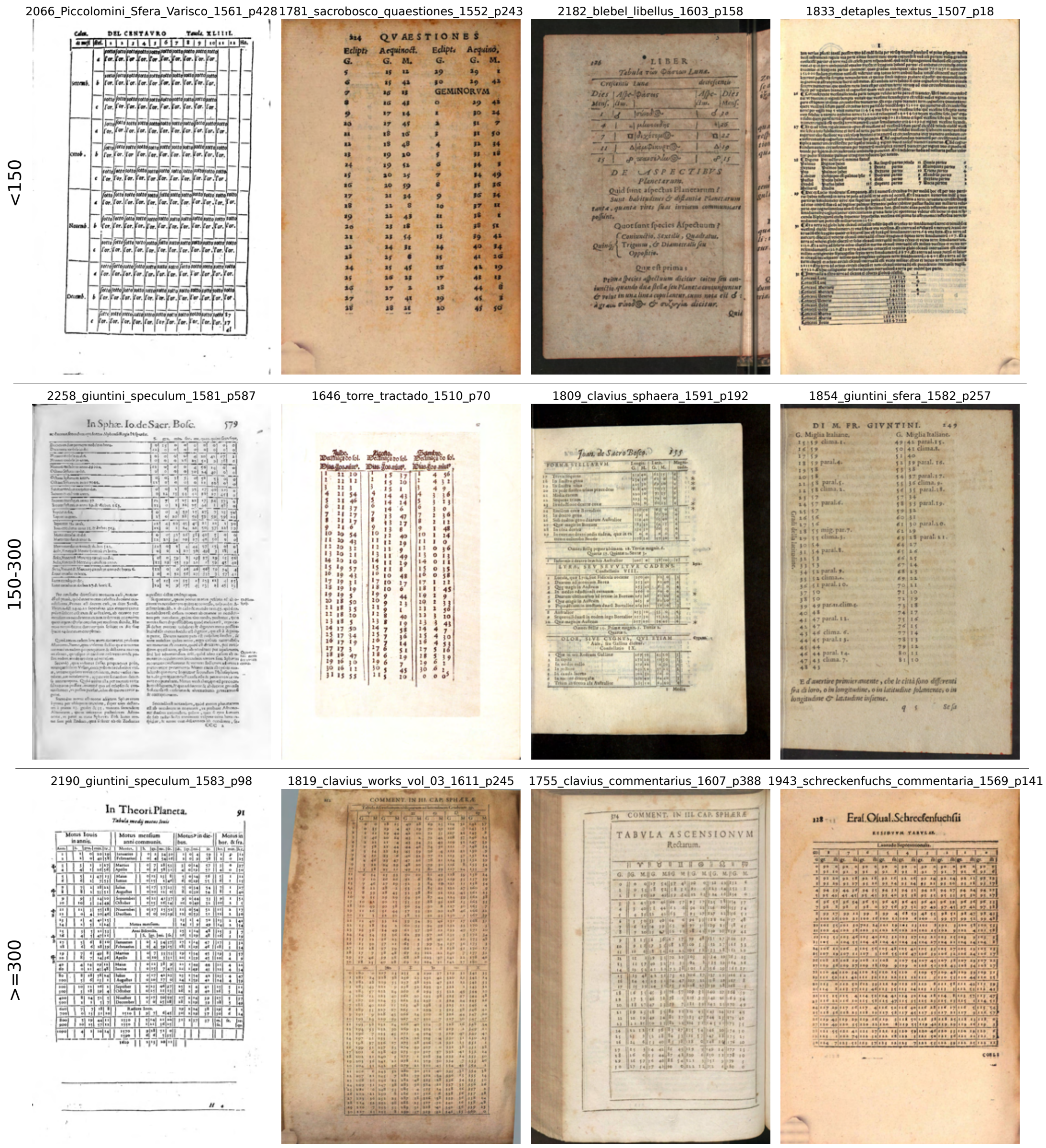

Figure S26: **Sacrobosco Table Pages grouped by digit feature density.** Top to lower rows correspond to increasing digit feature density levels, i.e. the first rows shows pages that contain less than 150 digit features as extracted by the bigram network.

and 1560, which becomes more and more visible for increasing number of clusters. Thus, we conclude that our observation of the entropy singularity is not an artefact of a specific clustering solution but can be observed for a reasonable k-means clustering solutions.

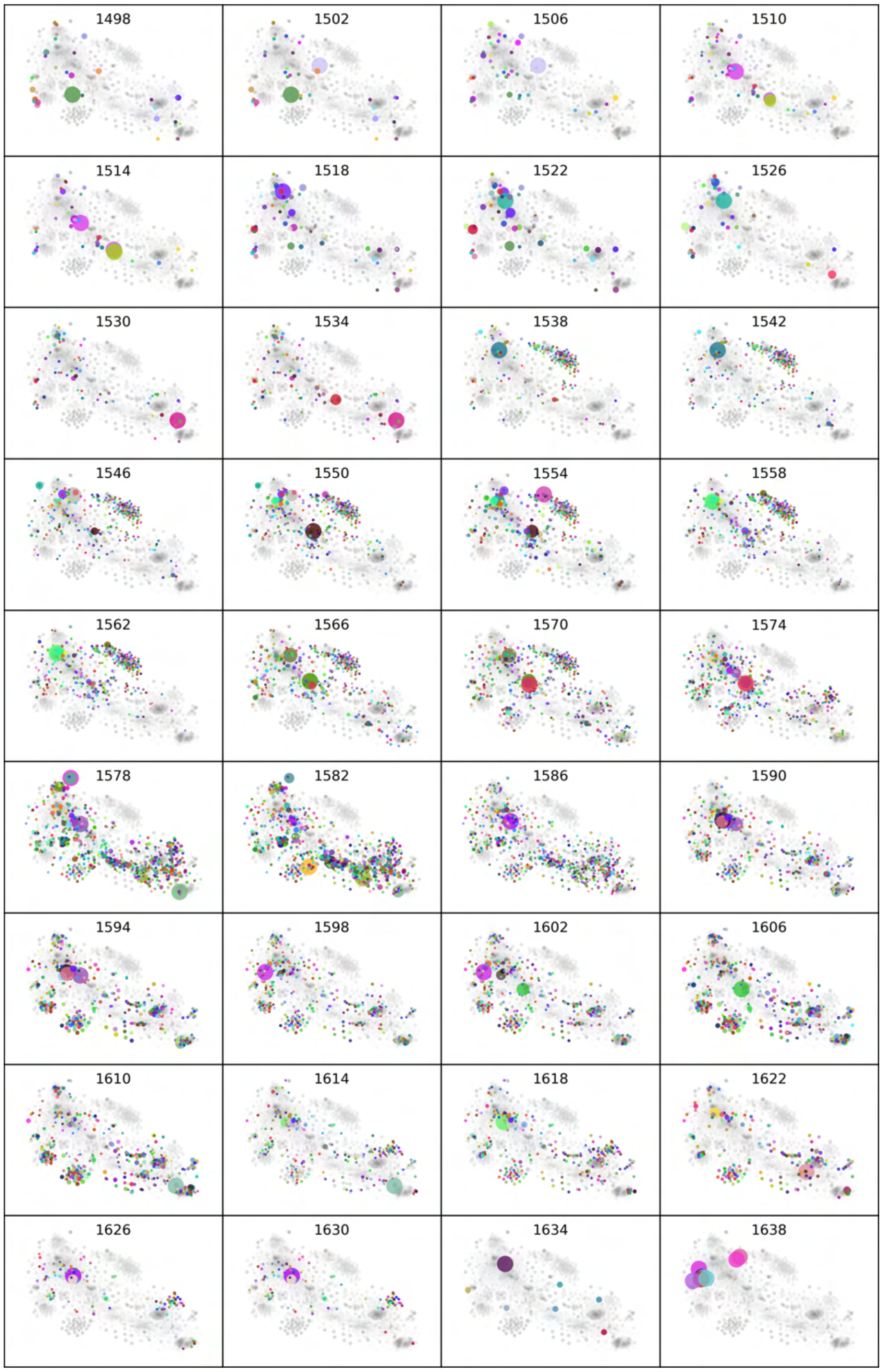

Figure S27: **Temporal dynamics of printing astronomic tables.** Each panel corresponds to one time point of the full corpus evolution. Clusters that contain published pages from this period are represented by a colored disk whose diameter is proportional to its cluster size.

Next, we investigate the effect of the standard deviation σ used to sample pages at each time step t. A smaller σ indicates a more narrow time window used to sample pages from the corpus for a given time step. For $\sigma = \{2, 3, 4, 5, 7, 10\}$ we present the entropy evolution analysis in Figure S29 and observe that for reasonably small σ values the entropy drop is maintained. This is in line with the explanation for the drop which will be advanced below. Only for larger values of $\sigma \geq 7$ we can see that the effect of sampling temporally more distant pages results in a smoothing of the entropy curve and vanishes for $\sigma = 10$.

To consolidate the entropy drop observation we extract the Sacrobosco Table pages that are the main drivers of the entropy change. For this we look at the time between $t = \{1540, ..., 1560\}$ and compute for each time step the clustering distribution $p_{cl,t}$ and entropy $H(p_{cl,t})$. We rank time steps according to the strongest absolute change $|H(p_{cl,t}) - H(p_{cl,t+1})|$ and find that this occurs for $t^* = 1553$. Next, we look at the change in clustering distribution $p_{cl,t^*} - p_{cl,t^*+1}$, rank which cluster has gained or lost the most relative members and historically investigate these relevant clusters and table pages respectively. The analysis reveals that during this period the same work, Oronce Finé's *Sphaera*, was repeatedly reprinted in five books which we refer to as the *Fine-5* group, constituted by three Latin editions (*206–208*) and two French ones (*209, 210*).

Historical Interpretation and Confirmation of the Temporal Corpus-Level Analysis

The observed lower entropy scores can suggest that the distribution of scientific knowledge within numerical tables aligns more closely with previously published material and/or that newly introduced tables are semantically related. In contrast, higher entropy scores could indicate a more uniform distribution of published content. The first aspect shown by the temporal analysis is the drop of entropy starting in 1551 in a rather short time window of 3 to 5 years. To assume a rather drastic development in such a short time window becomes historically plausi-

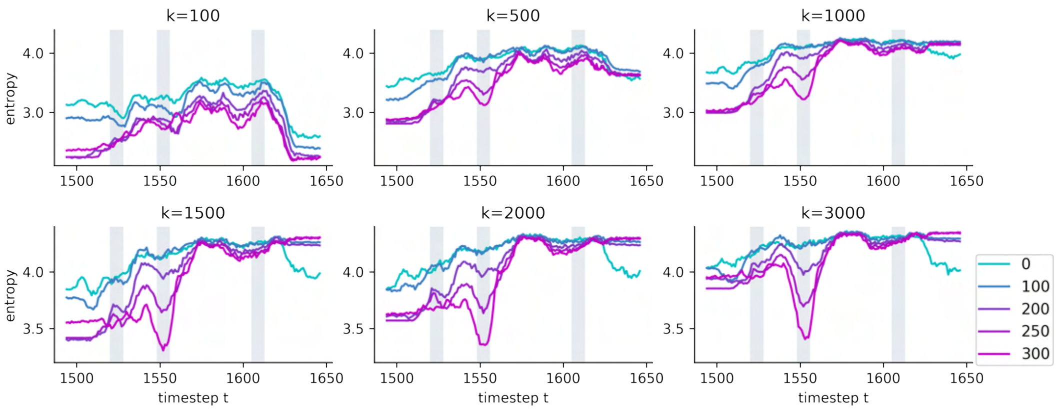

Figure S28: **Control study for different numbers of clusters.** Entropy evolution for different number of clusters $k = \{100, ..., 3000\}$. The different line colors correspond to a digit density filter of the pages, e.g. using all pages that contain at least 100 bigram features.

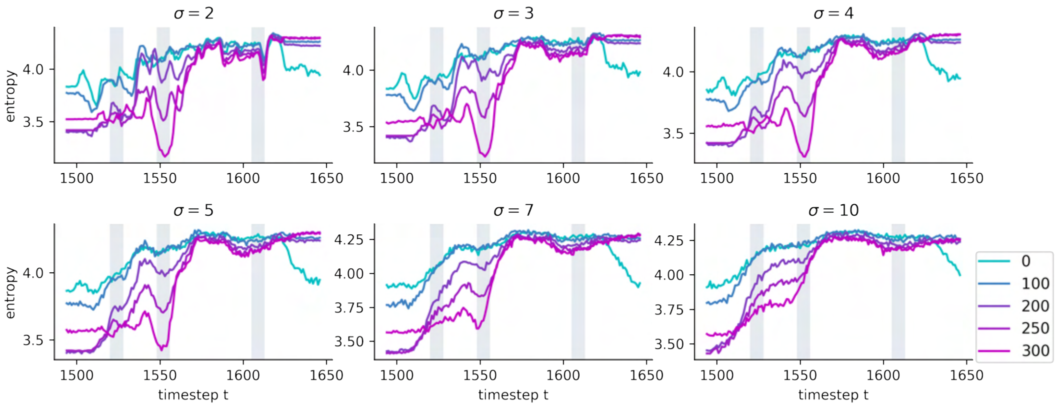

Figure S29: **Control study for varying time windows.** Pages are sampled from a Gaussian distribution centered at current time step t with a standard deviation of $\sigma = \{2, 3, 4, 5, 7, 10\}$.

ble if we take into account the practice of the printers and publishers comprising the emerging academic book market during the sixteenth century. When a novelty was introduced, printers and publishers could obtain a so-called *privilegium* upon request. A *privilegium* is the forerunner of what is now called copyright, and as it applied only to the book as a product, it was originally conceived to protect the work of print-shops's owners. However, a *privilegium* was often valid for only a few years, usually only two. This implies that once a new treatise was

granted a *privilegium*, the printer and/or publisher had every incentive to saturate the market with the that treatise. A normal practice, which also had the advantage of limiting the financial damages caused by the second-hand market, was to produce a large print-run (which in itself was a way to lower the per copy production costs) and then to place portions of the same print-run on the market every year. Only a new title page, with a new date of publication, had to be freshly printed in order for the printer/publisher to be allowed to claim they were publishing a new edition. The new edition was therefore not really a re-print, but more properly speaking a re-issue. Moreover, because of the fact that books were not sold bound, as they are today, but as piles of printed sheets that were then folded and bound at the book shop, printers and publishers always had the possibility to replace or add just a few sheets in order to claim that the new edition was indeed "really" new. For these reasons, frequently a new treatise, with high potential for international success, was pushed into the market by means of a series of editions published during a relatively short time interval, as has been demonstrated specifically for the Sacrobosco Collection and in reference to the the economic rules of the academic book market during the early modern period more broadly (*58, 78, 79*).

By looking closer at the group *Fine-5* that our corpus-level temporal analysis has identified as responsible for the entropy drop, it indeed turns out that, following the market mechanisms of printing outlined above, the five editions are in fact one Latin edition of 1551, a related re-issue in 1552, a slightly changed re-print of the same in 1555, a French edition of the same book also published in 1551, and a related re-issue in 1552. Moreover, these works also belong to the bigger cluster that involves forty editions and discussed in Section B.1.1 of the Supplementary Text. Bringing together these two findings enables us to conclude that the institutional competition mentioned earlier, which was a harbinger of the process of mathematization of astronomy, made use of commercial mechanisms developed within the context of early modern book marketing (*80*). This has not adequately been comprehended before.

Moreover, there is a noticeable trend of increasing entropy up until around 1570, at which point it reaches a saturation point. We know that all the editions of the collection focus on the same core knowledge and are printed in an increasing number of places, reaching an ever-widening audience in this period (knowledge homogenization). At the same time, however, the entropy trend during the initial 100 years means that novel content attaches to this common core in different ways, such as in processes of innovation. Finally this implies that the process of mathematization and the spread of scientific innovations went hand in hand with the process of homogenization of scientific knowledge. This important historical and epistemological result will be deepened by the successive corpus-level analysis presented in the next section that concerns the variance in the spatial distribution of the process of mathematization of knowledge as represented by computational tables.

B.1.1.2 Corpus-level Analysis 2: Spatial Variance of Mathematization of Astronomy

Here, we compare knowledge production across the 32 different printing centers. An overview of the distribution of cluster membership for the most productive print centers is shown in Figure S30. Different locations adhered to different printing strategies, e.g. by printing novel content or by adopting and reprinting content first appeared in other places. In the following, we show how to quantify these phenomena within an information-theoretic framework, enabling a comparatistic analysis of different printing strategies.

For each city, entropy scores are presented in Figure S25 (right). This score, defined to be below or equal to zero, captures the difference of entropy between the observed cluster distributions and an uninformed uniformly distributed production process $H(p) - H(p_{\max})$ with p_k being the probability of assigning a table to cluster k and $H(p_{\max}) = log(N_c)$ with N_c denoting the number of tables printed in city c.

The entropy $H(p_{\max})$ expresses the entropy of a hypothetical and unrestrained production

process, i.e. without references or memory of previously printed material, and without following an agenda imposed by external factors. In this setting, the produced table corpus is expected to not contain a specific similarity structure.

The entropy score thus compares the actual production and distribution process of historical content against this hypothetical process, highlighting print locations that have produced redundant information versus locations that have produced original content. While a certain degree of redundancy can be considered a necessary precondition of stable and successful knowledge transmission, too high a redundancy would mean stagnation, as is does not leave room for novelty. Our analysis in Figure S25 (right) highlights that entropy varies strongly across locations. A minimum in entropy is reached for the cities of Frankfurt am Main and Wittenberg. This indicates that in Wittenberg and Frankfurt, many tables are formed around the same clusters in comparison to an unconstrained print output. The respective textbooks produced in these cities are also more similar to one another than to material produced across the other locations, indicating a more homogeneous production process.

In Figure S31, moreover, we provide a t-SNE visualization for each of the different print locations in the corpus, providing indications of the content variety and output quantity of the different cities. The t-SNE mapping used here is the same as that in Figure S23. While Alcalá de Henares, Strasbourg, Lemgo, Vienna, Mexico City, and Avignon have each printed less than five table pages, the most productive print centers were Lyon, Venice, Wittenberg, Rome, Frankfurt (Main), Paris, and Saint Gervais with at least 500 printed table pages. We assume that similar distributions of the pages in the t-SNE signify locations that exhibit a similar print program, an assumption which is then checked against the original data. For example Rome, Saint Gervais, and Geneva have printed content that covers comparable regions of the embedding space as visible in Figure S31. In parallel, Lyon and Venice show the widest coverage across the full embedding space.

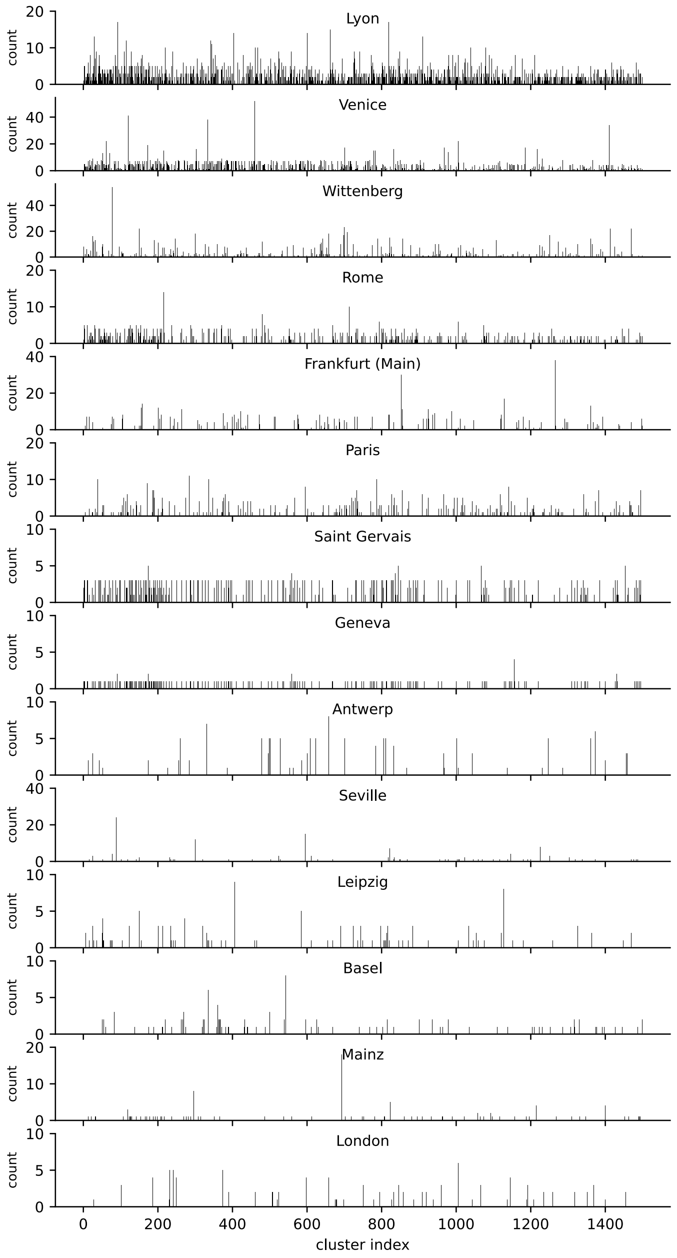

Figure S30: **Cluster membership histogram for each print location.** Displayed histograms show the number of printed table pages per cluster for all cities that have produced at least 100 pages (sorted from most to least productive print location).

Historical Interpretation and Confirmation of the Spatial Corpus-Level Analysis

The corpus-level analysis matched with further geographical data has provided two results. The first concerns the homogeneity of the output of tables in the treatises produced in Wit-

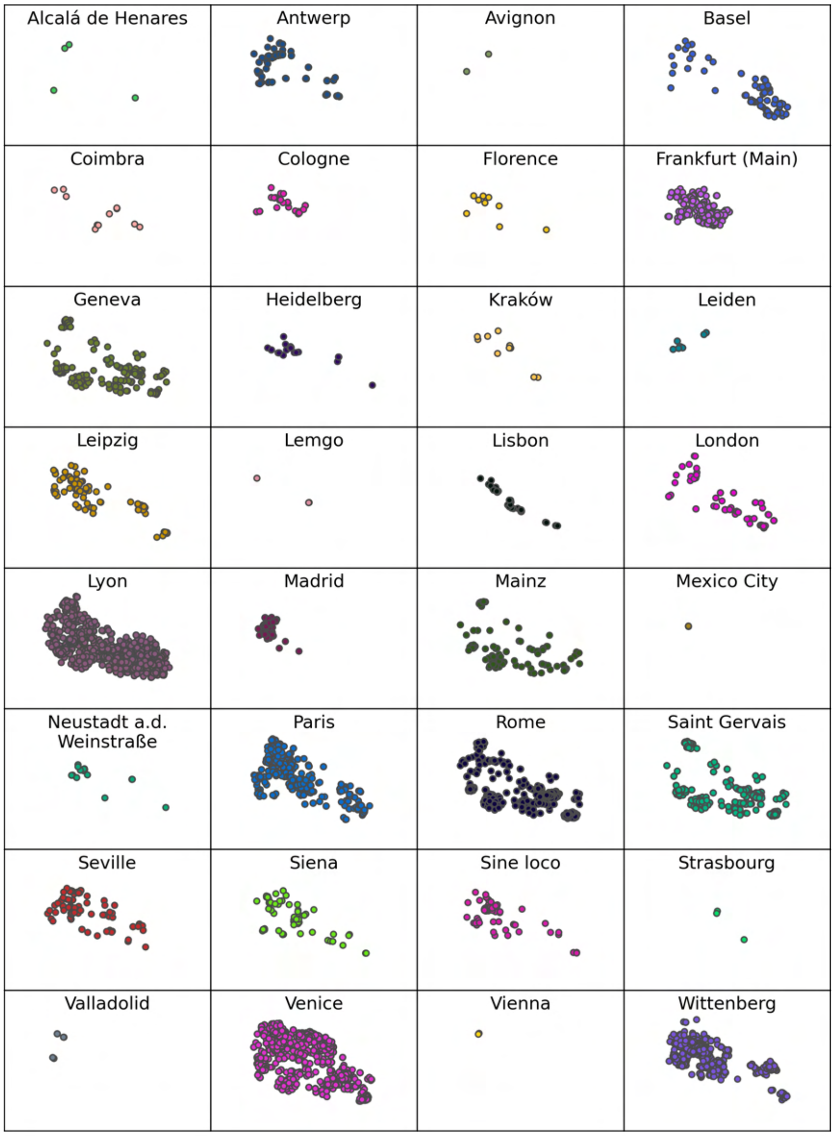

Figure S31: **Geographical distribution of printed table pages.** Each point corresponds to one page from the Sacrobosco Tables Collection and locations are sorted alphabetically.

tenberg and Frankfurt am Main. The second concerns the similar t-SNE projections for some places of publication such as Geneva, Mainz, and Saint Gervais, as well as the dissimilar features for instance of the projection for Venice.

Regarding the first point, historical analysis confirms that the lower entropy in Frankfurt

can be attributed to a significant portion of its book production consisting of numerous reprints of the same edition. This exemplifies redundant production, resulting in a high number of duplicative editions. Out of a total of seventeen editions produced in Frankfurt, thirteen are reprints of two specific editions, indicating that only four editions out of the seventeen are distinct. The first series of reprints spans from 1549 to 1571 (*211–220*), while the second series extends from 1580 to 1600 (*221–224*).

In Wittenberg, however, the case is different since it is known that key figures of the Protestant Reformation, Martin Luther and notably Philipp Melanchthon, were involved in the meticulous design and oversight of the curriculum of study at the Wittenberg University (*110, 256–257*) (*83, 84*). They had further worked in close contact with numerous printers and publishers that had moved their businesses to Wittenberg after the Reformation (*225*). Finally, there is a text written by Melanchthon, the famous *praeceptor Germaniae* ("Germany's instructor") calling for students to study mathematical disciplines and especially cosmology and astronomy, first printed in 1531 and reprinted at least another 63 times until 1619 (*226*). Considering the results and analyses that we have gained using our model, we can now surmise that the homogeneity observed in the mathematical content of the treatises produced in Wittenberg may have been a consequence of the political oversight exercised by the Protestant Reformers, who were most certainly aware of the wide-spread influence wielded by scientific treatises from Wittenberg all over Europe, as mentioned above in Section A.1.2. This interpretation further aligns with and is supported by the fact that the scientific visual apparatus used in the field of astronomic studies, which were mostly developed in Wittenberg during this era, maintained a steady influence across Europe that endured for many decades (*82, 95*). This suggests that the scientific output of the recently reformed Wittenberg had a significant influence on the linked processes of mathematization and homogenization of knowledge throughout Europe.

The cases of Frankfurt and Wittenberg, moreover, show how easily our model and suggested

analysis can identify singularities within a large volume of historical sources.

Coming to the second analysis, we focus on the projections for Mainz, Saint Gervais, and Geneva, as they clearly show a similar distribution in the t-SNE. Again in this case we do not take the structure observed in the t-SNE at face value. Instead, we identify by means of a simple database query for those places of publication the relevant data, and more closely inspect the treatises that are mostly responsible for the pattern observed in the t-SNE projection. We immediately discover that the great majority of treatises produced in these locations are actually many different editions of the same commentary, possibly slightly changed over time. These are the treatises authored by Christophorus Clavius, namely those treatises that were also identified by means of a closer look at a specific cluster suggested by the general t-SNE projection and discussed in section B.1.1 in Supplementary Text. This finally is a further confirmation of the overall picture of a series of centers and scientists in competition against each other to conquer the European academic book market by pushing the discipline toward a more sophisticated level of mathematization.

B.1.1.3 Limitations of Data-Driven Historical Analysis

Although methodological advances in machine learning have the potential to enable the processing of historical sources at scale, their applicability depends on a clearly defined task-setting and a critical assessment of the statistical properties of the data, as discussed in Section A.8 in Supplement Materials and Methods.

Assuming a robust ML method, this would allow a fully data-driven generation and selection of historical hypotheses. Instead, we adopted a combined approach: data-driven extraction and visualization of feature representations at the corpus level, complemented by decision-making and selection of relevant use cases guided by expert knowledge.

Historical data is always incomplete, not only due to losses in historical transmission but

also because of the limited resources available to digitize historical evidence in an appropriate form and adequate quantity. Therefore, the selection of historical data processed in computational approaches is ultimately and necessarily always based on assumptions by domain experts. Without a way to feed these assumptions back into the analysis, purely data-driven approaches will suffer from not being able to account for the fact that the data distribution is influenced by both the historical process and the process of data selection.

The challenges of a fully data-driven approach can be illustrated by a visualization of cluster membership vectors over time, as shown in Figure S32. Clusters are sorted from the smallest to the largest number of table pages they contain, alongside the publication year. The resulting distribution clearly exhibits a structure that suggests specific groupings of similar print patterns. A data-driven approach here would explore this structure, apply an adequate clustering method to robustly establish the suggested groups and integrate this with the contextual data, thus arriving at a hypothesis generated in a data-driven manner.

This would put in focus the structure displayed in Figure S32, which, however, does not yet entirely display historically meaningful clusters, as discussed in the following. The layout of a given table in a given edition can be such that it stretches over a number of pages. When such an edition is reprinted or re-issued with the same layout, the result is represented by distinct clustering patterns where the first page of a table will be clustered with the first page of that table in successive editions, the second table page with the second in successive editions, and so on for all tables in the edition. The apparent structure in Figure S32 largely stems from this republication process, emphasizing the need for domain knowledge to curate the historically and semantically meaningful clusters.

This remarks on the limitations of data-driven historical analysis tie into and complement the observation in Section A.8 once more underlining that computational and traditional methods should and must complement each other in the analysis of historical sources.

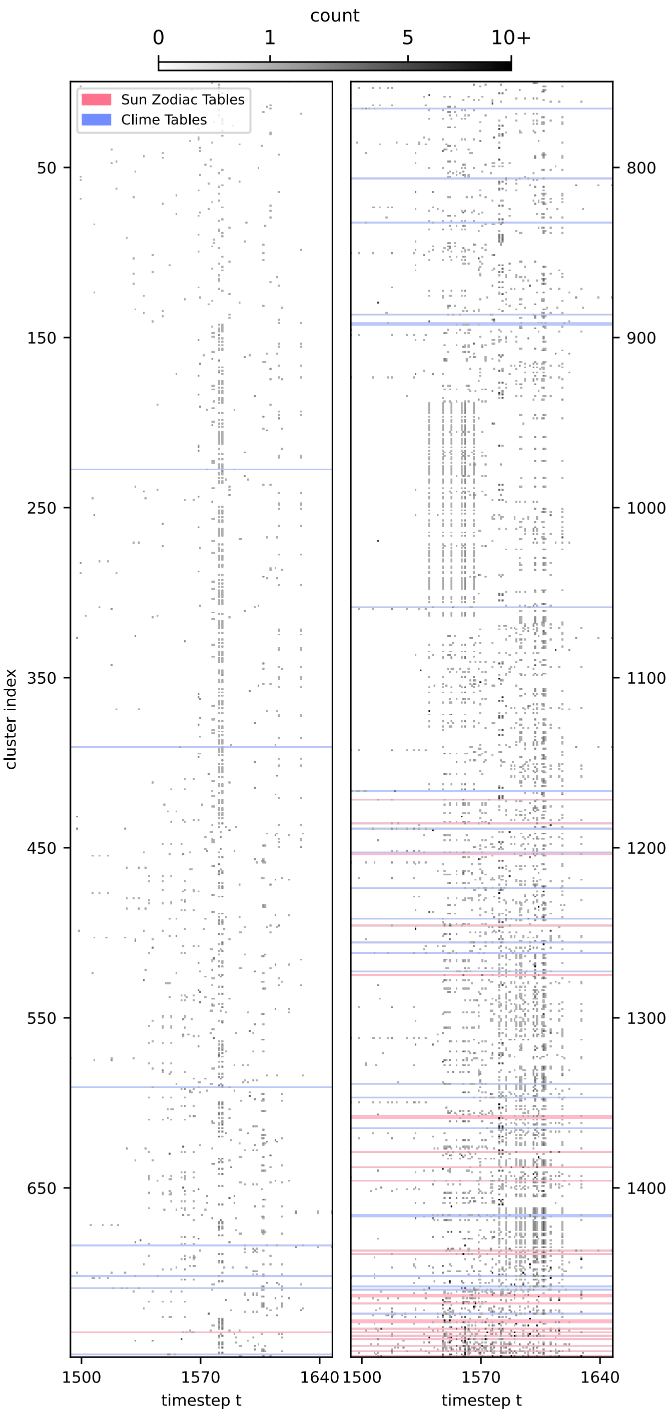

Figure S32: **Cluster membership counts over time.** Clusters are sorted by size and an each colored row indicates a cluster containing at least one Sun Zodiac (red) or Climate Zone (blue) table page.

B.1.2 Historical Case Studies and Table Similarity

While the primary goal of our approach is to obtain an overview of all the available materials, our model also allows for a different approach that focuses on specific and detailed interests. Thus, if for instance a historian wants to analyze the spread of a specific table—identified either through close reading of the text or because of its position within a particular cluster in the embedding space—we have developed a tool to input the image of that table in order to identify all similar tables (Section "Data and Code Availability" in the main paper). This creates a group of tables that, once matched with metadata, provides all necessary information to the historian in order to conduct specific case studies. The tool enables users to query the corpus and to find all tables in the corpus similar to a query table they provide, either in original format (page scan) or as a ground truth histogram. From a historical-methodological perspective, this approach implies that machine learning first enables us to conduct a corpus analysis which then serves as the backdrop against which case studies are selected. In other words, the relationship between micro and macro history is further enriched by the possibility to move in both directions from one to the other level.

In the following, we present two case studies as examples of such an approach, one concerned with the *Climate-Zones* tables and the other with the *Sun-Zodiac* tables. At the end it will be shown how these two very different case studies together allow for a general historical contribution.

B.1.2.1 Historical Case 1: Tables of Climate Zones

In his *Tractatus de sphaera* Sacrobosco picks up on a topic that has its origins in ancient Greek astronomy and geography, namely the subject of the different climes or climate zones as they will be referred to in the following. The climate zones quite generally divide the surface of the "inhabited" world into regions or bands bordered by circles of the same latitude. Climate

zones are formally defined by the length of the solar day which is used as an indication of the overall meteorological conditions that were, in turn, determinant information in the framework of Medieval and early modern medicine. Sacrobosco's discussion of the climate zones is found at the end of chapter 3 of his treatise where he introduces the concept and provides the essential data defining seven climate zones. The original treatment by Sacrobosco is picked up in the majority of the books in our corpus. More often than not, the data for the climate zones, which Sacrobosco himself renders as a text, is presented in form of a table. Our approach has enabled us to identify all climate-zone tables in the corpus, forming the largest cluster of tables overall. By matching these tables with the bibliographic metadata of the textbooks from which they are extracted, we indeed find that this cluster is the most extensive both spatially and temporally. We refer to Section A.7.2 in the Supplement Materials and Methods for the complete list of tables that were computationally extracted from the corpus and amended by a human expert.

Due to the inherent characteristics of our model, we are able to identify not only tables that contain data on the seven climate zones, but also tables that are similar, for instance sets of tables that contain additional information. These tables reveal the introduction of a novelty with respect to Sacrobosco's original treatise, which represented ancient and medieval knowledge. Besides tables listing the seven climate zones, the corpus contains instances of tables expressing a division into nine climate zones as well as tables representing a division into twenty-four climate zones. In the following we will analyze this finding and the spread pattern of these three variants' occurrences as evidenced by our collection and attribute historical reasons for those patterns.

The Tradition Of the Climate Zones

Clima in the ancient Greek tradition initially simply meant inclination and, if applied in geography, it specifically expressed the latitude of a location on earth. In Ptolemy's time (1st

cent. BCE), *clima* was indeed predominantly related to terrestrial latitude. Latitude circles were usually referred to as parallels (i.e. circles parallel to the equator).

For places with the same latitude, numerous observable astronomical phenomena are the same. For instance the length of the day is the same everywhere at the same latitude and thus in particular so is the length of the longest day of the year at the summer solstice. Before the introduction of a latitude grid, a common way to express quantitatively the latitude of a place was to specify the length of the longest solar day at that place (*227, 23*).

In the second century BCE, Hipparchus had already furnished the mathematical relation between the length of the longest day specified in hours and the latitude specified in degrees. Initially, the expression *clima* seems to have been used for any latitude expressed by the length of longest day. We note that Otto Neugebauer has rightly remarked that measuring the day-length instead of simply the pole height is much more complicated and less precise. He speculates that specification of latitude by day-length was practiced nevertheless because of its greater practical relevance ((*227, p. 23*)).

In the *Almagest*, Ptolemy included a list of parallels for increasing day-lengths of the longest day from 12 to 24 hours, beginning with 15 minute steps near the equator and in half-hour steps further away. Seven of Of these parallels he marked explicitly as *climata* and then specified, in addition to their latitudes in degrees, the city or some notable geographical feature the respective parallel runs through. This system, which Ptolemy later included in his *Geography*, was soon accepted as canonical and it radically changed the meaning of the term $\kappa\lambda\iota\mu\alpha$.

Besides the association with the parallel, *Clima* thus also came to acquire the meaning of "region," i.e a belt or zone of certain width around a particular latitude circle in which certain celestial phenomena "do not change appreciably." This explains why the words "clime" could be used either in the specific sense of the parallel or more generally for the band around it, as from most perspectives there was not a practical difference. It was moreover alleged that

the climate and related phenomena such as vegetation were similar within these zones (*228*). The seven zones subdivided the portion of the Earth's surface that was considered habitable in antiquity, though it was already known that people also lived outside that zone. As life outside of the defined habitable surface portion was considered uncomfortable because of excessive heat or cold, the other zones were then just ignored. No particular climate zones were specified for the southern hemisphere. The literature shows a dispute regarding the origin of the understanding of clime as climate zones. According to Ernst Honigman and Fuat Sezgin (*229*), this idea traces to Eratosthenes or earlier. David R. Dick argues for it to have originated with Hipparchus in *Posidionius* (*230*).

Sacrobosco himself harks on a tradition transmitting knowledge concerned with the climate zones as defined by Ptolemy: seven clime parallels marking their centers and are bordered by parallels defined by the longest day being respectively 15 minutes shorter or longer. In the *Phases of Fixed Stars and Collection of Weather Changes*, Ptolemy himself only uses five *climata* (cf. (*231*)). Thus, the first clime was understood as defined by the parallel for the maximum day-length of 13 hours as its center, the parallel for the day-length of 12 hours and 45 minutes as its southern and that of 13 hours 15 minutes as its northern confine. At the same time, this northern limit was the southern confine of the second climate zone, i.e. the seven zones where perceived as as being directly adjacent. Primarily via their number, the seven climate zones were related to the seven planets and thus also assumed astrological and therefore medical significance. The climate zones understood in this way "became one of the basic, canonical elements of late antique medieval European and Arabic geography" and as such were also picked up by Sacrobosco (*227*).

The Climate-Zone Tables in the Sacrobosco Collection

Seven climate zones. With the help of our approach we could identify 117 tables containing data concerned with only the seven climate zones. These tables show different attributes in various columns, though the sorts of attributes and the number of columns in a table can considerably vary, as the examples in figures S33 and S34 clearly show. Usually, a column displays the latitude given in degrees and minutes. Almost always a column is present that shows data of the length of the longest solar day given in hours and minutes at a specific latitude. In the majority of cases there is also a column naming a place which the central parallel of the zone runs through. Moreover, there can be a column for the width of the zone given as an angle and/or arc length of the zone sector. If present, the arc length is specified with respect to different units in different tables (e.g. stadia, German miles, etc.) and hence the numbers in this column, if present, can vary greatly.

For each climate zone, the parallel marking its southern confine, its center parallel and the parallel marking its northern confine are given. Sometimes the latter is omitted as it coincides with the beginning of the next climate zone. Usually, the parallels are given as rows, but there are other layout options working for instance with additional columns (Figure S34). As mentioned above, the seven climate-zone table represents the main scientific tradition during the early modern period, a tradition that originated in antiquity and continued throughout the Western Middle Ages and the Islamicate culture (Fig. S44).

Nine climate zones. These tables extend the type "seven" tables by two additional climate zones to the north. They re-occur at a much lower rate, with just twenty-six instances in total in the Sacrobosco Collection, and the layout of these examples is much more homogeneous than in the case of the seven climate-zone tables. Figure S35 shows the typical layout: Initial medium and end parallels are defined by day-length and longitude, and these pieces of information are

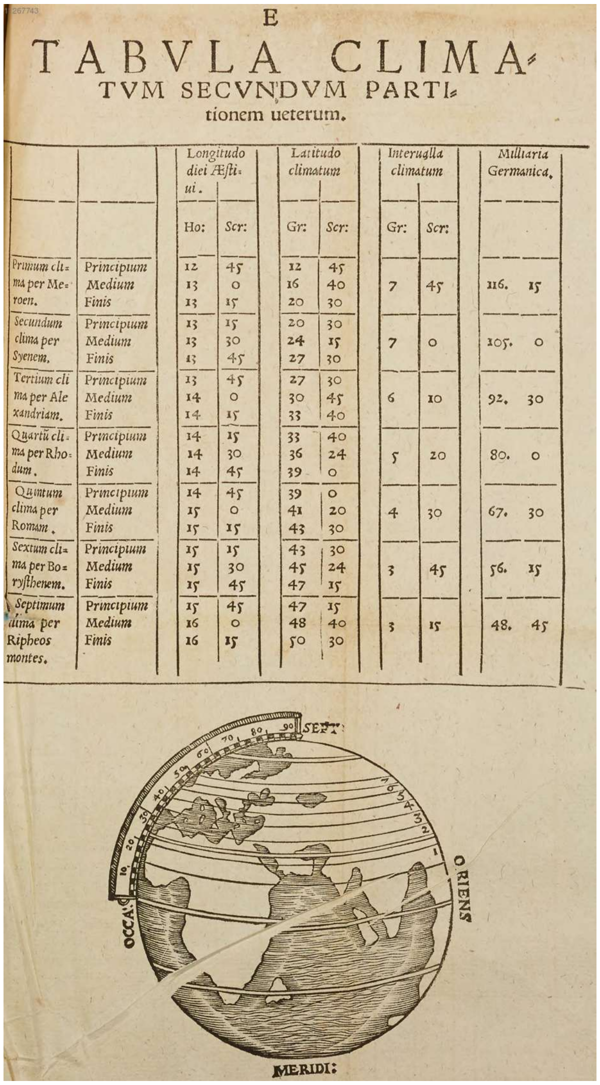

E

TABVLA CLIMATVM SECVNDVM PARTItionem ueterum.

		Longitudo diei Æstiui.		Latitudo climatum		Interualla climatum		Milliaria Germanica.
		Ho:	Scr:	Gr:	Scr:	Gr:	Scr:	
Primum clima per Meroen.	Principium	12	45	12	45	7	45	116. 15
	Medium	13	0	16	40			
	Finis	13	15	20	30			
Secundum clima per Syenem.	Principium	13	15	20	30	7	0	105. 0
	Medium	13	30	24	15			
	Finis	13	45	27	30			
Tertium clima per Alexandriam.	Principium	13	45	27	30	6	10	92. 30
	Medium	14	0	30	45			
	Finis	14	15	33	40			
Quartũ clima per Rhodum.	Principium	14	15	33	40	5	20	80. 0
	Medium	14	30	36	24			
	Finis	14	45	39	0			
Quintum clima per Romam.	Principium	14	45	39	0	4	30	67. 30
	Medium	15	0	41	20			
	Finis	15	15	43	30			
Sextum clima per Borysthenem.	Principium	15	15	43	30	3	45	56. 15
	Medium	15	30	45	24			
	Finis	15	45	47	15			
Septimum clima per Ripheos montes.	Principium	15	45	47	15	3	15	48. 45
	Medium	16	0	48	40			
	Finis	16	15	50	30			

Figure S33: **Seven climate zone table.** From: (*232, sign. Q4-8*). Augsburg, Staats- und Stadtbibliothek, Uniform Resource Name: urn:nbn:de:bvb:12-bsb11267743-1.

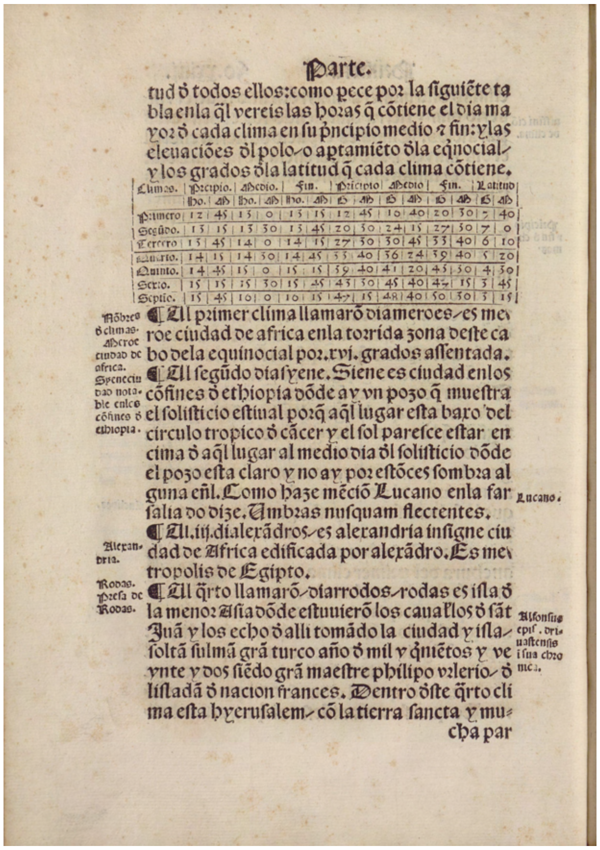

Parte.

tud ꝺ todos ellos: como pece poꝛ la siguiẽte tabla enla ꝗl vereis las horas ꝗ cõtiene el dia mayoꝛ ꝺ cada clima en su ꝑncipio medio ⁊ fin: y las eleuaciões ꝺl polo/o aptamiẽto ꝺla eꝗnocial/ y los grados ꝺla latitud ꝗ cada clima cõtiene.

Climas.	Pricipio.		Medio.		Fin.		Pricipio		Medio		Fin.		Latitud	
	Ho.	M	Ho.	M	Ho.	M	G	M	G	M	G	M	G	M
Primero	12	45	13	0	13	15	12	45	16	40	20	30	7	40
Segũdo.	13	15	13	30	13	45	20	30	24	15	27	30	7	0
Tercero	13	45	14	0	14	15	27	30	30	45	33	40	6	10
Quarto.	14	15	14	30	14	45	33	40	36	24	39	40	5	20
Quinto.	14	45	15	0	15	15	39	40	41	20	43	30	4	30
Sexto.	15	15	15	30	15	45	43	30	45	40	47	15	3	45
Septie.	15	45	16	0	16	15	47	15	48	40	50	30	3	15

Nõbres ꝺ climas. ¶ Al pꝛimer clima llamarõ ꝺiameroes/es meroe ciudad ꝺe africa enla toꝛrida zona ꝺeste cabo ꝺela equinocial poꝛ.xvj. grados assentada.
Meroe ciudad ꝺe africa.

Syene ciudad notable enlos cõfines ꝺ ethiopia. ¶ Al segũdo ꝺiasyene. Siene es ciudad enlos cõfines ꝺ ethiopia ꝺõde ay vn pozo ꝗ muestra el solisticio estiual poꝛꝗ aꝗl lugar esta baxo ꝺel circulo tropico ꝺ cãcer y el sol paresce estar encima ꝺ aꝗl lugar al medio ꝺia ꝺl solisticio ꝺõde el pozo esta claro y no ay poꝛ estõces sombꝛa alguna eñl. Como haze mẽciõ Lucano enla farsalia ꝺo ꝺize. Umbꝛas nusquam flectentes. Lucano.

Alexandria. ¶ Al.iij. ꝺialexãdros/es alexandria insigne ciudad ꝺe Africa edificada por alexãdro. Es metropolis ꝺe Egipto.

Rodas. Presa ꝺe Rodas. ¶ Al ꝗrto llamarõ/ꝺiarrodos/rodas es isla ꝺ la menoꝛ Asia ꝺõde estuuierõ los caualłos ꝺ sãt Juã y los echo ꝺ alli tomãdo la ciudad y isla/ soltã sulmã grã turco año ꝺ mil y ꝗnietos y veynte y ꝺos siẽdo grã maestre philipo vıꝛlerio/ ꝺ lisladã ꝺ nacion frances. Dentro ꝺste ꝗrto clima esta hyerusalem/ cõ la tierra sancta y mucha par
Alfonsus epis. oriuastensis i sua chronica.

Figure S34: **Seven climate zone table.** From: (*233, p. XXIIIIv*). Biblioteca Nacional de España, Accession number: bdh0000254979.

listed in columns. Angle and arc length of the sectors for each climate zone are specified; no locations are usually listed. The addition of two climate zones in the north represents a rupture with the tradition. The reason for such change, however, was the evident fact that those zones were now inhabited at least as much as the seven defined by the tradition (Fig. S45).

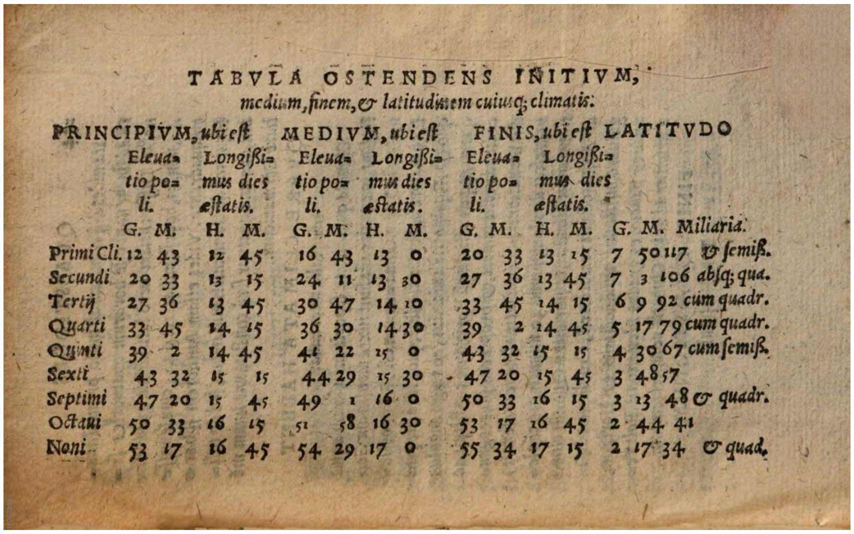

TABVLA OSTENDENS INITIVM, medium, finem, & latitudinem cuiusq; climatis:

	PRINCIPIVM, ubi est				MEDIVM, ubi est				FINIS, ubi est				LATITVDO		
	Eleuatio poli.		Longißimus dies æstatis.		Eleuatio poli.		Longißimus dies æstatis.		Eleuatio poli.		Longißimus dies æstatis.				
	G.	M.	H.	M.	G.	M.	H.	M.	G.	M.	H.	M.	G.	M.	Miliaria.
Primi Cli.	12	43	12	45	16	43	13	0	20	33	13	15	7	50	117 & semiß.
Secundi	20	33	13	15	24	11	13	30	27	36	13	45	7	3	106 absq; qua.
Tertij	27	36	13	45	30	47	14	10	33	45	14	15	6	9	92 cum quadr.
Quarti	33	45	14	15	36	30	14	30	39	2	14	45	5	17	79 cum quadr.
Quinti	39	2	14	45	41	22	15	0	43	32	15	15	4	30	67 cum semiß.
Sexti	43	32	15	15	44	29	15	30	47	20	15	45	3	48	57
Septimi	47	20	15	45	49	1	16	0	50	33	16	15	3	13	48 & quadr.
Octaui	50	33	16	15	51	58	16	30	53	17	16	45	2	44	41
Noni	53	17	16	45	54	29	17	0	55	34	17	15	2	17	34 & quad.

Figure S35: **Nine climate zone table.** From: (*219, p. 283*). Augsburg, Staats- und Stadtbibliothek, Uniform Resource Number: urn:nbn:de:bvb:12-bsb11267368-7.

Twenty-four climate zones. These tables extend the schema of the seven and nine climate zones even further north, usually up to the polar circle where the length of the longest day is exactly 24 hours, resulting in either 24 or 23 (and sometimes even less) climate zones depending on how the extension is concretely carried out. Even though the actual number of climate zones listed in these tables can vary, they are here subsumed under the rubric twenty-four climate zones. The Sacrobosco Collection contains eighty-one such tables. They usually (but not always) stretch over more than one page. Their contents and layouts are even more variable than in the two previous cases. There is a particular variability with respect to where the tables start in the south and how they count the parallels from there. In the traditional schema, the first climate zone has its southern confine where the longest day is 12 hours and 45 minutes, corresponding to a parallel at 12 degrees 45 latitude which was counted as the first parallel. Of course this schema of defining parallels by day-length in hours of the longest day in increments of 15 minutes can be extended further south to the equator, adding three more (including the the equator itself) parallels. Most of the tables in this group indeed make this extension to the

south. The extension is, however, done in different ways which result in different counts of the parallels (Figure S36a and S36b, S37a, and S37b). At times, the first zone is supposed to start at the Earth's equator, with the longest day (like every day there) measuring 12 hours, but sometimes the first zone begins where the longest day measures 12 hours and 15 minutes. Both cases result in 24 climate zones. As retained from the tradition, however, the first zone often starts at day-length 12 hours and 45 minutes, in which case there are only 23 zones. Figure S37c provides an example of an extreme variation. First it only gives the southern confines of the zones listed. Moreover, it starts the first climate zone at the equator, and thus the zones listed do not correspond to the traditional ones (neither in their numbering nor in latitude of their confines). Among other consequences, this results in an actual number of twenty-five climate zones, though we still maintain that it generally belongs to the group of tables showing twenty-four zones.

From a historical point of view, the appearance and spread of the twenty-four zone tables can be considered as the consequence of the recognition that the entire globe, as it was becoming known through the journeys of exploration, was actually inhabited. This interpretation is supported by the fact that we do find one instance where the schema of the climate zones is applied to the southern hemisphere, as testified by a table listing climate zones south of the equator (see Figure S38).

The Spread of Climate Zone Tables

By means of our model, we were able to identify a group of 224 tables (out of about 10,000) that display data related to the climate zones and that can be distinguished into three sub-groups of tables as described above. Furthermore, the closer analysis of this hitherto unexplored historical material allowed us to formulate the hypothesis that departure from the tradition, represented by the tables displaying nine and twenty-four climate zones respectively, is due to the increas-

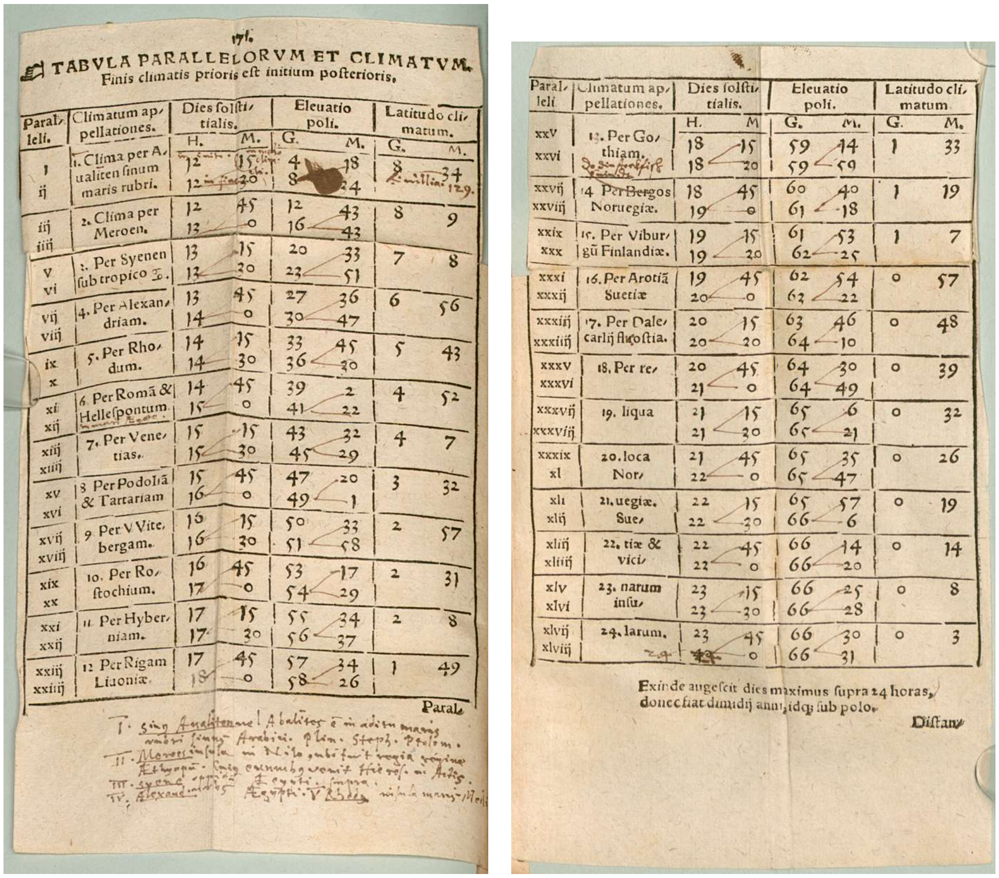

(a) Left page (b) Right page

Figure S36: **Twenty-four climate zone table.** From: (*234, pp. 171r–171v*). München, Bayerische Staatsbibliothek, Uniform Resource Number: urn:nbn:de:bvb:12-bsb00021009-2.

ing recognition that the concept of the inhabitable portion of the Earth's surface was losing its scientific meaning as it faced the discoveries resulting from the journeys of exploration. To try to determine the validity or at least the plausibility of this hypothesis, we analyze the spatio-temporal distribution of these tables by matching the identified tables with the bibliographic metadata of the editions in which they were printed.

First of all, we look at the temporal distribution of the three types of tables. We bin all the editions in the corpus according to publication date for each decade (Fig. S39, lower half). Similarly, for each decade, we plot the number of editions that contain at least one table be-

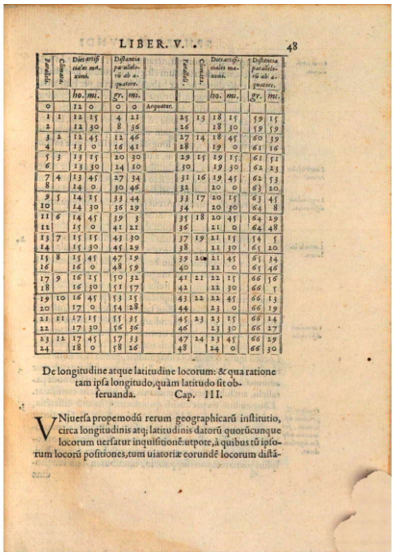

(a) From: (*208, p. 48r*). München, Bayerische Staatsbibliothek, Uniform Resource Number: urn:nbn:de:bvb:12-bsb10198974-9.

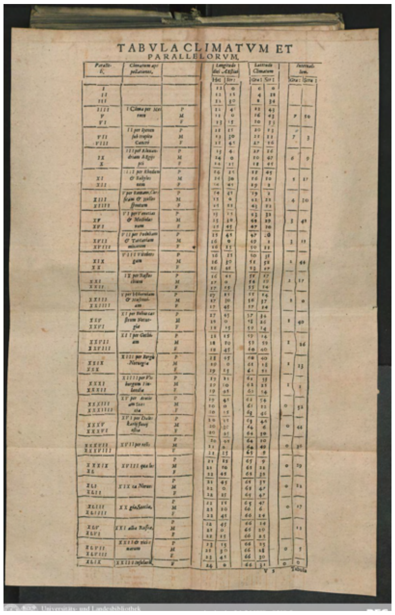

(b) From: (*235, p. 296*). Universitäts- und Landesbibliothek Sachsen-Anhalt, http://dx.doi.org/10.25673/opendata2-7144.

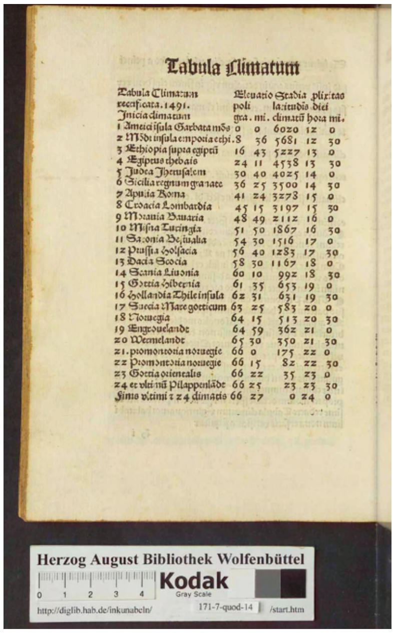

(c) From: (*236, sign. Hi-1*). Herzog August Bibliothek Wolfenbüttel, http://diglib.hab.de/inkunabeln/171-7-quod-14/start.htm.

Figure S37: **Twenty-four climate zone tables.**

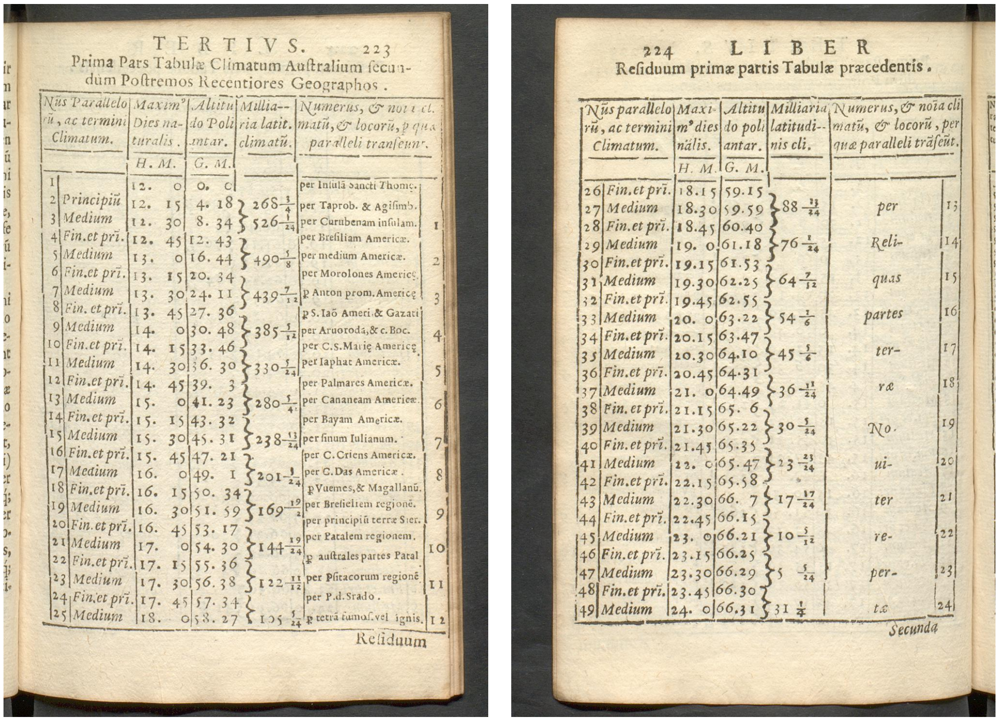

Figure S38: **Twenty-four climate zone table for the southern hemisphere.** From: (*237, pp. 223–224*). ETH-Bibliothek Zürich, https://doi.org/10.3931/e-rara-17609.

longing to one of the three principal types of climate-zones tables that we have identified. We distinguish editions comprising tables for seven, nine, and twenty-four climate zones. We then add one final bin indicating the number of editions published per decade that include both a seven and a twenty-four climate zone table (Fig. S39, upper half).

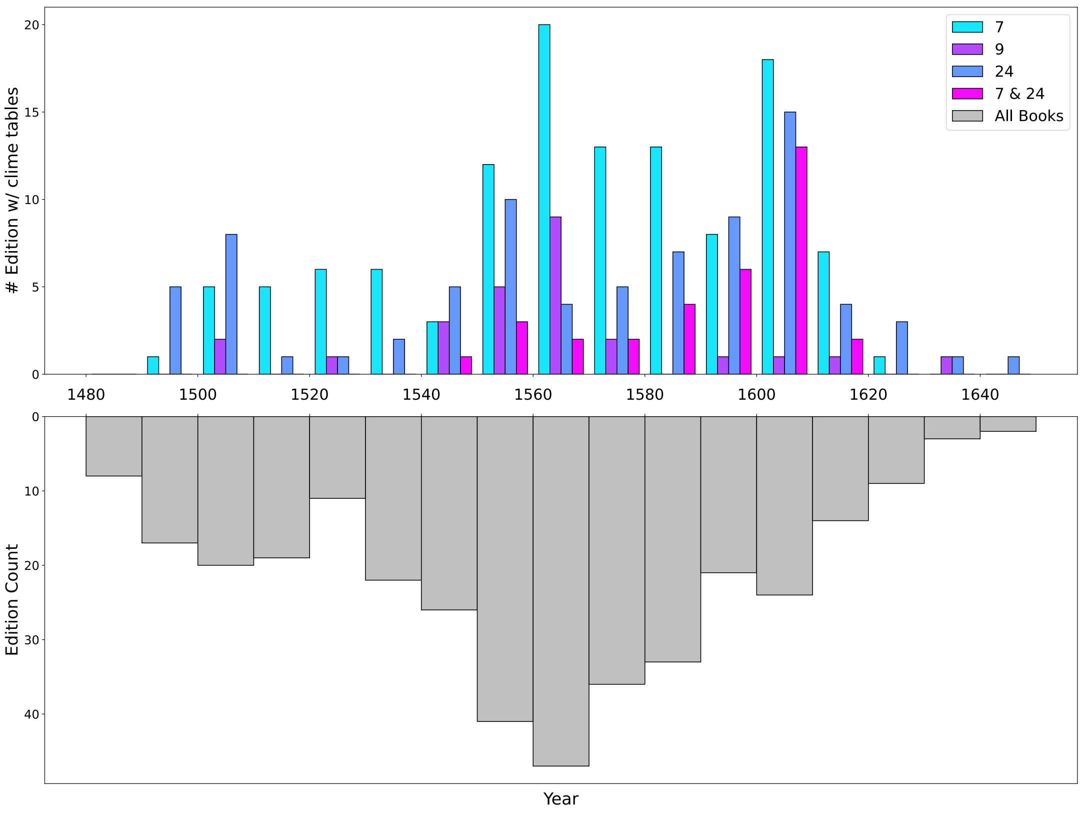

Figure S39: **Temporal spread of print production.** Analysis of the publication patterns of the different types of climate zone tables contained in the corpus.

Contrary to what one might expect at this point, the tradition, represented by the data concerned with the seven climate zones, remains rather robust for the entire period considered, which ends 158 years after the discovery of the "New World" (the American continent). As might be expected, however, the rupture with the tradition represented by the nine climate-zones table spread about 40 years before the twenty-four zones table received the same degree of of attention in print. This chronology supports the historical hypothesis that the emergence of new types of climate zone tables was associated with the increasing knowledge of the Earth's surface and human habitation patterns.

Moreover, if we directly compare the metadata for the editions containing the table displaying the twenty-four climate zones and those containing the traditional seven-zone table a

further interesting aspect is revealed. Firstly, many editions that contain the tables displaying the twenty-four climate zones also contain the traditional seven-zone table. Secondly, the peak in the production of the twenty-four zones table is due to exactly those editions that contain both types of tables (Fig. S39).

Finally, by considering the more abstract subject of the mode of production of scientific knowledge during the early modern period, the present case study allows us to assert that scientific innovations as represented by some of the computational tables in our corpus were introduced and could become successful mainly by building upon traditional and well-accepted knowledge. This same pattern is also observed in the case of the textual apparatus of the treatises where new knowledge is often presented in form of a commentary on a old and authoritative text.

To examine the temporal and spatial dynamic of the geographic spread of the tables, we created and visualized dynamic chains of re-occurrences. Each occurrence of a climate table of a particular type is linked to the first occurrence of a table of the same type that came before it. This simulation enables us to visualize the process of spread of this specific type of table and calculate both the instant and average speed of circulation. Videos of the dynamic visualizations are available in the supplementary materials and their captions in Section B.2 in this Supplement.

By examining the recordings, the first appearance of the tables can be first investigated. All three sorts of tables appear quite early in the collection for the first time, namely either before or just after the turn of the fifteenth century. Both the seven- and nine-zones tables disappear around 1620, while the twenty-four zones table re-occur until the very end of the period considered.

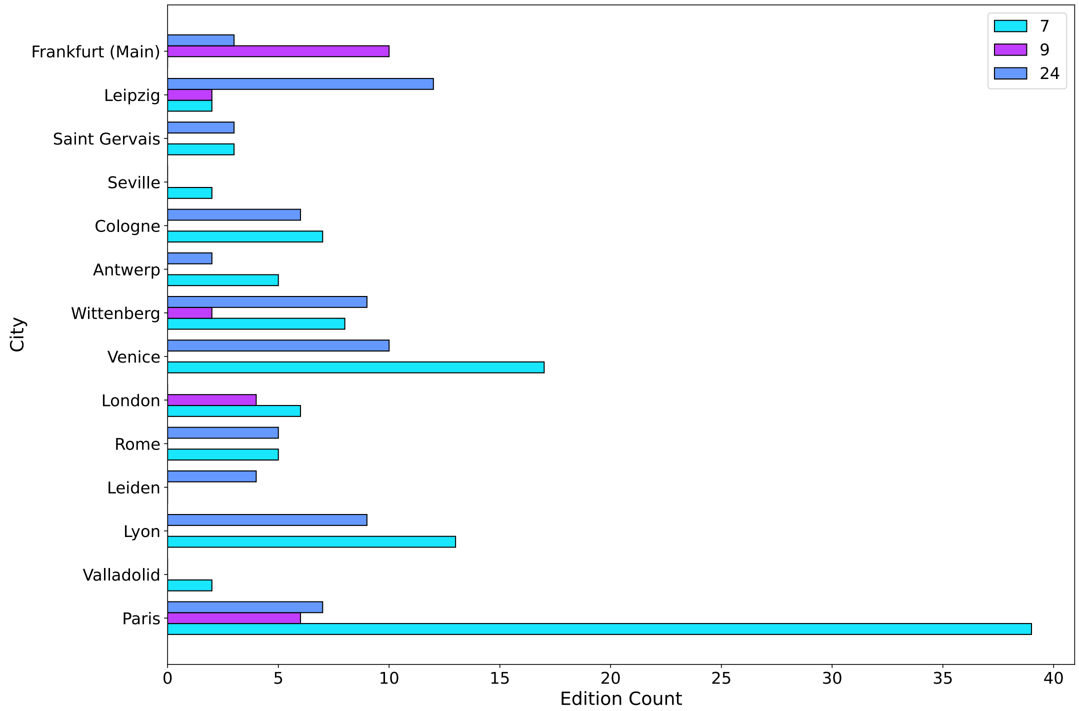

Figure S40: **Centers of book production.** The plot shows the major centers of production of editions that contain one or more of the three kinds of identified climate zone tables.

Observing the spatial distribution of occurrences (Fig. S40, and Figs. S41, S42 and S43), further relevant aspects can be recognized. To improve visibility, centers of production in which only one occurrence of the respective table took place have been suppressed from the plots. The table displaying seven zones is printed in nineteen different cities. In this case, the major centers of production and spread clearly are Paris, Venice, Lyon, and Wittenberg. In the case of the table containing data for nine climate zones, this spread only covers seven different cities, and the major centers are Frankfurt am Main, London, and Paris. Despite their overall lower number of occurrences, the twenty-four climate zone tables, were printed in twenty-two different cities. Their major centers of production were Leipzig, Venice, and Wittenberg.

In particular, we observe first that the geographic spread of the tables for both the seven and the twenty-four climate zones is very similar, though not identical. Both chains of re-

occurrences move from northern Europe toward the Iberian Peninsula and embrace northern Italy and South France. Toward the east, they do not go beyond Wittenberg. In the case of the nine-zones table, however, the dynamic is very different: it remains a fundamentally northern European phenomenon, which does, however, reach as far east as the city of Krakow.

B.1.2.2 Historical Case 2: Tables of Zodiac Signs

As a second historical case, we selected all printed instances of a particular table which gives the positions of the Sun relative to the signs of the zodiac in degrees for each day of the year. This table occurs in varying layouts in our corpus, where the different layouts partition the full table differently. In some cases the entire table is contained on one page, in other books it is distributed over as many as nine pages.

Due to the precession of the earth's axis, the positions of the Sun for a given point of time in the solar year (for instance the vernal equinox) gradually changes over time against the ecliptic. More importantly, before the calendar reform of 1582, the solar year was not in tune with the calendar year, such that the calendar date, for instance of the equinoxes, changed over time. In effect this meant that the position of the Sun against the ecliptic for a given calendar day depends on the year for which it is calculated.

Thanks to our model, we were able to investigate the spread of such table in the about 180 years considered here. First, we immediately recognized that, in our corpus, two variants of the sun-zodiac table are present: first, tables designated as valid for the times of the "ancient" poets (*veterum poetarum temporibus accommodata*) where the Sun is 16 degrees into Capricorn on the first of January and, second, tables valid for "contemporary" times (*nostro tempori*) where the Sun, on the first day of the year, has advanced 5 degrees and is located 21 degrees into Capricorn. Historically, the contemporary table was inserted in the treatises to teach the students the correspondence between the calendar and the celestial phenomena. Such correspondence,

then, could be used to date past events, as long as an observation concerning the position of the Sun was made explicit in the sources. The table for the ancients is in fact called "the table for the ancient poets" because it refers to texts of classical literature by authors such as Hesiod, Ovid, or Pliny.

In antiquity the correspondence between this celestial phenomenon and the calendar was common knowledge and, when they described particular events, ancient authors rarely missed the chance to signal the position of the Sun in the Zodiac, so that other and later readers could reconstruct the date of the event. This subject—the reconstruction of the temporal order of historical events described in ancient sources—became very important during the sixteenth century, especially in the cultural circles of the Protestant reformation. Initially, students could use the contemporary table and, through further calendric computations as well as astronomic calculations concerned with the precession of the equinoxes, they could date events described by the ancient authors. This method eventually proved to be too complex, so the lecturers in Wittenberg introduced a new table already adjusted for the ancient time. In fact, this table is almost always directly accompanied by another table specifically referring to and valid for Alexandria and Rome, the two places commonly referred to by the ancient authors in their observations and descriptions. This table lists for the most prominent stars the degree of the zodiac rising and setting respectively, together with the corresponding star as observed from either of the two locations. Combined with the sun-zodiac table, one can thus effortlessly determine the cosmic risings and settings of the stars for these two locations (and thus eventually also the helical and acronical risings and settings), another type of data often given in the ancient literature. For the historical reasons for the introduction of a new computational table concerned only with the ancient classical time, see (*94*). For examples on how past events were dated, see (*238*).

Essentially, the difference between the sixteenth-century and the ancient table amounts to a shift of the columns listing the days of the year with respect to columns giving the angular

locations. Thus from the perspective of our similarity model, these two variations represent the same (more abstract) table. Examples of the two sorts of tables, taken from the same treatise published in Wittenberg in 1582 are given in Figs. S46a and S46b for the contemporary time and in Figs. S47a and S47b for the ancient classical time. Since the equinoxes drift westward along the ecliptic one degree in about 71 years, a five degree shift would correspond to a time difference of 350 years. Yet in the Julian calendar, valid before the calendar reform of 1582, dates of a fixed solar event like the equinoxes increases by three quarter of a day every century. Using modern parameters, the time difference between the tables then amounts to about 750 years. This value of course depends on the value for the precession, which was debated at the time. Indeed in the Medieval period the prevalent opinion was that the precession was not constant but changed over time, a theory known as trepidation. Ptolemy gives a value of 1/100 degrees *per annum* for the precession (*240*). Using this value, a time difference for the two tables of approximately 2000 years results, which fits quite well with the distinction between the "old poets" and "our times."

The Spread of Zodiac Signs Tables

The identification of Sun-zodiac tables has been greatly facilitated by our approach, which was party informed by previous historical results (*94*). Indeed, without it, the task would be very laborious and almost impossible. Also in this case we can visualize the spread of the tables in Europe during the early modern period.

Videos of a dynamic visualization of the spread of the Sun-Zodiac tables were generated, too. The spread is determined according to time and locations of appearance of editions containing the tables.

The visualization concern solely those editions that contain the tables discussed. This implies, however, that the visualizations do not completely show the spread of this historical phe-

nomenon for two specific reasons. The first concerns the fact that a certain number of treatises discuss the subject at length, but furnish data in the flow of the text and could not therefore be identified by our model. The second relates instead to the preservation history of our historical sources. Especially the table for the ancient time was often printed as a foldout bound at the end of the book. We have several cases in which a manual analysis shows that the foldout once existed but was then later torn away (*94*). The videos of the dynamic visualizations are available in the supplementary materials and their captions in Section B.2 in this Supplement.

In the case of the contemporary table, its spread begins in 1545 in Wittenberg and forty-six different editions could be identified as containing this table. The overall geographic spread remains limited to the German and French speaking regions. In total, only printers and publishers of six different cities printed this table. The two major centers of production were Wittenberg and Paris (Fig. S48, top panel). The table concerning the position of the Sun in the zodiac as observable in ancient classical time has a more limited spread pattern. Only twenty editions in the corpus contain it. The table appears for the first time in 1549 and was produced in only three different locations, with Wittenberg clearly maintaining the primary role. The circulation of the table is a phenomenon clearly restricted to northern Germany (Fig. S48, bottom panel).

B.1.3 Mathematization and Identity

The dataset underlying this research is obtained from the collections provided by the historical research project "The Sphere. Knowledge System Evolution and the Shared Scientific Identity of Europe." As the project's title suggests, its primary goal is to investigate whether and how the transformation and dissemination of scientific knowledge has served as an element shaping European identity during pre-modernity. In doing so we consider the textbooks as technology of memory (*241*).

The two case studies provide results on whose basis an initial affirmative response can be

formulated concerning the identity-shaping role of scientific knowledge in early modern Europe. The first one, concerned with the tables displaying climate zones, has shown how the journeys of exploration and therefore the discovery of an inhabited world beyond Europe and the Ecumene led to the enrichment of an existing knowledge structure, namely the division of the inhabitable zone into climate zones. This implies that the new geographic discoveries were not perceived as contradicting established scientific knowledge. Instead, they were seen as a reason for expanding the knowledge base, and from this viewpoint, they served as a confirmation of such knowledge. This interpretation is supported by the observation that the success of the new table displaying 24 climate zones was largely dependent on the circulation of editions and treatises that contained not only this new table but also the old, classical one displaying the seven climate zones.

The discovery of the New World (American Continent), with its diverse populations, cultures, and traditions, was especially significant in this respect. Despite the brutality that ensued, the ability to incorporate the new discoveries in an established knowledge system must have been perceived as a confirmation tout court of the science that, at that time, was already seen as the result of collaborative efforts that were taking place at a continental level in Europe. Although this incorporation came at the expense of other cultures, it likely played a role in shaping science as a factor in identity formation. This idea appears all the more plausible if it is considered that previously the most relevant identity-shaping factor of Europe had been religion, particularly through the external representation of authority embodied by the Pope and the Holy See of Rome. In the period considered in this research, the unity of Europe could no longer be guaranteed by religion due to the fragmentation of the church and the consequent political and military conflicts. On the basis of our results, we therefore propose the working hypothesis that while encountering "otherness," science began to emerge as a new identity-consolidating cultural aspect.

The second case study further supports this hypothesis. If science plays a role in shaping identity, its roots need to be investigated as well. During the early modern era, these roots were clearly identified with the philosophical and scientific cultures of classic antiquity, particularly those of the Greeks and Romans. It was one of the most important fathers of the Protestant Reformation, Philipp Melanchthon, who in 1531 and 1538 urged the youth to study astronomy, stating that without it, history would merely be a chaotic collection of unordered pieces of information (*93, 94*). Melanchthon published this call in form of an open letter used as preface to an astronomy textbook. This text soon became the most republished text-part of the entire collection examined in this research, circulating widely across Europe regardless of the religion of the countries where the editions containing the text were printed and distributed.

The significance of Melanchthon's call was twofold. On the one hand it underscored the importance of understanding the history of Western civilization, a crucial step in any process of identity formation. On the other, it further contributed to the dissemination of mathematical culture as Melanchthon invoked mathematical astronomy as a primary basis to support history writing. Dating past events by means of astronomic calculations and investigations became a specialized field, creating a self-reinforcing loop. This process not only promoted scientific development but also positioned science as an identity-shaping factor, at least for what concerns the Latin Elite trans-regional community (*98, 37–46*) that was increasingly sharing astronomic knowledge.

Finally, it is worth considering that the processes unveiled by this research—both on corpus level and at the level of case studies—was taking place in what was becoming the most relevant educational institutional setting: the universities. In conclusion, we would like to highlight that this path has paved the way to the formulation of an important new working hypothesis within the realm of the political epistemology of science: the relationship between identity and domination as pivoted around scientific knowledge.

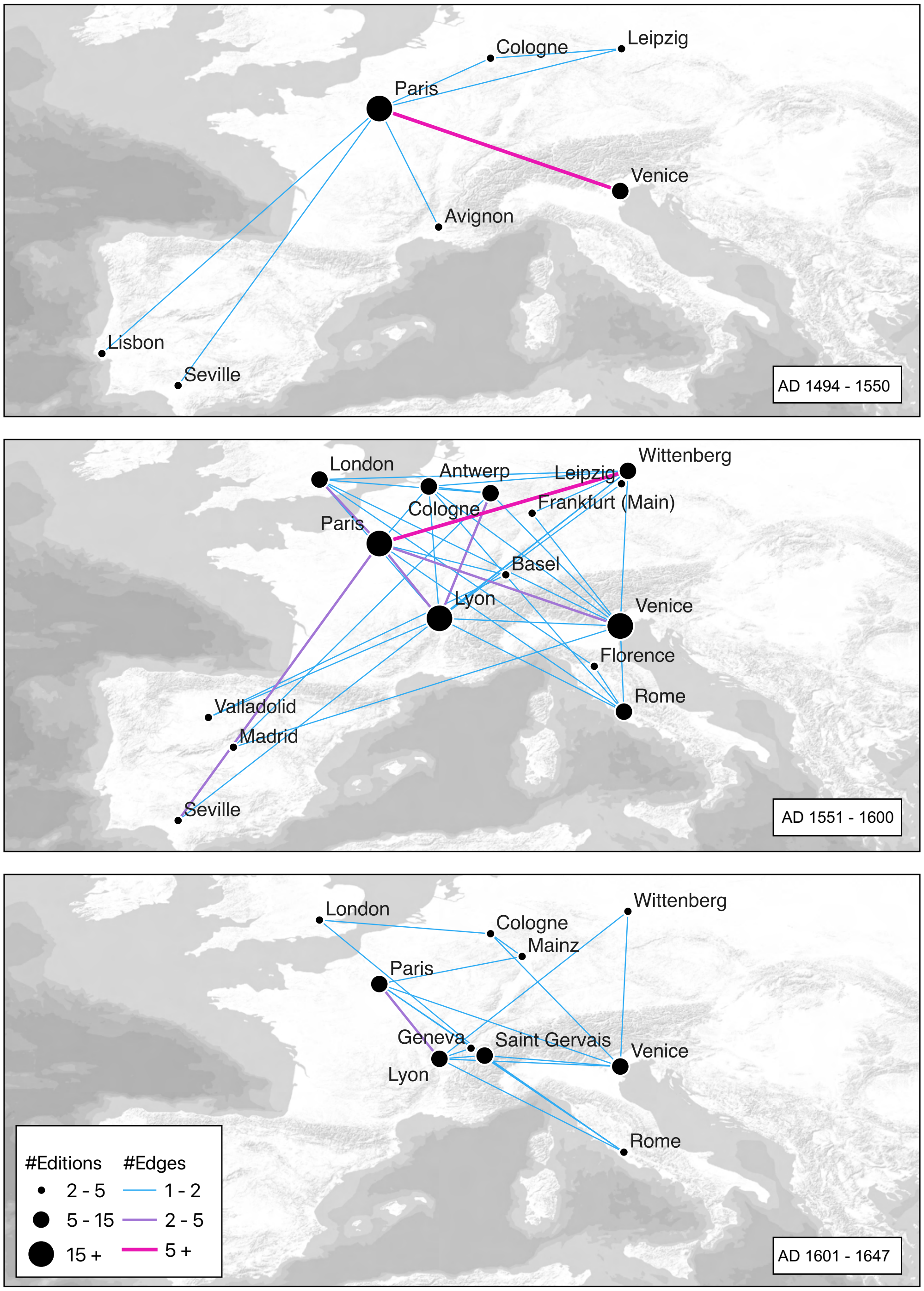

Figure S41: **Spread of seven climate zone table**. Dividing the considered time interval into three roughly equal time windows reveals that the traditional seven climate zone table was spreading across the continent and began to fade during the 17th century.

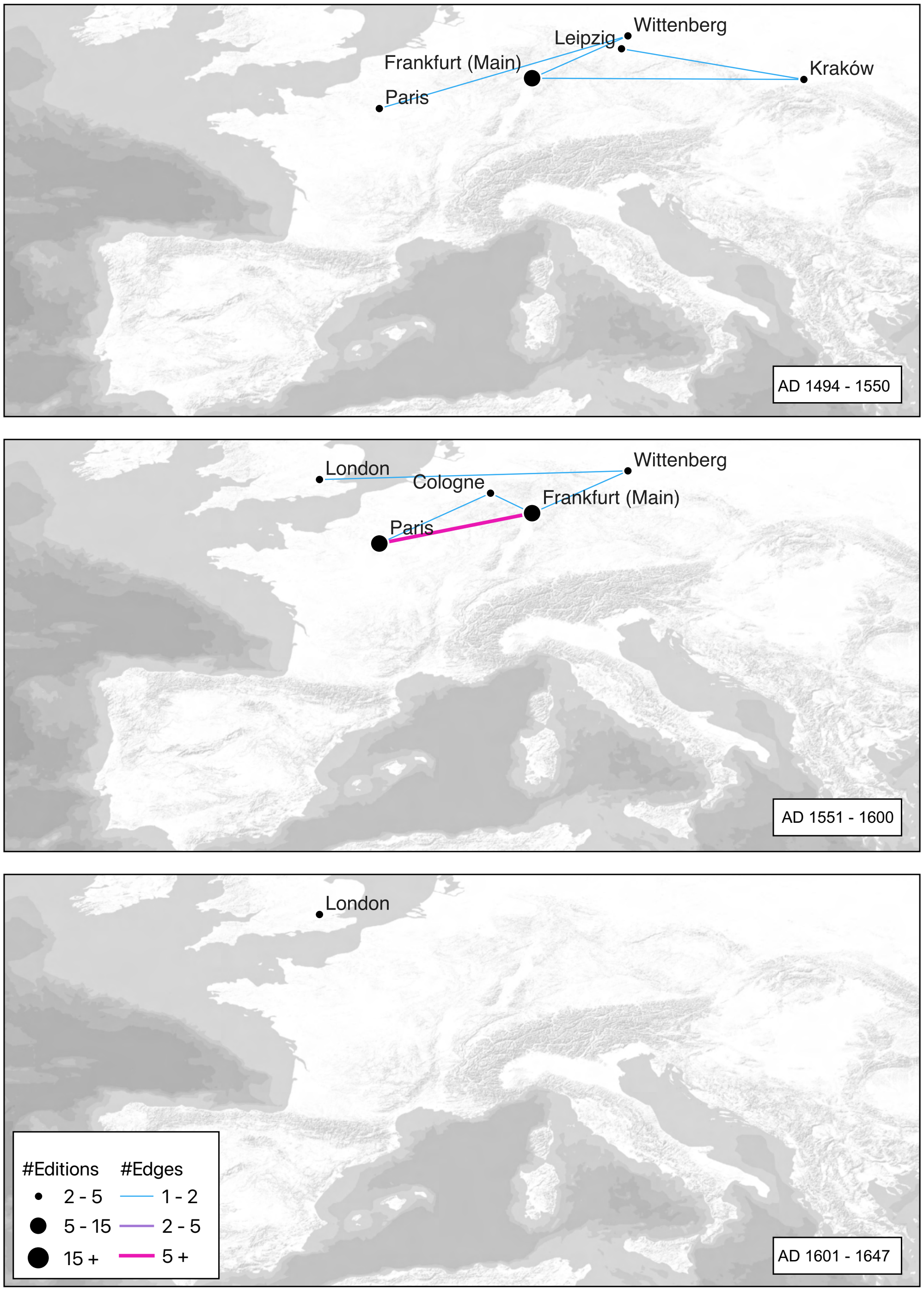

Figure S42: **Spread of the nine climate zone table.** Dividing the considered time interval into three roughly equal time windows reveals that the new nine climate zone table was spreading for a relatively short period and only in Northern Europe.

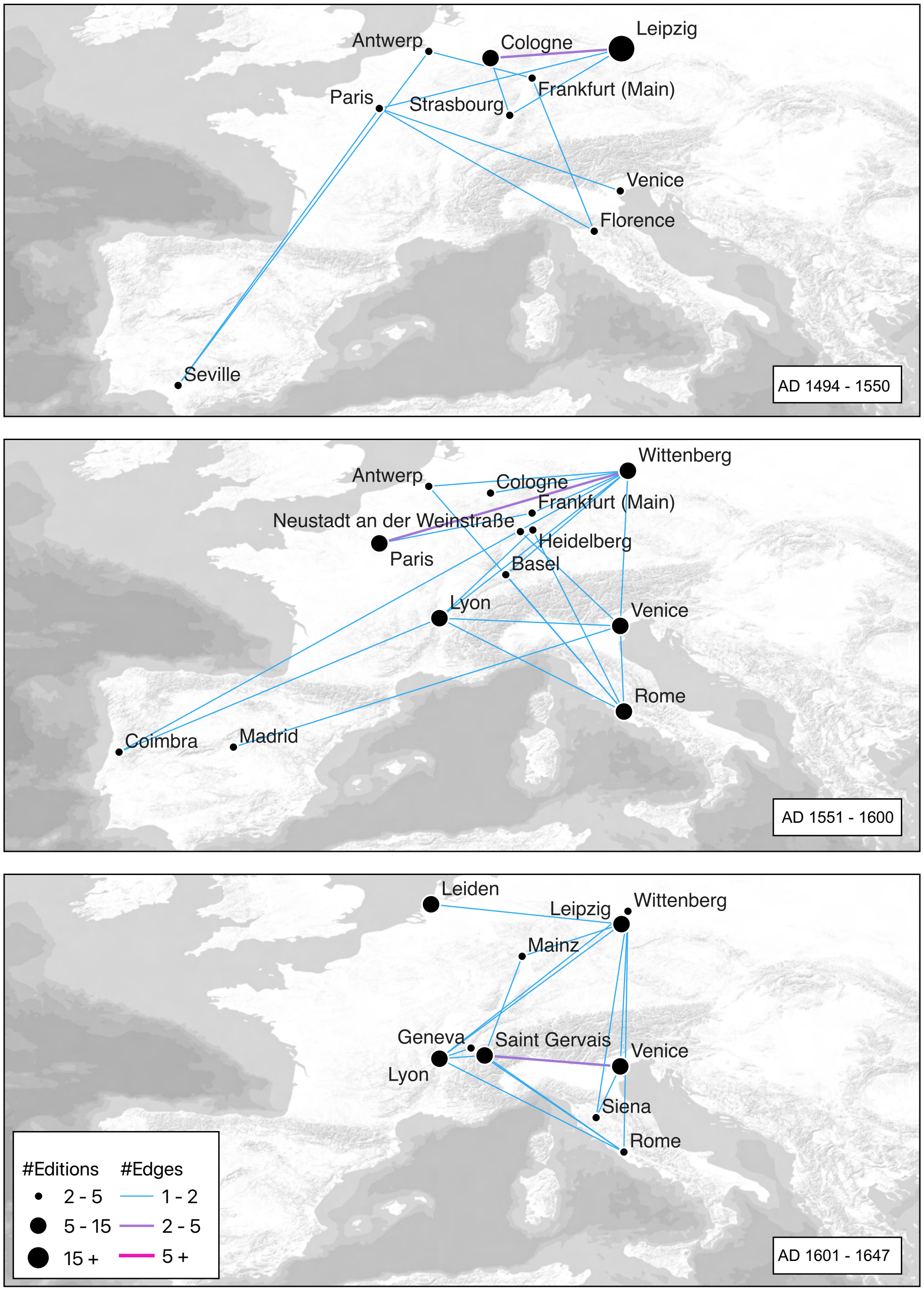

Figure S43: **Spread of the twenty-four climate zone table.** Dividing the considered time interval into three roughly equal time windows reveals that the new twenty-four climate zone table was spreading all over the continent and kept a more dynamic circulation that the one of the seven climate zone table during the 17th century, while the declining phase of the geocentric worldview began becoming evident.

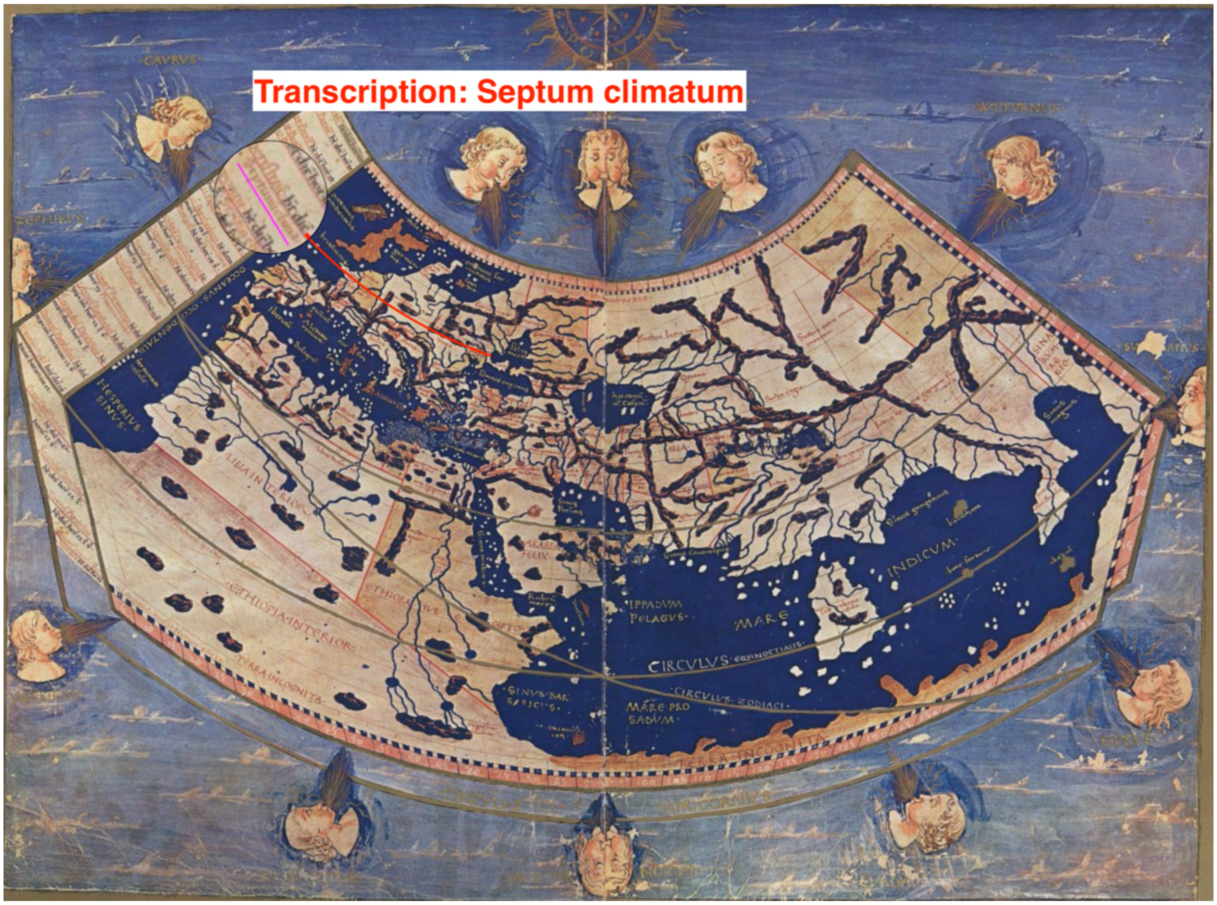

Figure S44: **Ptolemy's world map.** World map, as conceived in the Hellenistic era by Ptolemy, and whose oldest known exemplar was drawn during the 15th century by following Ptolemy's list of coordinates and metric. The 7th climate zone clearly excludes all regions north of Paris, including current Great Britain (the northern border of the seventh climate zone is delineated by a superimposed red line). From: Ptolemy, *Cosmographia*. Map maker: Nicolaus Germanus. From: Ms. membr., lat., sec. XV, cc. I–II, 124, III–IV. 1460–1466. Biblioteca Nazionale di Napoli.

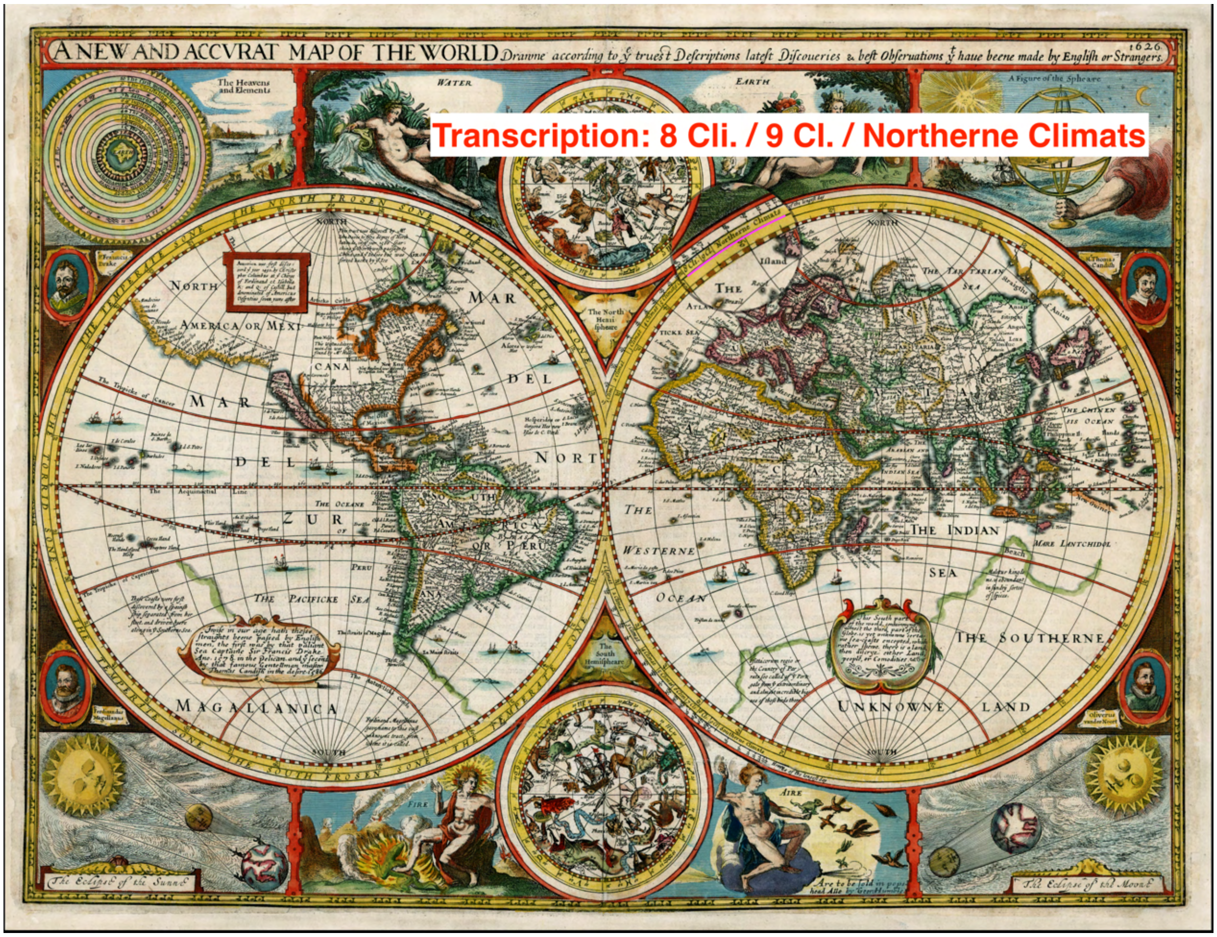

Figure S45: **Robert Walton's world map.** Drawn in 1626, it includes all recently discovered territories on the Earth but considers only nine climate zones as worth explicit mention. The 9th climate zone includes England but was originally introduced to include Wittenberg. Further zones to the north are only generically mentioned (text on the map over the superimposed fuchsia line). From: *A New and Accurat Map of the World Drawne according to ye truest Descriptions latest Discoveries & best observations yt have beene made by English or Strangers*, 1626. London 1627. The Barry Lawrence Ruderman Map Collection. Courtesy Stanford University Libraries. http://purl.stanford.edu/cc815fz9830

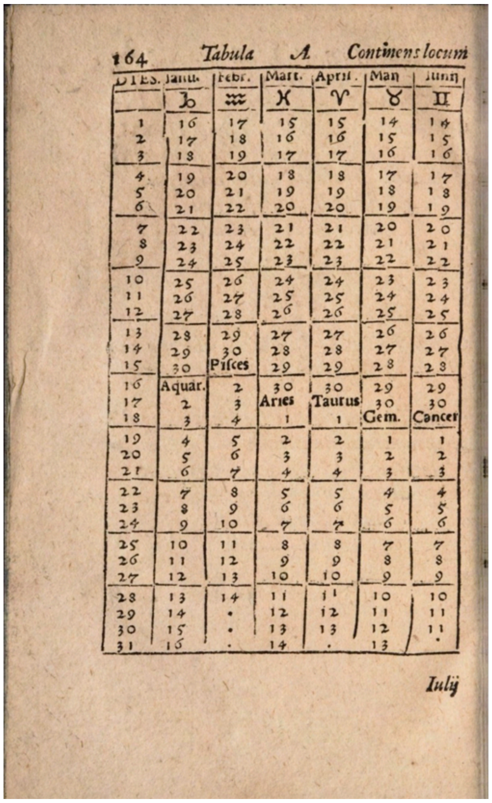

164 *Tabula* A *Continens locum*

DIES.	Janu.	Febr.	Mart.	April.	Maij	Iunij
	♑	♒	♓	♈	♉	♊
1	16	17	15	15	14	14
2	17	18	16	16	15	15
3	18	19	17	17	16	16
4	19	20	18	18	17	17
5	20	21	19	19	18	18
6	21	22	20	20	19	19
7	22	23	21	21	20	20
8	23	24	22	22	21	21
9	24	25	23	23	22	22
10	25	26	24	24	23	23
11	26	27	25	25	24	24
12	27	28	26	26	25	25
13	28	29	27	27	26	26
14	29	30	28	28	27	27
15	30	Pisces	29	29	28	28
16	Aquar.	2	30	30	29	29
17	2	3	Aries	Taurus	30	30
18	3	4	1	1	Gem.	Cancer
19	4	5	2	2	1	1
20	5	6	3	3	2	2
21	6	7	4	4	3	3
22	7	8	5	5	4	4
23	8	9	6	6	5	5
24	9	10	7	7	6	6
25	10	11	8	8	7	7
26	11	12	9	9	8	8
27	12	13	10	10	9	9
28	13	14	11	11	10	10
29	14	.	12	12	11	11
30	15	.	13	13	12	11
31	16	.	14	.	13	.

Iulij

(a) Left page

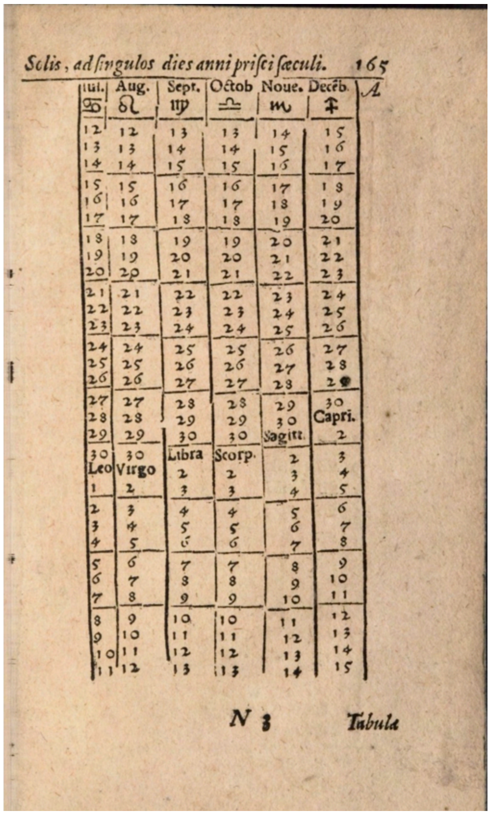

Solis, ad singulos dies anni prisci sæculi. 165

Iul.	Aug.	Sept.	Octob	Noue.	Decēb.
♋	♌	♍	♎	♏	♐
12	12	13	13	14	15
13	13	14	14	15	16
14	14	15	15	16	17
15	15	16	16	17	18
16	16	17	17	18	19
17	17	18	18	19	20
18	18	19	19	20	21
19	19	20	20	21	22
20	20	21	21	22	23
21	21	22	22	23	24
22	22	23	23	24	25
23	23	24	24	25	26
24	24	25	25	26	27
25	25	26	26	27	28
26	26	27	27	28	2[illegible]
27	27	28	28	29	30
28	28	29	29	30	Capri.
29	29	30	30	Sagitt.	2
30	30	Libra	Scorp.	2	3
Leo	Virgo	2	2	3	4
1	2	3	3	4	5
2	3	4	4	5	6
3	4	5	5	6	7
4	5	6	6	7	8
5	6	7	7	8	9
6	7	8	8	9	10
7	8	9	9	10	11
8	9	10	10	11	12
9	10	11	11	12	13
10	11	12	12	13	14
11	12	13	13	14	15

A

N 3 *Tabula*

(b) Right page

Figure S46: ***Nostro tempori*** **Sun-Zodiac table.** The table is computed in relation to the temporal position of the equinoxes in contemporary time (Edition published in 1582). From: (*239, pp. 164–165*). Courtesy of the Library of the Max Planck Institute for the History of Science.

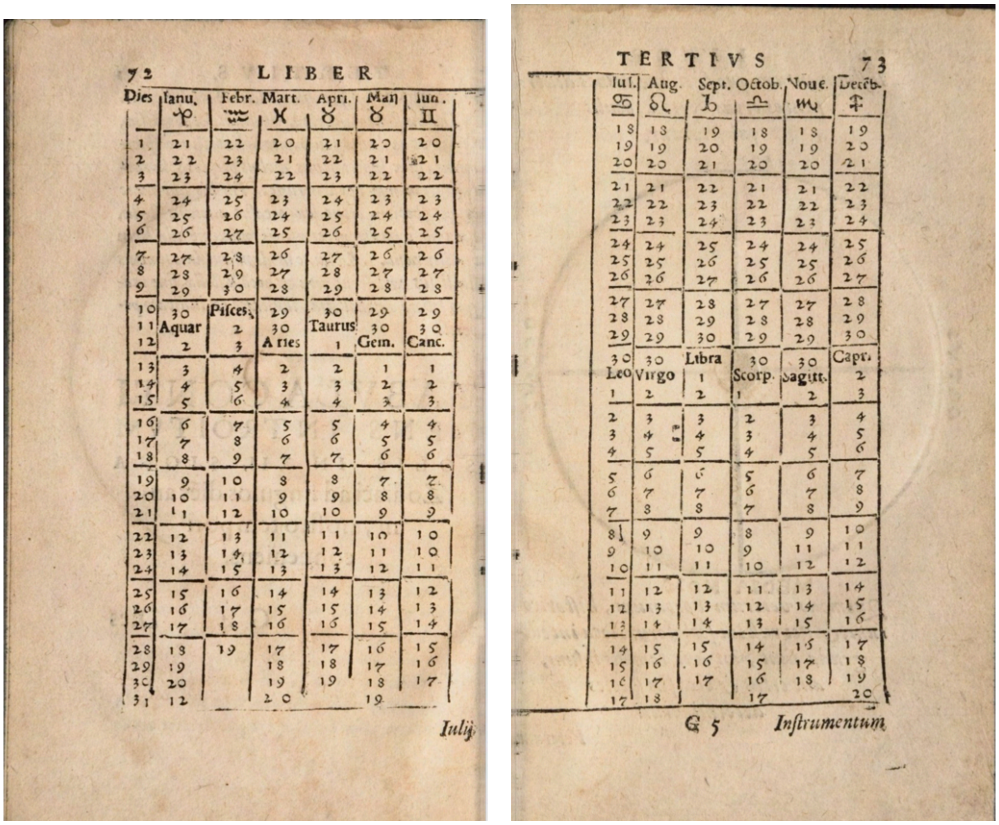

72 LIBER

Dies	Ianu. ♈	Febr. ♒	Mart. ♓	Apri. ♉	Maij ♉	Iun. ♊
1	21	22	20	21	20	20
2	22	23	21	22	21	21
3	23	24	22	23	22	22
4	24	25	23	24	23	23
5	25	26	24	25	24	24
6	26	27	25	26	25	25
7	27	28	26	27	26	26
8	28	29	27	28	27	27
9	29	30	28	29	28	28
10	30	Pisces	29	30	29	29
11	Aquar	2	30	Taurus	30	30
12	2	3	Aries	1	Gem.	Canc.
13	3	4	2	2	1	1
14	4	5	3	3	2	2
15	5	6	4	4	3	3
16	6	7	5	5	4	4
17	7	8	6	6	5	5
18	8	9	7	7	6	6
19	9	10	8	8	7	7
20	10	11	9	9	8	8
21	11	12	10	10	9	9
22	12	13	11	11	10	10
23	13	14	12	12	11	10
24	14	15	13	13	12	11
25	15	16	14	14	13	12
26	16	17	15	15	14	13
27	17	18	16	16	15	14
28	18	19	17	17	16	15
29	19		18	18	17	16
30	20		19	19	18	17
31	12		20		19	

Iulij

TERTIVS 73

Iul. ♋	Aug. ♌	Sept. ♍	Octob. ♎	Noue. ♏	Decemb. ♐
18	18	19	18	18	19
19	19	20	19	19	20
20	20	21	20	20	21
21	21	22	21	21	22
22	22	23	22	22	23
23	23	24	23	23	24
24	24	25	24	24	25
25	25	26	25	25	26
26	26	27	26	26	27
27	27	28	27	27	28
28	28	29	28	28	29
29	29	30	29	29	30
30	30	Libra	30	30	Capr.
Leo	Virgo	1	Scorp.	Sagitt.	2
1	2	2	1	2	3
2	3	3	2	3	4
3	4	4	3	4	5
4	5	5	4	5	6
5	6	6	5	6	7
6	7	7	6	7	8
7	8	8	7	8	9
8	9	9	8	9	10
9	10	10	9	11	11
10	11	11	10	12	12
11	12	12	11	13	14
12	13	13	12	14	15
13	14	14	13	15	16
14	15	15	14	15	17
15	16	16	15	17	18
16	17	17	16	18	19
17	18		17		20

G 5 Instrumentum

(a) Left page (b) Right page

Figure S47: **Sun-Zodiac table *veterum poetarum temporibus accommodata*.** The table is computed in relation to the temporal position of the equinoxes in ancient classical time (Edition published in 1582). From: (*239, pp. 72–73*). Courtesy of the Library of the Max Planck Institute for the History of Science.

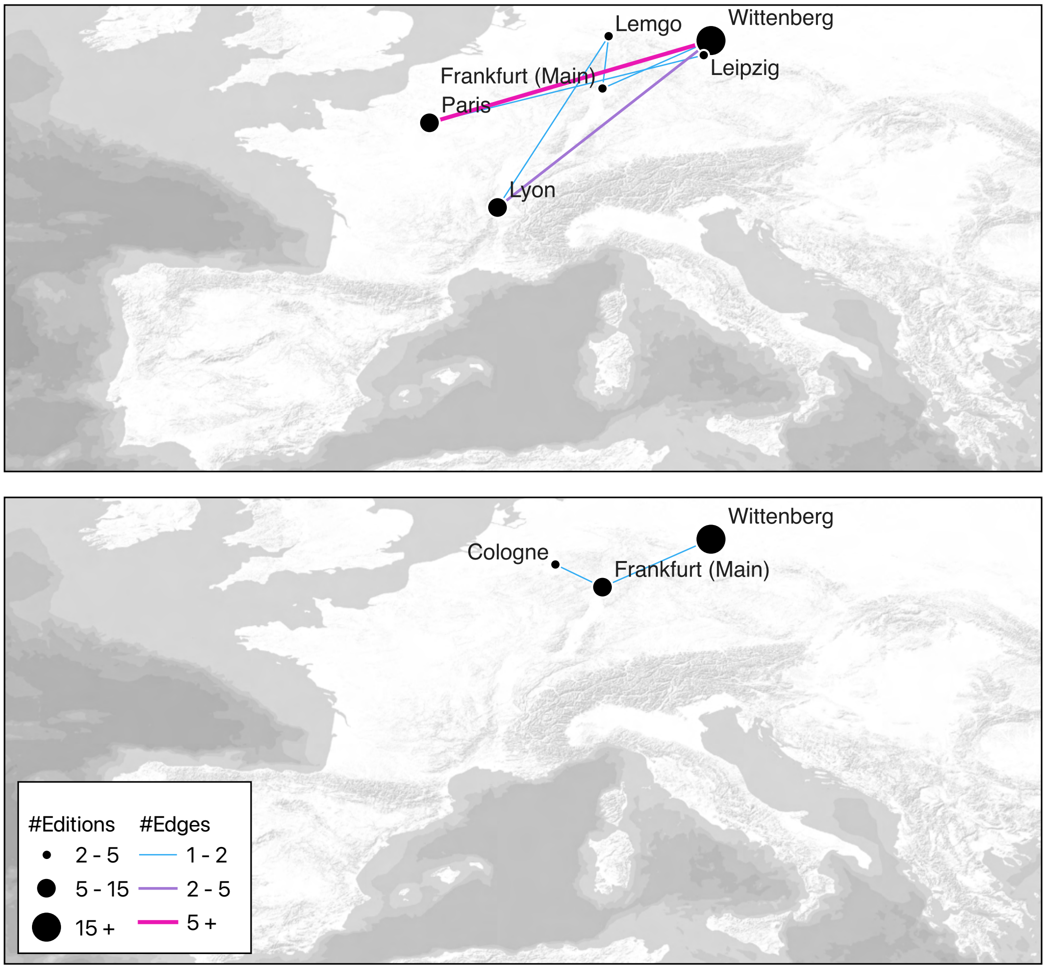

Figure S48: **Sun-Zodiac tables for contemporary and ancient times.** Top: Spread of the contemporary (16th cent.) Sun-Zodiac table showing a prevalent interest in this subject in Northern and Central Europe. Bottom: Spread of the Sun-Zodiac table calculated for the ancient times showing the interest for this subject mainly in German speaking territories.

B.2 Movie Captions

Movie 1. Filename: adj1719_Supplementary Movie_mov1_seq1_v1.mov

Geo-temporal spread of the table displaying seven climate zones. The spread process of this table starts with the appearance of the first tables and continues until ca. 1620. The table was printed in nineteen different cities. Among them, the major centers of production and spread clearly are Paris, Venice, Lyon, and Wittenberg.

Movie 2. Filename: adj1719_Supplementary Movie_mov2_seq2_v1.mov

Geo-temporal spread of the table displaying nine climate zones. The spread process of this table starts at the beginning of the 16th century and continues until ca. 1620. The spread of this table concerns only seven different cities, among them, Frankfurt am Main, London, and Paris.

Movie 3. Filename: adj1719_Supplementary Movie_mov3_seq3_v1.mov

Geo-temporal spread of the table displaying twenty-four climate zones. The spread process of this table starts at the end of the 15th century and continues until the end of the period considered it, as expected, after the end of the process of diffusion of the alternatives climate tables. The spread of this table concerns twenty-four locations, among them the major centers of production were Leipzig, Venice, and Wittenberg.

Movie 4. Filename: adj1719_Supplementary Movie_mov4_seq4_v1.mov

Geo-temporal spread of the 16th century table displaying the contemporary (*nostro*) Sun-Zodiac table. Its spread begins in 1545 in Wittenberg and forty-six different editions could be identified as containing this table. The overall geographic spread remains limited to the German and French speaking regions. In total, only printers and publishers of six different cities printed this table. The two major centers of production were Wittenberg and Paris.

Movie 5. Filename: adj1719_Supplementary Movie_mov5_seq5_v1.mov

Geo-temporal spread of the table displaying the Sun-Zodiac table valid for ancient writers (*veterum*). The spread pattern appears limited. Only twenty editions in the corpus contain it. The table appears for the first time in 1549 and was produced in only three different locations, with Wittenberg clearly maintaining the primary role. The circulation of the table is a phenomenon clearly restricted to northern Germany.

REFERENCES AND NOTES

1. A. Koyré, *Études galiléennes* (Hermann, 1939).
2. A. Koyré, *The Astronomical Revolution. Copernicus, Kepler, Borelli* (Cornell Univ. Press, 1973).
3. A. Koyré, *From the Closed World to the Infinite Universe* (Johns Hopkins Press, 1957).
4. O. Perdersen, *Early Physics and Astronomy: A Historical Introduction* (Cambridge Univ. Press, 1993).
5. R. Westfall, *The Construction of Modern Science* (John Wiley and Sons, 1971).
6. F. H. Cohen, *The Rise of Modern Science Explained: A Comparative History* (Cambridge Univ. Press, 2016).
7. T. S. Kuhn, *The Copernican Revolution* (Harvard Univ. Press, 1957).
8. P. Bussotti, B. Lotti, *Cosmology in the Early Modern Age: A Web of Ideas* (Springer, 2022).
9. W. R. Laird, *The Renaissance of Mechanics. Ancient Science in the Age of Humanism* (Springer, 2024).
10. T. S. Kuhn, *The Structure of Scientific Revolutions* (University of Chicago Press, 1962).
11. F. Braudel, *La méditerranée et le monde méditerranéen à l'époque de Philippe II* (Armand Colin, 1949).
12. E. Bloch, *Erbschaft dieser Zeit* (Oprecht & Helbing, 1935).
13. E. Bloch, *Tübinger Einleitung in die Philosophie I* (Suhrkamp Verlag, 1963).
14. P. Burke, *What Is the History of Knowledge?* (Polity Press, 2015).
15. L. Daston, The history of science and the history of knowledge. *KNOW: A Journal on the Formation of Knowledge* (University of Chicago, 2017), vol. 1.

16. J. Östling, D. L. Heidenblad, Fulfilling the promise of the history of knowledge: Key approaches for the 2020s. *Journal for the History of Knowledge* (2020), vol. 1.

17. Y. LeCun, Y. Bengio, G. Hinton, Deep learning. *Nature* **521**, 436–444 (2015).

18. S. Hochreiter, J. Schmidhuber, Long short-term memory. *Neural Comput.* **9**, 1735–1780 (1997).

19. J. Schmidhuber, Deep learning in neural networks: An overview. *Neural Netw.* **61**, 85–117 (2015).

20. A. Radford, J. Wu, R. Child, D. Luan, D. Amodei, I. Sutskever, Language models are unsupervised multitask learners (Tech. Rep. OpenAI, 2019).

21. J. Redmon, S. Divvala, R. Girshick, A. Farhadi, You only look once: Unified, real-time object detection, in *2016 IEEE Conference on Computer Vision and Pattern Recognition (CVPR)* (IEEE, 2016), pp. 779–788.

22. K. He, X. Zhang, S. Ren, J. Sun, Deep residual learning for image recognition, in *Proceedings of the IEEE Conference on Computer Vision and Pattern Recognition (CVPR)* (IEEE, 2016).

23. J. Devlin, M.-W. Chang, K. Lee, K. Toutanova, BERT: Pre-training of deep bidirectional transformers for language understanding, in *Proceedings of the 2019 Conference of the North American Chapter of the Association for Computational Linguistics: Human Language Technologies, Volume 1 (Long and Short Papers)* (Association for Computational Linguistics, 2019), pp. 4171–4186.

24. T. B. Brown, B. Mann, N. Ryder, M. Subbiah, J. Kaplan, P. Dhariwal, A. Neelakantan, P. Shyam, G. Sastry, A. Askell, S. Agarwal, A. Herbert-Voss, G. Krueger, T. Henighan, R. Child, A. Ramesh, D. M. Ziegler, J. Wu, C. Winter, C. Hesse, M. Chen, E. Sigler, M. Litwin, S. Gray, B. Chess, J. Clark, C. Berner, S. McCandlish, A. Radford, I. Sutskever, D. Amodei, Language models are few-shot learners. arXiv:2005.14165 [cs.CL] (2020).

25. A. D. Cohen, A. Roberts, A. Molina, A. Butryna, A. Jin, A. Kulshreshtha, B. Hutchinson, B. Zevenbergen, B. H. Aguera-Arcas, C. ching Chang, C. Cui, C. Du, D. D. F. Adiwardana, D. Chen, D. D. Lepikhin, E. H. Chi, E. Hoffman-John, H.-T. Cheng, H. Lee, I. Krivokon, J. Qin, J. Hall, J. Fenton, J. Soraker, K. Meier-Hellstern, K. Olson, L. M. Aroyo, M. P. Bosma, M. J. Pickett, M. A. Menegali, M. Croak, M. Díaz, M. Lamm, M. Krikun, M. R. Morris, N. Shazeer, Q. V. Le, R. Bernstein, R. Rajakumar, R. Kurzweil, R. Thoppilan, S. Zheng, T. Bos, T. Duke, T. Doshi, V. Y. Zhao, V. Prabhakaran, W. Rusch, Y. Li, Y. Huang, Y. Zhou, Y. Xu, Z. Chen, Lamda: Language models for dialog applications. arXiv:2201.08239 [cs.CL] (2022).

26. C. Papadopoulos, S. Pletschacher, C. Clausner, A. Antonacopoulos, The impact dataset of historical document images, in *Proceedings of the 2nd International Workshop on Historical Document Imaging and Processing, HIP '13* (Association for Computing Machinery, 2013), p. 123–130.

27. A. Fischer, "IAM-HistDB: A dataset of handwritten historical documents" in *Handwritten Historical Document Analysis, Recognition, and Retrieval - State of the Art and Future Trends* (World Scientific, 2020), pp. 11–23.

28. K. Nikolaidou, M. Sueret, H. Mojayet, M. Liwicki, A survey of historical document image datasets. *Int. J. Document Anal. Recognit.* **25**, 305–338 (2022).

29. J. Büttner, J. Martinetz, H. El-Hajj, M. Valleriani, Cordeep and the sacrobosco dataset: Detection of visual elements in historical documents. *J. Imaging* **8**, 285 (2022).

30. G. Graßhoff, M. Y. Abkenar, Kepler's astronomia nova–A challenge for computational history and the philosophy of science, in *Applied and Computational Historical Astronomy. Angewandte und computergestützte historische Astronomie: Proceedings of the Splinter Meeting in the Astronomische Gesellschaft, Sept. 25, 2020. Nuncius HamburgensisBeiträge zur Geschichte der Naturwissenschaften* (Tredition, 2021), vol. 55.

31. O. Ronneberger, P. Fischer, T. Brox, "U-net: Convolutional networks for biomedical image segmentation" in *Medical Image Computing and Computer-Assisted Intervention–MICCAI 2015: 18th International Conference, Munich, Germany, October 5–9, 2015, Proceedings, Part III* (Springer International Publishing, 2015), pp. 234–241.

32. A. Radford, J. W. Kim, C. Hallacy, A. Ramesh, G. Goh, S. Agarwal, G. Sastry, A. Askell, P. Mishkin, J. Clark, G. Krueger, I. Sutskever, Learning transferable visual models from natural language supervision, ICML, in *Proceedings of Machine Learning Research* (PMLR, 2021), vol. 139, pp. 8748–8763.

33. T. Monnier, M. Aubry, docExtractor: An off-the-shelf historical document element extraction, in *2020 17th International Conference on Frontiers in Handwriting Recognition (ICFHR)* (IEEE Xplore, 2020), pp. 91–96.

34. A. Dutta, G. Bergel, A. Zisserman, Visual analysis of chapbooks printed in scotland, *The 6th International Workshop on Historical Document Imaging and Processing, HIP '21* (Association for Computing Machinery, 2021), pp. 67–72.

35. T. Smits, M. Wevers, A multimodal turn in Digital Humanities. Using contrastive machine learning models to explore, enrich, and analyze digital visual historical collections. *Digit. Scholarsh. Hum.* **38**, 1267–1280 (2023).

36. L. Tsochatzidis, S. Symeonidis, A. Papazoglou, I. Pratikakis, HTR for Greek historical handwritten documents. *J. Imaging* **7**, 260 (2021).

37. C. Wick, J. Zöllner, T. Grüning, Transformer for handwritten text recognition using bidirectional post-decoding, *Document Analysis and Recognition–ICDAR 2021*, J. Lladós, D. Lopresti, S. Uchida, Eds. (Springer International Publishing, 2021), pp. 112–126.

38. M. Li, T. Lv, J. Chen, L. Cui, Y. Lu, D. A. F. Florêncio, C. Zhang, Z. Li, F. Wei, Trocr: Transformer-based optical character recognition with pre-trained models, in *Proceedings of the 37th AAAI Conference on Artificial Intelligence* (AAAI Press, 2023), pp. 13094–13102.

39. P. Ströbel, S. Clematide, T. Hodel, M. Volk, Transformer-based htr for historical documents, in *Workshop on Computational Methods in the Humanities 2022* (CEUR Workshop Proceedings, 2022).

40. Y. Assael, T. Sommerschield, J. Prag, Restoring ancient text using deep learning: A case study on Greek epigraphy, in *Proceedings of the 2019 Conference on Empirical Methods in Natural*

Language Processing and the 9th International Joint Conference on Natural Language Processing (EMNLP-IJCNLP) (Association for Computational Linguistics, 2019), pp. 6368–6375.

41. Y. Assael, T. Sommerschield, B. Schillingford, M. Bodbar, J. Pavlopoulos, M. Chatzipanangiotou, I. Androutsopulos, J. Prag, N. de Freitas, Restoring and attributing ancient texts using deep neural networks. *Nature* **603**, 280–283 (2022).

42. D. Bamman, P. J. Burns, Latin BERT: A contextual language model for classical philology. arXiv:2009.10053 [cs.CL] (2020).

43. E. Fetaya, Y. Lifshitz, E. Aaron, S. Gordin, Restoration of fragmentary babylonian texts using recurrent neural networks. *Proc. Natl. Acad. Sci. U.S.A.* **117**, 22743–22751 (2020).

44. A. Barucci, C. Cucci, M. Franci, M. Loschiavo, F. Argenti, A deep learning approach to ancient egyptian hieroglyphs classification. *IEEE Access* **9**, 123438–123447 (2021).

45. D. Baehrens, T. Schroeter, S. Harmeling, M. Kawanabe, K. Hansen, K.-R. Müller, How to explain individual classification decisions. *J. Mach. Learn. Res.* **11**, 1803–1831 (2010).

46. G. Montavon, W. Samek, K.-R. Müller, Methods for interpreting and understanding deep neural networks. *Digit. Signal Process.* **73**, 1–15 (2018).

47. D. Gunning, DARPA's explainable artificial intelligence (XAI) program, in *Proceedings of the 24th International Conference on Intelligent User Interfaces*, *IUI '19* (Association for Computing Machinery, 2019), p. ii.

48. A. B. Arrieta, N. D. Rodríguez, J. D. Ser, A. Bennetot, S. Tabik, A. Barbado, S. García, S. Gil-Lopez, D. Molina, R. Benjamins, R. Chatila, F. Herrera, Explainable artificial intelligence (XAI): Concepts, taxonomies, opportunities and challenges toward responsible AI. *Inf. Fusion* **58**, 82–115 (2020).

49. W. Samek, G. Montavon, S. Lapuschkin, C. J. Anders, K.-R. Müller, Explaining deep neural networks and beyond: A review of methods and applications. *Proc. IEEE* **109**, 247–278 (2021).

50. C. Zednik, H. Boelsen, Scientific exploration and explainable artificial intelligence. *Minds Mach.* **32**, 219–239 (2022).

51. L. M. Pawlowicz, C. E. Downum, Applications of deep learning to decorated ceramic typology and classification: A case study using tusayan white ware from northeast arizona. *J. Archaeol. Sci.* **130**, 105375 (2021).

52. P. Bell, F. Offert, Reflections on connoisseurship and computer vision. *J. Art Hist.* **24**, 1–10 (2021).

53. H. El-Hajj, O. Eberle, A. Merklein, A. Siebold, N. Shlomi, J. Büttner, J. Martinetz, K.-R. Müller, G. Montavon, M. Valleriani, Explainability and transparency in the realm of digital humanities: Toward a historian xai. *Int. J. Digit Hum.* **5**, 299–331 (2023).

54. S. Cole, The hierarchy of the sciences? *Am. J. Soc.* **89**, 111–139 (1983).

55. A. Lundgren, B. Bensaude-Vincent, Eds., *Communicating Chemistry. Textbooks and Their Audiences, 1789–1939* (Science History Publications, 2000).

56. M. Vicedo, Introduction: The secret lives of textbooks. *Isis* **103**, 83–87 (2012).

57. M. Valleriani, Ed., *De sphaera of Johannes de Sacrobosco in the Early Modern Period: The Authors of the Commentaries* (Springer, 2020).

58. M. Valleriani, A. Ottone, Eds., "Publishing Sacrobosco's «De sphaera»" in *Early Modern Europe. Modes of Material and Scientific Exchange* (Springer Nature, 2022).

59. M. Valleriani, F. Kräutli, M. Zamani, A. Tejedor, C. Sander, M. Vogl, S. Bertram, G. Funke, H. Kantz, The emergence of epistemic communities in the sphaera corpus: Mechanisms of knowledge evolution. *J. Hist. Netw. Res.* **3**, 50–91 (2019).

60. M. Zamani, A. Tejedor, M. Vogl, F. Kräutli, M. Valleriani, H. Kantz, Evolution and transformation of early modern cosmological knowledge: A network study. *Sci. Rep.* **10**, 19822 (2020).

61. M. Zamani, H. El-Hajj, M. Vogl, H. Kantz, M. Valleriani, A mathematical model for the process of accumulation of scientific knowledge in the early modern period. *Nature* **10**, 533 (2023).

62. M. Valleriani, "Prolegomena to the study of early modern commentators on Johannes de Sacrobosco's tractatus de sphaera" in *De sphaera of Johannes de Sacrobosco in the Early Modern Period: The Authors of the Commentaries*, M. Valleriani, Ed. (Springer, 2019), pp. 1–23.

63. O. Gingerich, Sacrobosco as a textbook. *J. History Astron.* **19**, 269–273 (1988).

64. O. Gingerich, "Five centuries of astronomical textbooks and their role in teaching" in *The Teaching of Astronomy*, J. M. Pasachoff, J. R. Percy, Eds. (Cambridge Univ. Press, 1990), pp. 189–211.

65. J. Chábas, B. R. Goldstein, *The Alfonsine Tables of Toledo* (Springer, 2003).

66. J. Chabás, B. R. Goldstein, *A Survey of European Astronomical Tables in the Late Middle Ages* (Brill, 2012).

67. B. Gilbert, *The Art of the Woodcut in the Italian Renaissance Book* (The Grolier Club, 1995).

68. E. L. Eisenstein, *The Printing Revolution in Early Modern Europe* (Cambridge Univ. Press, 1996).

69. I. Maclean, *Learning and the Market Place: Essays in the History of the Early Modern Book* (Brill, 2009).

70. A. Nuovo, *The Book Trade in the Italian Renaissance* (Brill, 2013).

71. W. A. Qader, M. M. Ameen, B. I. Ahmed, An overview of bag of words; importance, implementation, applications, and challenges, *2019 International Engineering Conference (IEC)* (IEEE Xplore, 2019), pp. 200–204.

72. N. Dalal, B. Triggs, Histograms of oriented gradients for human detection, in *2005 IEEE Computer Society Conference on Computer Vision and Pattern Recognition (CVPR'05)* (IEEE, 2005), vol. 1, pp. 886–893.

73. E. Nowak, F. Jurie, B. Triggs, Sampling strategies for bag-of-features image classification, ECCV (4) (Springer, 2006), vol. 3954 of *Lecture Notes in Computer Science*, pp. 490–503.

74. J. Yang, Y.-G. Jiang, A. G. Hauptmann, C.-W. Ngo, Evaluating bag-of-visual-words representations in scene classification, in *Proceedings of the International Workshop on Workshop on Multimedia Information Retrieval* (Association for Computing Machinery, 2007), pp. 197–206.

75. S. Bach, A. Binder, G. Montavon, F. Klauschen, K.-R. Müller, W. Samek, On pixel-wise explanations for non-linear classifier decisions by layer-wise relevance propagation. *PLOS ONE* **10**, e0130140 (2015).

76. O. Eberle, J. Büttner, F. Kräutli, K.-R. Müller, M. Valleriani, G. Montavon, Building and interpreting deep similarity models. *IEEE Trans. Pattern Anal. Mach. Intell.* **44**, 1149–1161 (2022).

77. P. F. Grendler, "The «sphaera» in the jesuit education" in *Publishing Sacrobosco's «De sphaera» in Early Modern Europe. Modes of Material and Scientific Exchange*, M. Valleriani, A. Ottone, Eds. (Springer Nature, 2022), pp. 369–406.

78. M. Valleriani, A. Ottone, "Printers, publishers, and sellers: Actors in the process of consolidation of epistemic communities in the early modern academic world" in *Publishing Sacrobosco's «De sphaera» in Early Modern Europe. Modes of Material and Scientific Exchange*, M. Valleriani, A. Ottone, Eds. (Springer Nature, 2022), pp. 1–24.

79. I. Maclean, "Sacrobosco at the book fairs, 1576–1624: The pedagogical marketplace", in *Publishing Sacrobosco's «De sphaera» in Early Modern Europe. Modes of Material and Scientific Exchange*, M. Valleriani, A. Ottone, Eds. (Springer Nature, 2022), pp. 195–232.

80. M. Valleriani, M. Vogl, H. El-Hajj, K. Pham, The network of early modern printers and its impact on the evolution of scientific knowledge: Automatic detection of awareness relations. *Dent. Hist.* **2**, 466–503 (2022).

81. A. Axworthy, "Oronce fine and sacrobosco: From the edition of the Tractatus de sphaera (1516) to the cosmographia (1532)" in *De sphaera of Johannes de Sacrobosco in the Early Modern Period: The Authors of the Commentaries*, M. Valleriani, Ed. (Springer Nature, 2020), pp. 185–264.

82. S. Limbach, "Scholars, printers, and the sphere: New evidence for the challenging production of academic books in wittenberg, 1531–1550" in *Publishing Sacrobosco's «De sphaera» in Early Modern Europe. Modes of Material and Scientific Exchange*, M. Valleriani, A. Ottone, Eds. (Springer Nature, 2022), pp. 155–194.

83. C. Domtera-Schleichardt, *Die Wittenberger »Scripta publice proposita« (1540–1569). Universitätsbekanntmachungen im Umfeld des späten Melanchthon* (Evangelische Verlagsanstalt, 2021).

84. C. D. Jackson, Educational reforms of Wittenberg and their faithfulness to Martin Luther's thought. *J. Christ. Educ.* **10**, 71–87 (2013).

85. R. L. Kremer, "Incunable almanacs and practica as practical knowledge produced in trading zones" in *The Structures of Practical Knowledge* (Springer, 2017), pp. 333–369.

86. H. Leitão, "Um mundo novo e uma nova ciência" in *360° Ciência Descoberta, Catálogo da Exposição* (Fundação Calouste Gulbenkian, 2013), pp. 16–39.

87. P. Burke, *The Renaissance Sense of the Past* (Edward Arnold, 1969).

88. S. Tanaka, *History without Chronology* (Lever Press, 2019).

89. K. Reich, E. Knobloch, Melanchthons vorreden zu sacroboscos «spahera» (1531) und zum «computus ecclesiasticus» (1538). *Beiträge zur Astronomiegeschichte* **7**, 13–44 (2004).

90. M. Valleriani, B. Federau, O. Nicolaeva, The hidden *Praeceptor*: How Georg Rheticus taught geocentric cosmology to Europe. *Perspect. Sci.* **30**, 1–46 (2022).

91. I. Pantin, "Borrowers and innovators in the printing history of sacrobosco: The case of the "in-octavo" tradition" in *De sphaera of Johannes de Sacrobosco in the Early Modern Period: The Authors of the Commentaries*, M. Valleriani, Ed. (Springer Nature, 2020), pp. 265–312.

92. P. Findlen, *Empires of Knowledge. Scientific Networks in the Early Modern World* (Routledge, 2018).

93. H. Leitão, Sphaera Mundi: *A Ciência na Aula de Esfera. Manuscriptos científicos do Colégio de Santo Antão nas colecções da* BNP (Biblioteca Nacional de Portugal, 2008).

94. B. Anderson, *Imagined Communities. Reflections on the Origin and Spread of Nationalism* (Verso, 1983).

95. F. Kräutli, M. Valleriani, CorpusTracer: A CIDOC database for tracing knowledge networks. *Digit. Scholarsh. Hum.* **33**, 336–346 (2018).

96. K. Simonyan, A. Zisserman, Very deep convolutional networks for large-scale image recognition, in *International Conference on Learning Representations (ICLR)* (2015).

97. M. Weiler, G. Cesa, General E(2)-equivariant steerable CNNs, *Conference on Neural Information Processing Systems (NeurIPS)* (Curran Associates, 2019).

98. J. Kauffmann, K.-R. Müller, G. Montavon, Towards explaining anomalies: A deep Taylor decomposition of one-class models. *Pattern Recognit.* **101**, 107198 (2020).

99. G. Montavon, A. Binder, S. Lapuschkin, W. Samek, K.-R. Müller, "Layer-wise relevance propagation: An overview" in *Explainable AI* (Springer, 2019), vol. 11700 of *Lecture Notes in Computer Science*, pp. 193–209.

100. J. MacQueen, Some methods for classification and analysis of multivariate observations, in *Proceedings of the Fifth Berkeley Symposium on Mathematical Statistics and Probability, Volume 1: Statistics* (University of California Press, 1967), pp. 281–297.

101. J. d. Sacrobosco, P. Melanchthon, *Libellus de Sphaera Iohannis de Sacro Busto. Accessit eiusdem Autoris Computus Ecclesiasticus, Et alia quaedam, in studiosorum gratiam edita. Cum Praefatione Philippi Melanchthonis* (Johann Krafft for Zacharias Schürer & Partners, 1601).

102. N. Germanus, *Ptolemy's Cosmographia* (Ms. membr., lat., sec. XV, cc. I–II, 124, III–IV, Biblioteca Nazionale di Napoli, 1460–1466).

103. R. Walton, *A New and Accurat Map of the World Drawne according to ye truest Descriptions lastest Discoveries & best observations yt have beene made by English or Strangers, London 1626* (The Barry Lawrence Ruderman Map Collection. Stanford Univ. Libraries, 1627).

104. F. Faleiro, *Tratado del Esphera y del arte del marear: con el regimiento de las alturas: con algunas reglas nuevamente escritas muy necessarias* (Juan Cromberger, 1535).

105. P. F. Gehl, "Advertising or fama? Local markets for schoolbooks in sixteenth-century italy" in *Print Culture and Peripheries in Early Modern Europe. A Contribution to the History of Printing and the Book Trade in Small European and Spanish Cities*, B. R. Costas, Ed. (Brill, 2013), pp. 69–100.

106. I. Pantin, "Les problèmes de l'édition des livres scientifiques: l'exemple de guillaume cavellat" in *Le livre dans l'Europe de la Renaissance: Actes du XXVIIIe Colloque international d'Etudes humanistes de Tours*, B. Nationale, Ed. (Promodis, Editions du Cercle de la Librairie, 1998), p. 240–252.

107. A. Ottone, "The giunta's publishing and distributing network and their supply to the european academic market" in *Publishing Sacrobosco's «De sphaera» in Early Modern Europe. Modes of Material and Scientific Exchange*, M. Valleriani, A. Ottone, Eds. (Springer Nature, 2022), pp. 255–288.

108. S. Werner, *Studying Early Printed Books. 1450–1800. A Practical Guide* (Wiley Blackwell, 2019).

109. W. Friedensburg, *Geschichte der Universität Wittenberg* (Niemeyer, 1917).

110. W. Friedensburg, *Urkundenbuch der Universität Wittenberg. Teil 1 (1502–1611)* (Selbstverlag der historischen Kommission, 1920).

111. J. d. Sacrobosco, F. Pifferi, *Sfera di Gio. Sacro Bosco tradotta, e dichiarata da don Francesco Pifferi San Savino Monaco Camaldolense, e Matematico nello Studio de Siena. Misurato Intronato. Al Serensissimo don Cosimo Medici Gran Principe di Toscana. Con nuove aggiunte di molte cose notabili, e varie demostrazioni utili, e dilettevoli. Come nella seguente Tavola si vede* (Silvestro Marchetti, 1604).

112. M. Valleriani, *Galileo Engineer, Boston Studies in the Philosophy of Science* (Springer, 2010).

113. M. Valleriani, C. Sander, "Paratexts, printers, and publishers: Book production in social context" in *Publishing Sacrobosco's «De sphaera» in Early Modern Europe. Modes of Material and Scientific Exchange*, M. Valleriani, A. Ottone, Eds. (Springer, 2022), p. 337–367.

114. C. Rideau-Kikuchi, "Erhard ratdolt's edition of sacrobosco's «tractatus de sphaera:» a new editorial model in venice?" in *Publishing Sacrobosco's «De sphaera» in Early Modern Europe. Modes of Material and Scientific Exchange*, M. Valleriani, A. Ottone, Eds. (Springer Nature, 2022), pp. 61–98.

115. M. Valleriani, F. Kräutli, D. Lockhorst, N. Shlomi, "Vision on vision: Defining similarities among early modern illustrations on cosmology" in *Scientific Visual Representations in History* (Springer, 2023), p. 99–137.

116. M. Valleriani, F. Kräutli, "The necessity of linked data alias thinking big in computational history" in *Person und Wissen. Bilanz und Perspektive* (vdf Hochschulverlag AG, 2022), p. 171–191.

117. H. El-Hajj, M. Zamani, J. Büttner, J. Martinetz, O. Eberle, N. Shlomi, A. Siebold, G. Montavon, K.-R. Müller, H. Kantz, M. Valleriani, An ever-expanding humanities knowledge

graph: The sphaera corpus at the intersection of humanities, data management, and machine learning. *Datenbank-Spektrum: Zeitschrift für Datenbanktechnologien und Information Retrieval* **22**, 153–162 (2022).

118. F. Kräutli, D. Lockhorst, M. Valleriani, Calculating sameness: Identifying early-modern image reuse outside the black box. *Digital Scholarship in the Humanities* **36**, 165–174 (2020).

119. J. de Sacrobosco, J. Regiomontanus, G. von Peuerbach, *Spaerae mundi compendium foeliciter inchoat. Noviciis adolescentibus: ad astronomicam rem publicam capessendam aditum impetrantibus: pro brevi rectoque tramite a vulgari vestigio semoto: Ioannis de Sacro busto sphaericum opusculum una cum additionibus nonnullis littera A sparsim ubi intersertae sint signatis: Contraque cremonensia in planetarum theoricas delyramenta Ioannis de monte regio disputationes tam acuratiss. atque utills. Nec non Georgii purbachii in erundem motus planetarum acuratiss. theoricae: dicatum opus: utili serie contextum: fausto sidere inchoat* (Ottaviano Scoto I, 1490).

120. J. Büttner, "Shooting with ink" in *The Structures of Practical Knowledge*, M. Valleriani, Ed. (Springer Nature, 2017), pp. 115–166.

121. M. Valleriani, From the quadrivium to modern science. *Hist. Sci. Technol.* **16**, 121–132 (2022).

122. G. M. Cooper, Numbers, prognosis, and healing: Galen on medical theory. *J. Wash. Acad. Sci.* **98**, 45–60 (2004).

123. J. de Sacrobosco, C. Clavius, *Christophori Clavii Bambergensis ex Societate Iesu in Sphaeram Ioannis de Sacro Bosco commentarius Nunc tertio ab ipso Auctore recognitus, & plerisque in locis locupletatus. Permissu superiorem* (Domenico Basa, 1585).

124. J. de Sacrobosco, C. Clavius, *Christophori Clavii Bambergensis ex Societate Iesu In Sphaeram Ioannis de Sacro Bosco commentarius, Nunc tertio ab ipso Auctore recognitus, & plerisque in locis locupletatus. Permissu Superiorum* (Giovanni Battista Ciotti, 1591).

125. O. Finé, *Orontii Finei Delphinatis, liberalium disciplinarum professoris regii, protomathesis: Opus varium, ac scitu non minus utile quàm iucundum, nunc primùm in lucem foeliciter emissum. Cuius index universalis, in versa pagina continetur* (Jean Pierre de Tour for Gérard Morrhy, 1532).

126. J. Qiu, Q. Wu, G. Ding, Y. Xu, S. Feng, A survey of machine learning for big data processing. *EURASIP J. Adv. Signal Process.* **2016**, 1–16 (2016).

127. M. Adibuzzaman, P. DeLaurentis, J. Hill, B. Benneyworth, Big data in healthcare – the promises, challenges and opportunities from a research perspective: A case study with a model database. *AMIAAnnu. Symp. Proc.* **2017**, 384–392 (2018).

128. C. Kelly, A. Karthikesalingam, M. Suleyman, G. Corrado, D. King, Key challenges for delivering clinical impact with artificial intelligence. *BMC Med.* **17**, 195 (2019).

129. O. Finé, *De Mundi sphaera, sive Cosmographia, primáve Astronomiae parte, Lib. V* (Simon de Colines, 1542).

130. O. Finé, *Opere...Divise in cinque parti; arimetica, geometria, cosmografia, et orivoli* (Francesco de Franceschi, 1587).

131. J. d. Sacrobosco, F. Giuntini, *Commentaria in Sphæram Ioannis de Sacro Bosco accuratissima* (Philippe Tinghi, 1578).

132. J. d. Sacrobosco, P. Melanchthon, *Ioannis de Sacrobusto libellus de sphaera* (Johann Krafft, 1550).

133. W. Abdulla, Mask R-CNN for object detection and instance segmentation on keras and TensorFlow, (2017); https://github.com/matterport/Mask_RCNN.

134. B. Sun, J. Feng, K. Saenko, Return of frustratingly easy domain adaptation, in *Proceedings of the Thirtieth AAAI Conference on Artificial Intelligence, AAAI'16* (AAAI Press, 2016), pp. 2058–2065.

135. H. Zhao, R. T. D. Combes, K. Zhang, G. Gordon, On learning invariant representations for domain adaptation, in *Proceedings of the 36th International Conference on Machine Learning*, K. Chaudhuri, R. Salakhutdinov, Eds. (PMLR, 2019), vol. 97 of *Proceedings of Machine Learning Research*, pp. 7523–7532.

136. J. Wang, C. Lan, C. Liu, Y. Ouyang, T. Qin, W. Lu, Y. Chen, W. Zeng, P. S. Yu, Generalizing to unseen domains: A survey on domain generalization. *IEEE Trans. Knowl. Data Eng.* **35**, 8052–8072 (2023).

137. L. Andéol, Y. Kawakami, Y. Wada, T. Kanamori, K.-R. Müller, G. Montavon, Learning domain invariant representations by joint wasserstein distance minimization. *Neural Netw.* **167**, 233–243 (2023).

138. M. Y. Yang, B. Rosenhahn, V. Murino, *Multimodal Scene Understanding: Algorithms, Applications and Deep Learning* (Academic Press, Inc., ed. 1, 2019).

139. A. Paszke, S. Gross, F. Massa, A. Lerer, J. Bradbury, G. Chanan, T. Killeen, Z. Lin, N. Gimelshein, L. Antiga, A. Desmaison, A. Kopf, E. Yang, Z. DeVito, M. Raison, A. Tejani, S. Chilamkurthy, B. Steiner, L. Fang, J. Bai, S. Chintala, "Pytorch: An imperative style, high-performance deep learning library" in *Advances in Neural Information Processing Systems 32*, H. Wallach, H. Larochelle, A. Beygelzimer, F. d'Alché-Buc, E. Fox, R. Garnett, Eds. (Curran Associates Inc., 2019), pp. 8024–8035.

140. E. Simoncelli, W. Freeman, The steerable pyramid: A flexible architecture for multiscale derivative computation, in *Proceedings, International Conference on Image Processing* (IEEE, 1995), vol. 3, pp. 444–447.

141. Y. Zheng, H. Li, D. Doermann, Machine printed text and handwriting identification in noisy document images. *IEEE Trans. Pattern Anal. Mach. Intell.* **26**, 337–353 (2004).

142. J. Martínek, L. Lenc, P. Král, Building an efficient OCR system for historical documents with little training data. *Neural Comput. Appl.* **32**, 17209–17227 (2020).

143. L. Lyu, M. Koutraki, M. Krickl, B. Fetahu, Neural OCR post-hoc correction of historical corpora. *Trans. Assoc. Comput. Linguist.* **9**, 479–493 (2021).

144. D. H. Diaz, S. Qin, R. R. Ingle, Y. Fujii, A. Bissacco, Rethinking text line recognition models. arXiv:2104.07787 [cs.CV] (2021).

145. A. Sulaiman, K. Omar, M. F. Nasrudin, Degraded historical document binarization: A review on issues, challenges, techniques, and future directions. *J. Imaging* **5**, (2019).

146. D. Bamman, D. Smith, Extracting two thousand years of latin from a million book library. *J. Comput. Cult. Herit.* **5**, 1–13 (2012).

147. S. Lapuschkin, S. Wäldchen, A. Binder, G. Montavon, W. Samek, K.-R. Müller, Unmasking Clever Hans predictors and assessing what machines really learn. *Nat. Commun.* **10**, 1096 (2019).

148. W. Samek, A. Binder, G. Montavon, S. Lapuschkin, K.-R. Müller, Evaluating the visualization of what a deep neural network has learned. *IEEE Trans. Neural Netw. Learning Syst.* **28**, 2660–2673 (2017).

149. W. Samek, G. Montavon, A. Vedaldi, L. K. Hansen, K.-R. Müller, Eds., *Explainable AI: Interpreting, Explaining and Visualizing Deep Learning* (Springer, 2019), vol. 11700 of *Lecture Notes in Computer Science*.

150. E. Tjoa, C. Guan, A survey on explainable artificial intelligence (XAI): Toward medical XAI. *IEEE Trans. Neural Netw. Learn. Syst.* **32**, 4793–4813 (2021).

151. T. Schnake, O. Eberle, J. Lederer, S. Nakajima, K. T. Schütt, K.-R. Müller, G. Montavon, Higher-order explanations of graph neural networks via relevant walks. *IEEE Trans. Pattern Anal. Mach. Intell.* **44**, 7581–7596 (2022).

152. M. Rupp, A. Tkatchenko, K.-R. Müller, O. A. von Lilienfeld, Fast and accurate modeling of molecular atomization energies with machine learning. *Phys. Rev. Lett.* **108**, 058301 (2012).

153. K. T. Schütt, H. E. Sauceda, P.-J. Kindermans, A. Tkatchenko, K.-R. Müller, SchNet—A deep learning architecture for molecules and materials. *J. Chem. Phys.* **148**, 241722 (2018).

154. K. T. Schütt, F. Arbabzadah, S. Chmiela, K. R. Müller, A. Tkatchenko, Quantum-chemical insights from deep tensor neural networks. *Nat. Commun.* **8**, 13890 (2017).

155. O. T. Unke, S. Chmiela, H. E. Sauceda, M. Gastegger, I. Poltavsky, K. T. Schütt, A. Tkatchenko, K.-R. Müller, Machine learning force fields. *Chem. Rev.* **121**, 10142–10186 (2021).

156. C. J. Shallue, A. Vanderburg, Identifying exoplanets with deep learning: A five-planet resonant chain around kepler-80 and an eighth planet around kepler-90. *Astron. J* **155**, 94 (2018).

157. H. Valizadegan, M. Martinho, L. Wilkens, J. Jenkins, J. Smith, D. Caldwell, P. Gerum, N. Walia, K. Hausknecht, N. Lubin, J. Twicken, N. Oza, Exominer: A highly accurate and explainable deep learning classifier that validates 200+ new exoplanets. *Astrophys. J* **926**, 120 (2022).

158. G. Sumbul, M. Charfuelan, B. Demir, V. Markl, BigEarthNet: A large-scale benchmark archive for remote sensing image understanding, in *IGARSS 2019–2019 IEEE International Geoscience and Remote Sensing Symposium* (IEEE, 2019), pp. 5901–5904.

159. J. Runge, P. Nowack, M. Kretschmer, S. Flaxman, D. Sejdinovic, Detecting and quantifying causal associations in large nonlinear time series datasets. *Sci. Adv.* **5**, eaau4996 (2019).

160. B. A. Toms, E. A. Barnes, I. Ebert-Uphoff, Physically interpretable neural networks for the geosciences: Applications to earth system variability. *J. Adv. Model. Earth Syst.* **12**, e2019MS002002 (2020).

161. F. Klauschen, K.-R. Müller, A. Binder, M. Bockmayr, M. Hägele, P. Seegerer, S. Wienert, G. Pruneri, S. Maria, S. Badve, S. Michiels, T. Nielsen, S. Adams, P. Savas, F. Symmans, S. Willis, T. Gruosso, M. Park, B. Haibe-Kains, C. Denkert, Scoring of tumor-infiltrating

lymphocytes: From visual estimation to machine learning. *Semin. Cancer Biol.* **52**, 151–157 (2018).

162. A. Binder, M. Bockmayr, M. Hägele, S. Wienert, D. Heim, K. Hellweg, M. Ishii, A. Stenzinger, A. Hocke, C. Denkert, K. R. Müller, F. Klauschen, Morphological and molecular breast cancer profiling through explainable machine learning. *Nat. Mach. Intell.* **3**, 355–366 (2021).

163. U. Güçlü, M. A. J. van Gerven, Deep neural networks reveal a gradient in the complexity of neural representations across the ventral stream. *J. Neurosci.* **35**, 10005–10014 (2015).

164. S. A. Cadena, G. H. Denfield, E. Y. Walker, L. A. Gatys, A. S. Tolias, M. Bethge, A. S. Ecker, Deep convolutional models improve predictions of macaque v1 responses to natural images. *PLoS Comput. Biol.* **15**, e1006897 (2019).

165. W. J. Neumann, R. S. Turner, B. Blankertz, T. Mitchell, A. A. Kühn, R. M. Richardson, Toward electrophysiology-based intelligent adaptive deep brain stimulation for movement disorders. *Neurotherapeutics* **16**, 105–118 (2019).

166. M. W. Mathis, A. Mathis, Deep learning tools for the measurement of animal behavior in neuroscience. *Curr. Opin. Neurobiol.* **60**, 1–11 (2020).

167. R. Roscher, B. Bohn, M. F. Duarte, J. Garcke, Explainable machine learning for scientific insights and discoveries. *IEEE Access* **8**, 42200–42216 (2020).

168. S. Ranathunga, E.-S. A. Lee, M. Prifti Skenduli, R. Shekhar, M. Alam, R. Kaur, Neural machine translation for low-resource languages: A survey. *ACM Comput Surv* **55**, 1–37 (2023).

169. A. Pine, D. Wells, N. Brinklow, P. Littell, K. Richmond, Requirements and motivations of low-resource speech synthesis for language revitalization, in *Proceedings of the 60th Annual Meeting of the Association for Computational Linguistics (Volume 1: Long Papers)* (Association for Computational Linguistics, 2022), pp. 7346–7359.

170. L. A. Gatys, A. S. Ecker, M. Bethge, Image style transfer using convolutional neural networks, in *2016 IEEE Conference on Computer Vision and Pattern Recognition (CVPR)* (IEEE, 2016), pp. 2414–2423.

171. S.-G. Lee, E.-Y. Cha, Style classification and visualization of art painting's genre using self-organizing maps. *HCIS* **6**, 7 (2016).

172. B. Seguin, C. Striolo, I. diLenardo, F. Kaplan, "Visual link retrieval in a database of paintings" in *Computer Vision–ECCV 2016 Workshops*, G. Hua, H. Jégou, Eds. (Springer International Publishing, 2016), pp. 753–767.

173. S. Lang, B. Ommer, Attesting similarity: Supporting the organization and study of art image collections with computer vision. *Digit. Scholarsh. Hum.* **33**, 845–856 (2018).

174. M. Panagopoulos, C. Papaodysseus, P. Rousopoulos, D. Dafi, S. Tracy, Automatic writer identification of ancient Greek inscriptions. *IEEE Trans. Pattern Anal. Mach. Intell.* **31**, 1404–1414 (2009).

175. O. Vane, Using data visualisation to tell stories about cultural collections, in *Proceedings of the 2017 CHI Conference Extended Abstracts on Human Factors in Computing Systems, CHI EA '17* (Association for Computing Machinery, 2017), p. 335–339.

176. I. Schlag, O. Arandjelovic, Ancient roman coin recognition in the wild using deep learning based recognition of artistically depicted face profiles, in *2017 IEEE International Conference on Computer Vision Workshops (ICCVW)* (IEEE, 2017), pp. 2898–2906.

177. X. Shen, A. A. Efros, M. Aubry, Discovering visual patterns in art collections with spatially-consistent feature learning, in *Proceedings IEEE Conf. on Computer Vision and Pattern Recognition (CVPR)* (IEEE, 2019).

178. T. R. Tangherlini, P. Leonard, Trawling in the sea of the great unread: Sub-corpus topic modeling and humanities research. *Poetics* **41**, 725–749 (2013).

179. M. L. Jockers, D. Mimno, Significant themes in 19th-century literature. *Poetics* **41**, 750–769 (2013).

180. C. Schöch, Topic modeling genre: An exploration of french classical and enlightenment drama. arXiv:2103.13019 [cs.CL] (2021).

181. M. Koppel, M. Michaely, A. Tal, Reconstructing ancient literary texts from noisy manuscripts, in *Proceedings of the Fifth Workshop on Computational Linguistics for Literature* (Association for Computational Linguistics, 2016), pp. 40–46.

182. N. Yadav, H. Joglekar, R. P. N. Rao, M. N. Vahia, R. Adhikari, I. Mahadevan, Statistical analysis of the indus script using n-grams. *PLOS ONE* **5**, –e9506 (2010).

183. J. Luo, Y. Cao, R. Barzilay, Neural decipherment via minimum-cost flow: From Ugaritic to Linear B, in *Proceedings of the 57th Annual Meeting of the Association for Computational Linguistics* (Association for Computational Linguistics, 2019), pp. 3146–3155.

184. J. Luo, F. Hartmann, E. Santus, R. Barzilay, Y. Cao, Deciphering undersegmented ancient scripts using phonetic prior. *Trans. Assoc. Comput. Linguist.* **9**, 69–81 (2021).

185. C. M. Bishop, *Neural networks for pattern recognition* (Oxford Univ. Press, 1995).

186. C. Tan, F. Sun, T. Kong, W. Zhang, C. Yang, C. Liu, A survey on deep transfer learning, in *Artificial Neural Networks and Machine Learning–ICANN 2018: 27th International Conference on Artificial Neural Networks, Rhodes, Greece, October 4–7, 2018, Proceedings, Part III 27* (Springer, 2018), pp. 270–279.

187. Y. Wang, Q. Yao, J. T. Kwok, L. M. Ni, Generalizing from a few examples: A survey on few-shot learning. *ACM Comput. Surveys* **53**, 1–34 (2020).

188. C. Bekiari, G. Bruseke, M. Doerr, C.-E. Ore, S. Stead, A. Velios, Definition of the CIDOC conceptual reference model v7.1.1, *The CIDOC Conceptual Reference Model Special Interest Group* (ICOM, 2021).

189. C. Bekiari, M. Doerr, P. L. Boeuf, P. Riva, *Definition of FRBRoo: A conceptual model for bibliographic information in object-oriented formalism* (IFLA Repository, 2015).

190. C. Meghini, M. Doerr, A first-order logic expression of the cidoc conceptual reference model. *Int. J. Metadata Semant. Ontologies* **13**, 131–149 (2018).

191. F. Kräutli, E. Chen, M. Valleriani, “Linked data strategies for conserving digital research outputs” in *Information and Knowledge Organisation in Digital Humanities* (Routledge, 2021), pp. 206–224.

192. E. Poole, The computer in determining stemmatic relationships. *Comput. Hum.* **8**, 207–216 (1974).

193. J.-B. Camps, F. Cafiero, “Stemmatology: An R package for the computer-assisted analysis of textual traditions” in *Corpus-Based Research in the Humanities CRH-2* (Gerastree Edition, 2018).

194. J. Savoy, *Machine Learning Methods for Stylometry: Authorship Attribution and Author Profiling* (Springer International Publishing, 2020).

195. K. Manders, “The euclidean diagram” in *The Philosophy of Mathematical Practice*, P. Mancosu, Ed. (Oxford Univ. Press, 2008), pp. 80–133.

196. J. Franklin, “Diagrammatic reasoning and modelling in the imagination: The secret weapons of the scientific revolution” in *1543 and All That: Image and Word, Change and Continuity in the Proto-Scientific Revolution*, G. Freeland, A. Corones, Eds. (Kluwer Academic Publishers, 2000).

197. S.-J. Shin, O. Lemon, J. Mumma, “Diagrams” in *The Stanford Encyclopedia of Philosophy*, E. N. Zalta, Ed. (Metaphysics Research Lab, Stanford University, 2018), Winter 2018 edition.

198. G. Priest, S. De Toffoli, P. Findlen, Tools of reason: The practice of scientific diagramming from antiquity to the present. *Endeavour* **42**, 49–59 (2018).

199. S. D. Toffoli, What are mathematical diagrams? *Synthese* **200**, 1–29 (2022).

200. S. Kalleli, T. Scott, A. Ségolène, G. Samuel, H. Mathieu, A. Mathieu, Editing and analysing historical astronomical diagrams wit artificial intelligence, in *Proceedings of the IAMAHA 2023, Nice, France* (Ècole des Ponts, ParisTech, 2023).

201. S. Brausch, G. Graßhoff, Machine learning for the history of ideas. *Future Hum.* **1**, e6 (2023).

202. L. van der Maaten, G. Hinton, Visualizing data using t-SNE. *J. Mach. Learn. Res.* **9**, 2579–2605 (2008).

203. B. Kang, D. García García, J. Lijffijt, R. Santos-Rodríguez, T. De Bie, Conditional t-SNE: More informative t-sne embeddings. *Mach. Learn* **110**, 2905–2940 (2021).

204. F. Anowar, S. Sadaoui, B. Selim, Conceptual and empirical comparison of dimensionality reduction algorithms (PCA, KPCA, LDA, MSD, SVD, LLE, ISOMAP, LE, ICA, t-SNE). *Comput Sci Rev* **40**, 100378 (2021).

205. I. Pantin, "Oronce finé mathématiien et homme du livre: la pratique éditoriale comme *moteur d'évolution*" in *Mise en forme des savoirs à la Renaissance. À la croisée des idées, des techniques et des public*, I. Pantin, G. Péoux, Eds. (Armand Colin, 2013), pp. 19–40.

206. O. Finé, *Sphaera mundi, sive cosmographia quinque recèns auctis & emendatis absoluta* (Michel Vascosan, 1551).

207. O. Finé, *Sphaera mundi, sive cosmographia quinque libris recèns auctis & emendatis absoluta* (Michel Vascosan, 1552).

208. O. Finé, *Orontii Finaei Delphinatis, regii mathematicarum Lutetiae professoris, de mundi sphaera, sive cosmographia, libri V* (Michel Vascosan, 1555).

209. O. Finé, *Le sphere du monde, proprement ditte cosmographie, composee nouvellement en francois, & divisee en cinq livres* (Michel Vascosan, 1551).

210. O. Finé, *Le sphere du monde, proprement ditte cosmographie, composee nouvellement en francois, & divisee en cinq livres* (Michel Vascosan, 1552).

211. J. d. Sacrobosco, H. Beyer, *Quaestiones novae, in libellum de Sphaera Iohannis de Sacro Busto, in gratiam studiosae inventutis collectae ab Hartmanno Bavaro* (Peter Braubach, 1549).

212. J. d. Sacrobosco, H. Beyer, *Quaestiones novae in libellum de sphaera Ioannis de Sacro Busto, in gratiam studiosae iuventutis collectae ab Hartmanno Beyer, recognitae & plaerisque in locis auctae* (Peter Braubach, 1549).

213. J. d. Sacrobosco, H. Beyer, *Quaestiones novae, in libellum de Sphaera Iohannis de Sacro Busto, in gratiam studiosae inventutis collectae ab Ariele Bicardo* (Peter Braubach, 1549).

214. J. d. Sacrobosco, H. Beyer, *Quaestiones in libellum De sphaera Ioannis de Sacro Busto, in gratiam studiosae iuuentutis collectae ab Hartmanno Beyer & nunc denuo recognitae* (Peter Braubach,1552).

215. J. d. Sacrobosco, H. Beyer, *Qvaestiones in libellum de sphaera Ioannis de Sacro Busto, in gratiam studiosae iuventutis collectae ab Hartmanno Beyer, & nunc denuo recognitae* (Peter Braubach, 1556).

216. J. d. Sacrobosco, H. Beyer, *Quaestiones in libellum de sphaera Ioannis de Sacro Busto, in gratiam studiosae iuventutis olim in Academia Vuitebergensi collectae, per Hartmannum Beyer, nunc emendatae & auctae* (Peter Braubach, 1561).

217. J. d. Sacrobosco, H. Beyer, *Quaestiones in libellum de sphaera Ioannis de Sacro Busto, in gratiam studiosae iuventutis olim in Acadaemia Vuitebergensi collectae, per Hartmannum Beyer, nunc emendatae & auctae* (Peter Braubach, 1563).

218. J. d. Sacrobosco, H. Beyer, *Quaestiones. In libellum de Sphaera Ioannis de Sacro Busto, in gratiam studiosae iuventutis olim in Academia Vuitebergensi collectae: Nunc vero denuo revisae emendatae & auctae. Per M. Hartmannum Beyer* (Heirs of Peter Braubach, 1571).

219. J. d. Sacrobosco, H. Beyer, *Quaestiones in libellum de Sphaera Ioannis de Sacro busto, in gratiam studiosae iuventutis olim in Academia, Vuitebergensi collectae, per Hartmannum Beyer, nunc emendatae & auctae* (Peter Braubach, 1560).

220. J. d. Sacrobosco, H. Beyer, *Quaestiones in libellum de sphaera Ioannis de Sacro Busto, in gratiam studiosae iuventutis olim in Academia Vitebergensi collectae, per Hartmannum Beyer, nunc emendatae & auctae* (Peter Braubach, 1560).

221. S. Wilhelm Adolf, *Isagoge sphaerica methodice proposita a Guilhelmo Adolpho Scribonio Marpurgensi* (André Wechel, 1580).

222. S. Wilhelm Adolf, *Isagoge sphaerica methodice proposita a Gulielmo Adolpho Scribonio Marpurgensi* (Johann Wechel, 1587).

223. S. Wilhelm Adolf, Z. Palthenius, *Isagoge sphaerica Guilielmi Adolpho Scribonii, in gratiam studiosae iuventutis notis & explicationibus illustrata per M. Zachariam Palthenium Fridbergensem* (Johann Wechel, 1593).

224. S. Wilhelm Adolf, Z. Palthenius, *Isagoge sphaerica Gulielmi Adolphi Scribonii, in gratiam studiosae iuventutis notis & explicationibus illustrata per M.* Zachariam Palthenium Fridbergensem (Jonas Rosa, 1600).

225. I. C. Hennen, "Printers, booksellers, and bookbinders in wittenberg in the sixteenth century: Real estate, vicinity, political, and cultural activities" in *Publishing Sacrobosco's De sphaera in Early Modern Europe. Modes of Material and Scientific Exchange*, M. Valleriani, A. Ottone, Eds. (Springer, 2022), pp. 99–154.

226. J. de Sacrobosco, P. Melanchthon, *Liber Iohannis de Sacro Busto, de Sphaera. Addita est praefatio in eundem librum Philippi Melanchthonis ad Simonem Gryneum* (Joseph Klug, 1531).

227. D. Shcheglov, Ptolemy's system 0f seven climata and eratosthenes geography. *Geographia Antiqua* **13**, 21–37 (2004).

228. G. Graßhoff, "Living according to the seasons" in *Knowledge, Text and Practice in Ancient Technical Writing* (Cambridge Univ. Press, 2017), pp. 200–216.

229. E. Honigmann, F. Sezgin, *Die sieben Klimata und die Poleis Episemoi : eine Untersuchung zur Geschichte der Geographie und Astrologie im Altertum und Mittelalter* (Institute for the History of Arabic-Islamic Science, 1992).

230. D. R. Dicks, The ΚΛΙΜΑΤΑ in Greek geography. *Class. Q* **5**, 248–255 (1955).

231. M. G. Nickiforov, Analysis of the calendar C. Ptolemy "Phases of the fixed stars". *Bulg. Astron. J* **20**, 68 (2014).

232. K. Peucer, *Elementa doctrinae de circulis coelestibus, et primo motu, recognita et correcta, autore Casparo Peucero* (Johann Krafft the Elder, 1558).

233. M. Cortés, *Breve compendio de la sphera y de la arte de navegar, con nueuos instrumentos y reglas, exemplificado con muy subtiles demonstraciones: compuesto por Martin Cortes natural de burjalaros en el reyno de Aragon y de presente vezino de la ciudad de Cadiz: dirigido al invictissimo Monarcha Carlo Quinto Rey de las Hespanas etc. Senor Nuestro* (António Alvares,1556).

234. H. Witekind, *De sphaera mundi: Et Témporis ratione apud Christianos. Hermanni VVitekindi* (Matthäus Harnisch, 1590).

235. S. Dietrich, *Novae quaestiones sphaericae, hoc est, de circulis coelestibus & primo mobili, in gratiam studiosae iuventutis scriptae, a M. Sebastiano Theodorico Vuinshemio. Mathematum Professore* (Matthaeus Welack, 1591).

236. J. d. Sacrobosco, *Opusculum Johannis de sacro busto spericum cum notabili commento atque figuris textum declarantibus utilissimis* (Martin Landsberg, 1495).

237. F. Barozzi, *Cosmographia in quatuor libros distributa, summo ordine, miraque facilitate, ac brevitate ad Magnam Ptolemaei Mathematicam Constructionem, ad universamque Astrologiam instituens: Francisco Barocio, Iacobi Filio, Patritio Veneto autore. Cum Prefatione eiusdem Authoris, in qua perfecta quidem Astrologiae Divisio, & enarratio Aurorum illustrium, & voluminum ab eis conscriptorum in singulis Astrologiae partibus habetur: Ioannis de Sacrobosco verò 84 errores, & alij permulti*

suorum expositorum, & sectatorum ostenduntur, rationibusque redarguuntur. Precesserunt etiam quaedem Communia Mathematica, necnon Arithmetica & Geometrica principia, nonnulleque Propositiones, de quibus in toto opere saepe sit mentio: Ac demum locupletissimus Index eorum, que ipsa Cosmographia continentur. Omnia nuper in hac secunda editione ab ipso Autore diligenter recognita, multisque in locis aucta (Grazioso Percacino, 1598).

238. I. Pantin, "Lire le ciel dans les poèmes anciens. le De ortu poetico et la pédagogie de melanchthon" in *'Une honnête curiosité de s'enquérir de toutes choses'. Mélanges en l'honneur d'Olivier Millet*, M. C. de Ribes, S. Dembruk, D. Fliege, V. Oberliessen, Eds. (Droz, 2021), pp. 373–384.

239. T. Blebel, *De sphaera et primis astronomiae rudimentis libellus ad usum Scholarum maximè accomodatus: accurata methodo & brevitate conscriptus, ac denuo editus. A M. Thoma Blebelio Budissino* (Johann Krafft, 1582).

240. M. H. Close, Hipparchus and the precession of the equinoxes. *Proc. R. Irish Acad.* **6**, 450–456 (1900).

241. M. T. R. G. Gavilanes, A. Mollgaard, T. Yasseri, The memory remains: Understanding collective memory in the digital age. *Sci. Adv.* **3**, e1602368 (2017).
